# Evaluation of the Persistence of Higher-Order Strand Symmetry in Genomic Sequences by Novel Word Symmetry Distance Analysis

**DOI:** 10.3389/fgene.2019.00148

**Published:** 2019-03-07

**Authors:** Bi Huang, Li-Fang Huang, Shang-Hong Zhang

**Affiliations:** Key Laboratory of Gene Engineering of Ministry of Education, Biotechnology Research Center, Sun Yat-sen University, Guangzhou, China

**Keywords:** the second parity rule, higher-order oligonucleotide, whole-genome sequences, frequency analysis, word symmetry distance (*WSD*)

## Abstract

For the ubiquitous phenomenon of strand symmetry, it has been shown that it may persist for higher-order oligonucleotides. However, there is no consensus about to what extent (order of oligonucleotides or length of words) strand symmetry still persists. To determine the extent of strand symmetry in genomic sequences is critically important for the further understanding of the phenomenon. Based on previous studies, we have developed an algorithm for the novel word symmetry distance analysis. We applied it to evaluate the higher-order strand symmetry for 206 archaeal genomes and 2,659 bacterial genomes. Our results show that the new approach could provide a clear-cut criterion to determine the extent of strand symmetry for a group of genomes or individual genomes. According to the new measure, strand symmetry would tend to persist for up to 8-mers in archaeal genomes, and up to 9-mers in bacterial genomes. And the persistence may vary from 6- to 9-mers in individual genomes. Moreover, higher-order strand symmetry would tend to positively correlate with GC content and mononucleotide symmetry levels of genomic sequences. The variations of higher-order strand symmetry among genomes would indicate that strand symmetry itself may not be strictly relevant to biological functions, which would provide some insights into the origin and evolution of the phenomenon.

## Introduction

Strand symmetry, also called the second parity rule, exists in almost all modern cellular genomes (genomes in living organisms nowadays on Earth). It is the phenomenon in which the numbers of occurrence of individual nucleotides and oligonucleotides (words) match well and exclusively with those of their respective reverse complements (complementary words) in each genomic DNA strand of sufficient length (e.g., for tetranucleotide GTCA vs. TGAC; for review see Baisnée et al., 2002; Forsdyke and Bell, 2004; Albrecht-Buehler, 2006; Zhang and Huang, 2010; Afreixo et al., 2016; Shporer et al., 2016). As an important feature of genome compositional structures, strand symmetry may persist for higher-order oligonucleotides, while the degree of symmetry decreases with increasing order (see Zhang and Huang, 2008 for example). Moreover, higher-order strand symmetry could not be explained solely by mononucleotide or lower-order oligonucleotide symmetry. For the further understanding of the origin and evolution of the phenomenon, it is essential to figure out to what extent (order of oligonucleotides or length of words) strand symmetry still persists.

Qi and Cuticchia (2001) showed that strand symmetry may persist for oligonucleotides up to 10 nt long in some prokaryotic genomes and eukaryotic chromosomes, based on the analysis of frequencies of some complementary pairs (each consisting of a word and its complementary word). On the other hand, Afreixo et al. (2013) concluded that strand symmetry would be statistically significant for word lengths up to 6 nt in the human genome by equivalence tests and word symmetry distance analysis. Employing some of Afreixo et al.'s data, Zhang (2015) argued that strand symmetry would persist for oligonucleotides up to 9 nt in the human genome. Apparently, there is no consensus for the issue. The extent of strand symmetry must be evaluated with all the complementary pairs, which was not the case in Qi and Cuticchia (2001). Also, the use of equivalence tests would not be fully justified, while the word symmetry distance analysis without taking into account the spacing (absolute distance) that separates the two words of each complementary pair in the word frequency arrangement seems inadequate (Zhang, 2015).

To determine the extent of strand symmetry, some rational criteria must be established. One logical consideration is that if strand symmetry persists for oligonucleotides of length *k* (*k*-mers) in a sequence, the complementary pairs of *k*-mers should, respectively, be more similar in intra-pair frequency compared with corresponding non-complementary pairs. In other words, complementary pairs of *k*-mers must stand out in terms of intra-pair frequency similarity for strand symmetry to persist for *k*-mers in the sequence. In this respect, one may compare in terms of intra-pair frequency similarity/difference all the complementary pairs vs. corresponding non-complementary pairs, especially vs. nearly complementary pairs and nearly identical pairs. The latter two kinds of pairs are most useful for the comparison, because nearly complementary pairs (the same as a complementary pair except one change to another nucleotide of the same GC content anywhere in the word pair, see also Zhang and Huang, 2010) are most closely related to complementary pairs, while nearly identical pairs (each consisting of two otherwise identical words except a single change to another nucleotide of the same GC content anywhere in the word pair) are quite similar by chance alone in intra-pair frequency if strand symmetry exists for mononucleotides. However, there would be enormous or even too many pairs to handle for higher-order oligonucleotides (e.g., 8-, 9-, and 10-mers), which would make the comparison burdensome and computationally intensive.

A viable alternative to the all-against-all comparison is an approach employing also word frequency arrangement as in Afreixo et al. (2013), with random sequences generated according to the nucleotide composition of genomic sequences to be studied as controls (see Method for details). However, unlike the word symmetry distance analysis by Afreixo et al. (2013) that counts only the number of moves in the word frequency rearrangement, the new approach calculates also the absolute distance (step) in each move. The strategy is based on the consideration that: (1) words of each complementary pair, because of frequency similarity, should in general be significantly closer to each other compared to words of any corresponding non-complementary pair (consisting of two words with the same length and GC content as the complementary pair) in the high-to-low frequency arrangement for genomic sequences if strand symmetry persists; and (2) the spacing in the frequency arrangement for words of a complementary pair, for words of any of its corresponding nearly complementary pairs and nearly identical pairs, and even for words of any other corresponding non-complementary pair would not be considerably different from one another for the random sequences generated because of frequency similarity by chance of those words. As strand symmetry declines for *k*-mers, genomic sequences would become somewhat indistinguishable from random sequences in terms of word spacing of *k*-mers in the frequency arrangement. Words of a complementary pair would no longer be closer to each other compared to words of any corresponding non-complementary pair in the frequency arrangement when strand symmetry no longer persists. All things considered, the word spacing, reflecting the word frequency difference, always counts and could not be ignored in the assessment of strand symmetry.

In Afreixo et al.'s study (2013), the frequency difference was regarded as the same (one move) for words of every complementary pair separated by ≥1 other word in the arrangement. The information about word spacing would be lost, thus inadequate in the evaluation of the persistence of strand symmetry. On the other hand, the new approach, based on word arrangement as well as word spacing, would provide an appropriate measure for the intra-pair frequency similarity/difference of complementary pairs in the context of non-complementary pairs. And the method would be more adequate in the study of the phenomenon of strand symmetry.

In the present work, we developed an algorithm for the novel word symmetry distance analysis. We applied our approach to evaluate the higher-order strand symmetry in the genomes of more than 2,000 species of archaea and bacteria. We compared also our metric with that of Afreixo et al. The results show that our approach just meets the need to discriminate the difference between complementary pairs vs. non-complementary pairs for the purpose of evaluating the persistence of strand symmetry. It provides a new and clear-cut measure to determine the extent of strand symmetry for a group of genomes or individual genomes (including the human genome, see also Discussion), which may vary from 6- to 9-mers. Our results would shed new light on the origin and evolution of the phenomenon of strand symmetry.

## Materials and Methods

### Whole-Genome Sequences Analyzed and Their Controls

We downloaded the whole-genome sequences of all species of prokaryotes including archaea and bacteria that were available as of April 2017, along with the information about their taxonomic status, from the NCBI (https://www.ncbi.nlm.nih.gov/genome/browse/). For the species that have two or more strains or subspecies whose genomes have been sequenced, in principle only one was taken randomly from each species. If there is a considerable difference of genomic GC content between individual strains or subspecies of a species, we selected also the strains or subspecies whose GC content is different from that of others by at least 1% (see also Zhang and Wang, 2012; Zhang et al., 2013). In addition, we replaced any genomic sequence containing more than 100 undetermined nucleotides with a better quality sequence from the same species if possible. In total, 206 complete archaeal genomes and 2,659 complete bacterial genomes were analyzed in this study. The complete list, accession numbers, and other information of these genomes are available in Supplementary Material 1.

As controls, we used 1 or 30 random sequences for each genome, depending on the purpose of the analysis (as group or individually). The random sequences were generated according to the exact mononucleotide composition of their corresponding genomic sequences, i.e., with the same numbers of A, C, G, and T, respectively, and consequently the same length, as the real genome. That would be equivalent to shuffling sufficiently the genomic sequence concerned.

### Calculation of Oligonucleotide Occurrence Frequencies

We calculated the occurrence frequencies of oligonucleotides at orders 1–10 for the complete genomes and their corresponding random sequences. Overlapping oligonucleotides were counted for a chromosome (see also Albrecht-Buehler, 2006; Zhang and Huang, 2008). Each chromosome was analyzed separately for genomes with two or more ones. Counts from all chromosomes of a genome were compiled, and occurrence frequencies of oligonucleotides at orders 1–10 for the genome were calculated, respectively, from those counts.

### Calculation of Strand Symmetry Indexes

To calculate the strand symmetry indexes for *k*-mers of a sequence, we first obtained the occurrence frequencies of all its *k*-mers. Perfect strand symmetry of order *k* is that every *k*-mer has the same frequency as its reverse complement. For any given sequence, we measured the strand symmetry at order *k* as the similarity between frequencies of all possible *k*-mers (*f*_*i*_, *i* = 1, 2, 3, …, *n*, where *n* is 4^*k*^) and those of their respective reverse complements (*f*_*i*_′, *i* = 1, 2, 3, …, *n*) in the sequence. To figure out the similarity level, we developed two symmetry indexes as follows:

(1) $$\begin{array}{r} S_{1}=1-\frac{\sum_{i}|f_{i}-f_{i}^{{}^{\prime }}|}{\sum_{i}\left(f_{i}+f_{i}^{{}^{\prime }}\right)} \end{array}$$

(2) $$\begin{array}{r} S_{2}=1-\frac{1}{M}\underset{i}{\sum }\frac{\left|f_{i}-f_{i}^{{}^{\prime }}\right|}{f_{i}+f_{i}^{{}^{\prime }}} \end{array}$$

*S*_1_ is equivalent to the index suggested by Baisnée et al. (2002), in which the word frequency difference for each complementary pair is weighed by the total frequency of all words. *S*_2_ is an index proposed in this paper, in which each pair is given the same weight regardless of its frequencies (*M* being the number of pairs involved in the calculation; each complementary pair with two different words being considered as two pairs if calculated twice, see below). It may be regarded as a control of *S*_1_. We employed the two symmetry indexes to investigate whether they, taken together, may provide more information about the extent of higher-order strand symmetry. In the calculation of both symmetry indexes in this study, we considered only words each of which has a reverse complement different from itself (the frequencies of words that are identical to their respective reverse complements (e.g., ACGT) were not used in the calculation). Moreover, if both words of a pair were absent (*f* = *f* ′ = 0), the pair was not counted either. For convenience and to avoid omission, the frequencies of all the words considered for the symmetry indexes were regarded as *f*_*i*_, those of their reverse complements as *f*_*i*_′. Accordingly, the word frequency difference for each complementary pair with two different words was calculated twice (e.g., |*f*_*AC*_—*f*_*GT*_| and |*f*_*GT*_—*f*_*AC*_|) and compensated in the denominator of the above two equations.

We calculated *S*_1_ and *S*_2_ at orders 1 to 10 for the complete genomes and their corresponding random sequences.

### Calculation of Word Symmetry Distance

To calculate the word symmetry distance of *k*-mers for a sequence, we first arranged all possible *k*-mers from highest to lowest in terms of their frequencies in the sequence to obtain arrangement *A*_1_(*k*). As a rule, *k*-mers with the same frequency were arranged according to the arrangement (frequencies) of their respective reverse complements. We then arranged complementary pairs by pairs by making the two complementary *k*-mers of each pair adjacent to each other, also from highest to lowest according to the larger frequency value of each pair to obtain arrangement *A*_2_(*k*) (see also Afreixo et al., 2013).

The difference between *A*_1_(*k*) and *A*_2_(*k*) is a measure of the distance from perfect strand symmetry. In Afreixo et al. (2013), it was calculated with Ulam's metric (an algorithm to compute the distance between two permutations), which is the minimum number of moves required to go from *A*_1_(*k*) to *A*_2_(*k*). We denoted the metric as word symmetry distance 1 (*WSD*1), and its value divided by the maximum Ulam distance (the maximum possible of Ulam's metric) as normalized word symmetry distance 1 (*nWSD*1, equivalent to *Ws* in Afreixo et al., 2013) for comparison in our study. As for the novel word symmetry distance analysis, the difference between *A*_1_(*k*) and *A*_2_(*k*) was evaluated by the minimum number of moves as well as the step of each move (the number of words separating the two words of a complementary pair in the original or intermediary arrangement, see below). The step of each move was counted to amount to a distance summed up with all moves from *A*_1_(*k*) to *A*_2_(*k*). We denoted such a distance as word symmetry distance 2 (*WSD*2). Its value divided by the maximum possible of *WSD*2 for *k*-mers (see below) gave rise to normalized word symmetry distance 2 (*nWSD*2).

Take the arrangement of dinucleotide frequencies, [AG CT AT GC AA CA TG GT CG AC TC CC GA TT GG TA], as an example of *A*_1_(2). With *A*_1_(2) determined, *A*_2_(2) should be [AG CT AT GC AA TT CA TG GT AC CG TC GA CC GG TA]. From *A*_1_(2) to *A*_2_(2), *WSD*1 has a value of 3 (3 moves). For *WSD*2, TT moves to the position after AA with a step of 8, giving rise to an intermediary arrangement [AG CT AT GC AA TT CA TG GT CG AC TC CC GA GG TA]; AC moves to the position after GT with a step of 1; GA moves to the position after TC with a step of 1. As a result, *WSD*2 has a value of 10.

The maximum Ulam distance or the maximum possible of *WSD*2 for dinucleotides may be obtained from an arrangement like [AA AC AG CA CC GA AT CG GC TA TC GG TG CT GT TT]. From that, the maximum Ulam distance is 6; the maximum possible of *WSD*2 is 54 {i.e., 14 + 12 + 10 + 8 + 6 + 4, or [(14 + 4) × 6]/2}. Accordingly, *nWSD*1 is 0.500, and *nWSD*2 is 0.185 for the above example. For *k*-mers the maximum Ulam distance may be calculated as (4^*k*^−2)/2 when *k* is odd [all the complementary pairs except the last one left in the rearrangement must move to go from *A*_1_(*k*) to *A*_2_(*k*)], and as (4^*k*^−4^k/2^)/2 when *k* is even [all the complementary pairs, not including the *k*-mers that are identical to their respective reverse complements (4^k/2^ in all), must move to go from *A*_1_(*k*) to *A*_2_(*k*)]. As for the maximum possible of *WSD*2 for *k*-mers, it may be calculated as 4^*k*^ (4^*k*^−2)/4 when *k* is odd, and as (4^*k*^ + 4^k/2^-2) (4^*k*^−4^k/2^)/4 when *k* is even (formulas deduced from that for the sum of a sequence of number).

We calculated *WSD*1, *nWSD*1, *WSD*2, and *nWSD*2 at orders 1 to 10 for the complete genomes and their corresponding random sequences.

### Strand Symmetry Assessment and Statistical Analysis

We first performed an analysis in terms of the two symmetry indexes of the genomic sequences and their corresponding random sequences to have a global view of the levels of symmetry at orders 1–10. We then employed the word symmetry distance analysis to evaluate the significance of strand symmetry at a certain order for archaeal genomes and bacterial genomes, respectively, calculating at the same time *WSD*1 (*nWSD*1) and *WSD*2 (*nWSD*2) to determine the extent of strand symmetry. To further investigate higher-order strand symmetry in various situations, the archaeal and bacterial genomes, respectively, were classified and grouped according to their GC content, mononucleotide symmetry levels, or phyla/classes. In the first two grouping approaches we took into account the sample size (the number of genomes) to avoid having too few or too many genomes in a group compared with others. In the grouping by phylum/class, we considered in principle each phylum with more than 30 genomes available separately. Some related phyla, especially those with a limited number of genomes available, were put together if possible for analysis as the phylum group in systematics. And if there were more than 1,000 genomes available in a phylum, we considered instead each class in it that was with more than 30 genomes separately. After grouping we applied the word symmetry distance analysis to each of the groups. To analyze strand symmetry of individual genomes, we selected some representative ones by their mononucleotide symmetry levels as well as GC content and employed the word symmetry distance analysis with *WSD*2 only.

In all the symmetry index and word symmetry distance analysis, a comparison between the genomic sequences and their corresponding random sequences were performed with statistical test and graphic illustration. As group, each genome was with one random sequence as control; for individual genomes, each was compared with 30 corresponding random sequences.

As the values of symmetry indexes or those of word symmetry distance for a group of genomes and their corresponding random sequences were always paired (each genomic sequence vs. its corresponding random sequence) and the paired differences assumed a non-normal distribution, we employed the Wilcoxon signed-rank test for the significance of group difference between genomic sequences and random sequences. On the other hand, we employed the *t*-test for the significance of difference between the value of *WSD*2 for an individual genome and those for 30 random sequences corresponding to the genome, because the data from the random sequences took on a normal distribution. As long as the values of word symmetry distance at order *k* for a group of genomes are significantly smaller than those for the corresponding random sequences, we conclude that strand symmetry would tend to persist for *k*-mers in the genomes. The same principle applies to analysis of individual genomes.

All the calculations and statistical tests mentioned above were performed with computer programs written in Python (see Sanner, 1999).

## Results

In this section, we first present the results for the analysis of symmetry indexes. Symmetry indexes *S*_1_ and *S*_2_ may be used together to measure the levels of strand symmetry across order of oligonucleotides in genomic sequences, but they could not be employed to determine its extent. The rest of the section is all about word symmetry distance analysis. The novel *WSD*2 was compared with *WSD*1. The results show that only *WSD*2 analysis could provide a reasonable and clear-cut criterion to determine the extent of strand symmetry. We further describe the results of *WSD*2 for genomes grouped according to their GC content, mononucleotide symmetry levels, or phyla/classes. Finally, results about the extent of strand symmetry for some representative archaeal and bacterial genomes are shown in more detail. All the results indicate that there are considerable variations of higher-order strand symmetry among genomes. For further assessment and explanations of the results, they can be found in the Discussion section.

### Symmetry Indexes of the Complete Genomes and Their Corresponding Random Sequences

**Figure 1:**
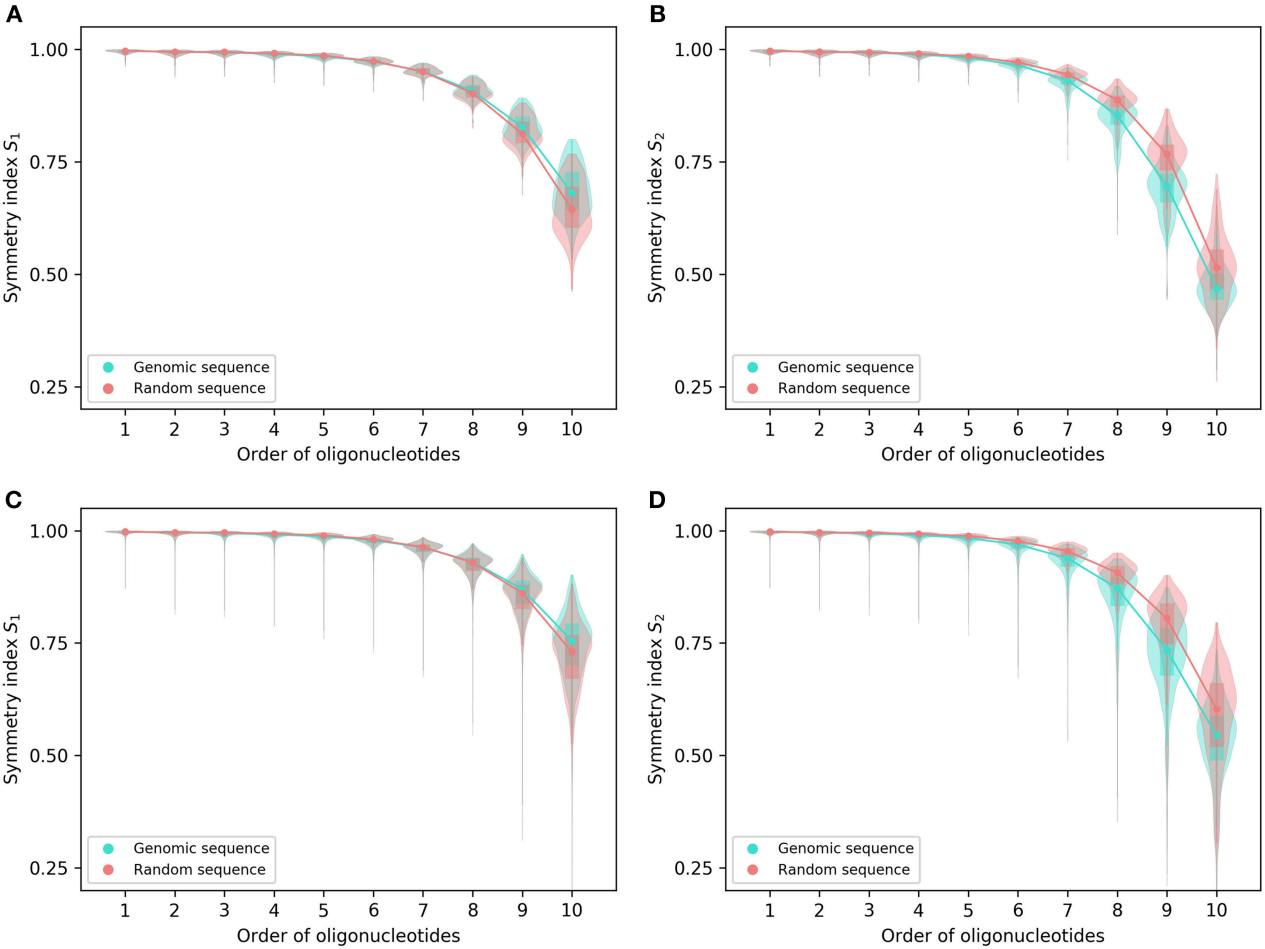
Symmetry indexes of the complete genomes and their corresponding random sequences. **(A)** *S*_1_ and **(B)** *S*_2_ of 206 archaeal genomes and their corresponding random sequences. **(C)** *S*_1_ and **(D)** *S*_2_ of 2,659 bacterial genomes and their corresponding random sequences. The rectangles in the violin plot indicate the interquartile range of the data at different orders for genomic sequences and random sequences, respectively. Medians are marked with dots.

Both symmetry indexes (*S*_1_ and *S*_2_) may provide an overall view of the levels of strand symmetry across orders of oligonucleotides for the complete genomes in spite of considerable variations among individual genomes in symmetry index values (Figure 1). The values of the two symmetry indexes are usually close to 1 for lower-order oligonucleotides. They decrease generally and gradually with the increase of orders, and the trend becomes more pronounced for higher-order oligonucleotides. However, there is no abrupt change of the values across orders.

For both *S*_1_ and *S*_2_, the archaeal genomes have values identical to or not significantly different from those of their corresponding random sequences for 1- and 2-mers, whereas the bacterial genomes tend to be smaller than the random sequences in both values for 2-mers (Figure 1; for details and *P*-values see Supplementary Material 2). For other *k*-mers there are usually differences between genomic sequences and random sequences in *S*_1_ and *S*_2_ values except *S*_1_ for 7-mers of the archaeal genomes. For *S*_1_, the values of genomic sequences tend to be smaller than those of random sequences for 3-mers through 7-mers (bacterial genomes only for 7-mers), and tend to be larger than those of random sequences for 8-mers through 10-mers (Figures 1A,C). As for *S*_2_, the values of genomic sequences tend to be smaller than those of random sequences for 3-mers through 10-mers (Figures 1B,D).

In general, the values of *S*_1_ are somewhat larger than those of *S*_2_ at the same order for the complete genomes as well as the corresponding random sequences, especially for higher-order *k*-mers (Figures 1A–D).

From the pattern across orders and the difference of genomic sequences from random sequences, it appears *S*_1_ and *S*_2_, even taken together, could not provide reasonable and reliable criteria to determine the extent of strand symmetry for a sequence.

### Evaluation of Strand Symmetry in Groups of Genomes by Word Symmetry Distance Analysis

#### Strand Symmetry in All the Archaeal Genomes and All the Bacterial Genomes Studied

**Figure 2:**
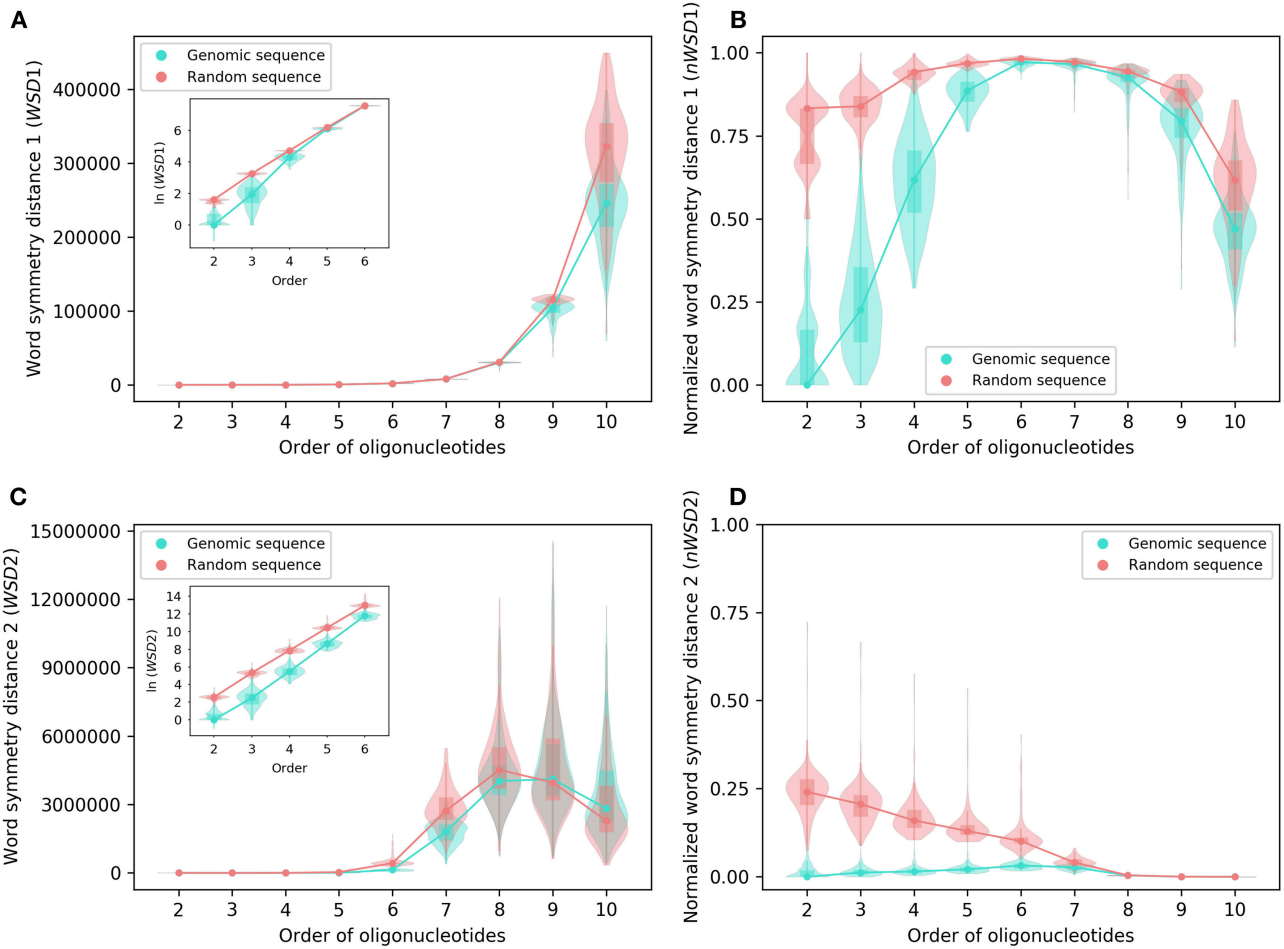
Various word symmetry distances for 206 archaeal genomes and their corresponding random sequences. **(A)** *WSD*1. **(B)** *nWSD*1. **(C)** *WSD*2. **(D)** *nWSD*2. The values of *WSD* are presented in natural logarithm in insets **(A,C)**. For other explanations see the legend of Figure 1.

**Figure 3:**
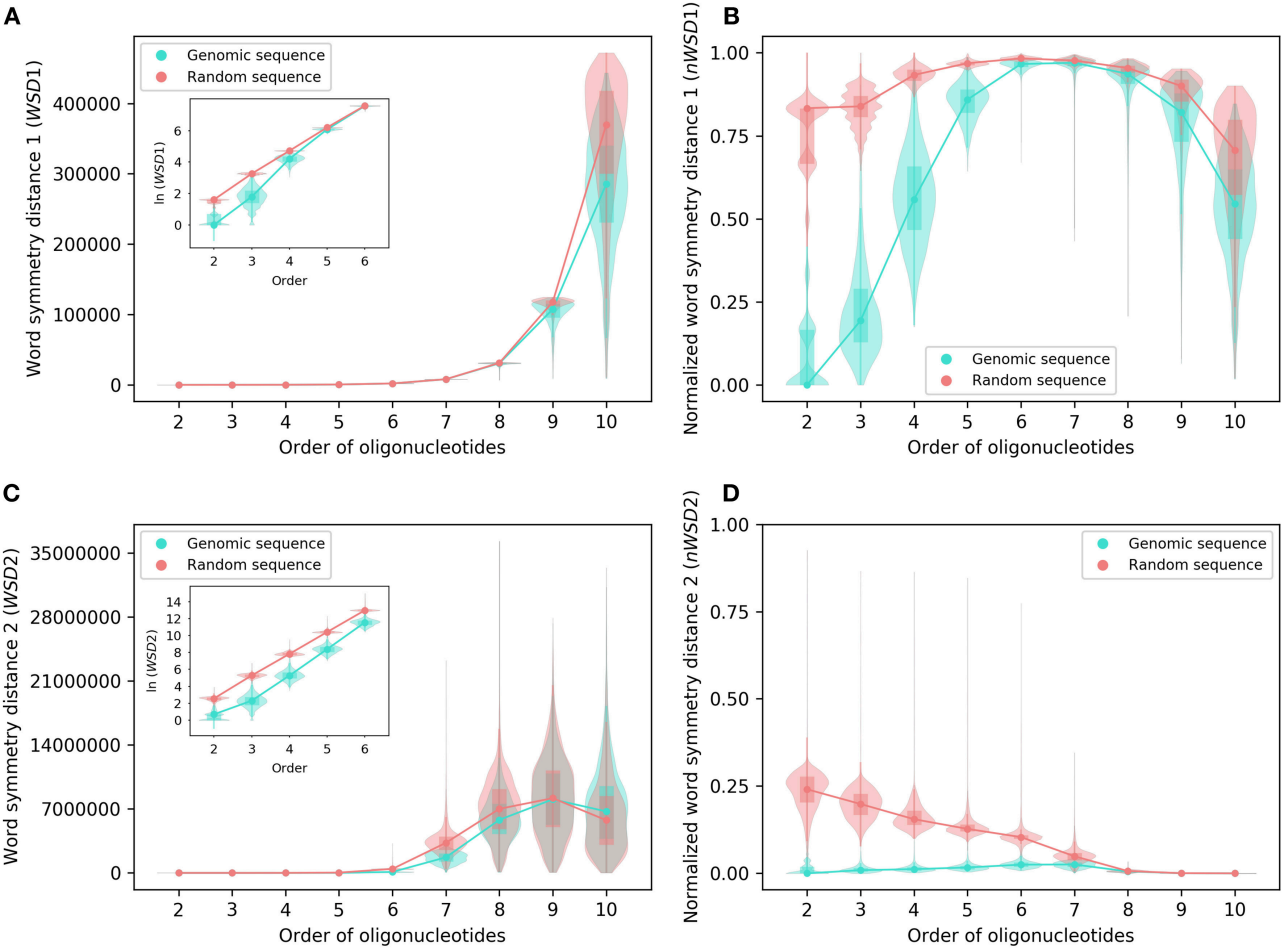
Various word symmetry distances for 2,659 bacterial genomes and their corresponding random sequences. **(A)** *WSD*1. **(B)** *nWSD*1. **(C)** *WSD*2. **(D)** *nWSD*2. For other explanations see the legend of Figures 1,2.

Analyzed as group, the results of *WSD*1, *nWSD*1, *WSD*2, and *nWSD*2 from 2-mers through 10-mers for the 206 archaeal genomes are shown in Figure 2, those for the 2,659 bacterial genomes in Figure 3. They are presented along with the results for the corresponding random sequences [for 1-mers, the values for all the genomes studied and all the random sequences are always the same (zero), thus not included in the results].

For all sequences, the values of *WSD*1 increase approximately exponentially with orders of oligonucleotides up to 10-mers (Figures 2A, 3A), while the values of *nWSD*1 increase to a maximum at order 6 or 7 and then decrease (Figures 2B, 3B). The values of *nWSD*1 for the genomic sequences are obviously smaller than those for the random sequences at orders 2 to 5. On the other hand, the values of *WSD*2 for all sequences increase approximately exponentially with orders to 7-mers only and then level off at 9-mers, and finally decrease at 10-mers (Figures 2C, 3C). For *nWSD*2, the values for the genomic sequences change little (with a maximum median at order 6 or 7 nevertheless) while those for the random sequences decrease gradually with orders and converge seemingly with the values for the genomic sequences toward zero at 9- and 10-mers (Figures 2D, 3D).

Comparing the data of genomic sequences with those of random sequences, it is clear from *nWSD*1 and *nWSD*2 that the archaeal genomes and the bacterial genomes usually have smaller values than the random sequences for up to 5-mers (*nWSD*1) or 6-mers (*nWSD*2) (Figures 2B,D, 3B,D; for details and *P*-values see Supplementary Material 3). And there is no doubt that strand symmetry would persist generally for up to at least 5-mers (*nWSD*1) or 6-mers (*nWSD*2) in the genomes studied. To determine the extent of strand symmetry, the results of word symmetry distance analysis for higher *k*-mers would be crucial.

For *WSD*1 (*nWSD*1), the genomic sequences have similar values as the random sequences for 6- and 7-mers, nonetheless they are significantly different [the genomic sequences also tend to be smaller in *WSD*1 (*nWSD*1) values, *P* < 0.0001]; the genomic sequences continue tending to have smaller values than the random sequences for 8-mers through 10-mers (Figures 2A,B, 3A,B). Results of *WSD*1 analysis for smaller and more appropriate sample sizes remain practically the same (see the following sections of Evaluation of Strand Symmetry in Groups of Genomes by Word Symmetry Distance Analysis). Therefore, in addition to being inadequate in design, *WSD*1 (*nWSD*1) could not offer a clear-cut measure to determine the order of oligonucleotides at which strand symmetry no longer persists in the archaeal and bacterial genomes.

In contrast, the results of *WSD*2 and *nWSD*2 may provide such a clear-cut measure (Figures 2C,D, 3C,D). The values of *WSD*2 (*nWSD*2) for the archaeal and bacterial genomes, respectively, tend to be smaller than those for the corresponding random sequences for 7- and 8-mers (*P* < 0.0001). The values for the bacterial genomes also tend to be smaller than those for the corresponding random sequences for 9-mers (*P* < 0.0001). Conversely, the values for the archaeal genomes tend to be larger than those for the random sequences for 9- and 10-mers, so do the values for the bacterial genomes for 10-mers (*P* < 0.0001). The result for 9- and 10-mers of the archaeal genomes and that for 10-mers of the bacterial genomes may provide just what is needed to determine the order of oligonucleotides at which strand symmetry no longer persists. Therefore, it may be concluded from the statistical results of *WSD*2 and *nWSD*2 that strand symmetry would tend to persist for up to 8-mers in the group of archaeal genomes, and up to 9-mers in the group of bacterial genomes. Strand symmetry would likely break down from 9-mers in the archaeal genomes, and from 10-meres in the bacterial genomes. At order 8, the archaeal genomes have a median of *S*_1_ a little over 0.90, and a median of *S*_2_ a little over 0.85, while the corresponding values of the bacterial genomes are a little over 0.93 and 0.87, respectively. At order 9, the medians of *S*_1_ and *S*_2_ of the bacterial genomes are still over 0.86 and 0.73, respectively, considerably larger than the corresponding value of the archaeal genomes (Figure 1 and Supplementary Material 2). Overall, the bacterial genomes would have higher strand symmetry levels than the archaeal genomes for 8- and 9-mers. On the other hand, as there are considerable variations among individual genomes in terms of symmetry index values and word symmetry distance values in such large groups as archaea or bacteria (Figures 1–3), the above results on the extent of strand symmetry would just provide a sketchy account of the genomes studied. More detailed and precise results may be obtained in word symmetry distance analysis with smaller groups or individual genomes.

#### Strand Symmetry in Groups of Genomes Classified According to Their GC Content

Among the genomes studied, the GC content varies from 24.24% to 68.41% in the archaea, and from 13.54% to 74.84% in the bacteria. We classified the 206 archaeal genomes into 4 groups, and the 2,659 bacterial genomes into 10 groups, according to their genomic GC content. The order of oligonucleotides up to which strand symmetry would tend to persist in each group of genomes was determined by the same analysis of word symmetry distance as employed in section Strand Symmetry in All the Archaeal Genomes and All the Bacterial Genomes Studied. The results of the analysis for each group are shown in Table 1 (*WSD*1 and *nWSD*1 could not provide a clear-cut measure either in this analysis of groups of genomes, their results are not included in the table; for details and *P*-values see Supplementary Material 4).

**Table 1:** Extent of strand symmetry determined by word symmetry distance analysis (*WSD*2 and *nWSD*2) in groups of archaeal and bacterial genomes classified according to their GC content.

**Group**	**GC content (%)**	**Member**	**Order**	***P*-value**
A1 (GC)	[24.24, 40.00)	56	8	<0.0001
A2 (GC)	[40.00, 50.00)	64	8	<0.0001
A3 (GC)	[50.00, 60.00)	47	8	<0.0001
A4 (GC)	[60.00, 68.41]	39	9	<0.0001
B1 (GC)	[13.538, 30.00)	174	8	<0.0001
B2 (GC)	[30.00, 35.00)	210	8	<0.0001
B3 (GC)	[35.00, 40.00)	348	8	<0.0001
B4 (GC)	[40.00, 45.00)	316	8	<0.0001
B5 (GC)	[45.00, 50.00)	212	8	<0.0001
B6 (GC)	[50.00, 55.00)	215	8	<0.0001
B7 (GC)	[55.00, 60.00)	277	9	<0.0001
B8 (GC)	[60.00, 65.00)	369	9	<0.0001
B9 (GC)	[65.00, 70.00)	369	9	<0.0001
B10 (GC)	[70.00, 74.841]	169	9	<0.0001

*A1 (GC)–A4 (GC) are groups of archaeal genomes by GC content; B1 (GC)–B10 (GC) are groups of bacterial genomes by GC content; member is the number of genomes; order is the order of oligonucleotides up to which strand symmetry would tend to persist in the group of genomes concerned; P-value is the P-value for the two-tailed Wilcoxon signed-rank test*.

For the archaeal genomes, strand symmetry would tend to persist for up to 8-mers in the low- and intermediate-GC content groups, and up to 9-mers in the high-GC content group. The same is true of the bacterial genomes with more than one high-GC content group. These results are consistent with the fact that high-GC content genomes tend to have higher levels of strand symmetry for mononucleotides and oligonucleotides compared with other genomes. Actually, there is a positive correlation of levels of strand symmetry of complete genomes with genomic GC content, a correlation comparable to that of strand symmetry with genome sizes (Supplementary Material 5). As the proportion of high-GC content genomes is larger in the bacterial genomes studied than in the archaeal genomes studied, strand symmetry may tend to persist for *k*-mers higher in the bacterial genomes than in the archaeal genomes (9-mers vs. 8-mers, see also section Strand Symmetry in All the Archaeal Genomes and All the Bacterial Genomes Studied).

#### Strand Symmetry in Groups of Genomes Classified According to Their Mononucleotide Symmetry Levels

As the values of *S*_1_ and *S*_2_ are very similar for mononucleotides of a complete genome, and *S*_1_ is more widely used, we classified and grouped the archaeal and bacterial genomes according to their *S*_1_ values for mononucleotides. *S*_1_ for mononucleotides ranges from 0.9627 to 0.9998 for the 206 archaeal genomes, and from 0.8721 to 0.9999 for the 2,659 bacterial genomes (see also Supplementary Material 2). We classified the archaeal genomes into 4 groups, and the bacterial genomes into 11 groups. The results of word symmetry distance analysis (*WSD*2 and *nWSD*2) are shown in Table 2 (for details, including *WSD*1 and *nWSD*1, and *P*-values see Supplementary Material 6).

**Table 2:** Extent of strand symmetry determined by word symmetry distance analysis (*WSD*2 and *nWSD*2) in groups of archaeal and bacterial genomes classified according to their mononucleotide symmetry levels (*S*_1_).

**Group**	***S*_**1**_**	**Member**	**Order**	***P*-value**
A1 (*S*_1_)	[0.9627, 0.9940)	50	8	<0.0001
A2 (*S*_1_)	[0.9940, 0.9965)	47	8	<0.0001
A3 (*S*_1_)	[0.9965, 0.9985)	67	8	<0.0001
A4 (*S*_1_)	[0.9985, 0.99984]	42	9	<0.05
B1 (*S*_1_)	[0.87209, 0.9900)	200	7	<0.0001
B2 (*S*_1_)	[0.9900, 0.9940)	252	8	<0.0001
B3 (*S*_1_)	[0.9940, 0.9950)	123	8	<0.0001
B4 (*S*_1_)	[0.9950, 0.9960)	193	8	<0.0001
B5 (*S*_1_)	[0.9960, 0.9970)	297	8	<0.0001
B6 (*S*_1_)	[0.9970, 0.9975)	218	8	<0.0001
B7 (*S*_1_)	[0.9975, 0.9980)	267	9	<0.0001
B8 (*S*_1_)	[0.9980, 0.9985)	323	9	<0.0001
B9 (*S*_1_)	[0.9985, 0.9990)	358	9	<0.0001
B10 (*S*_1_)	[0.9990, 0.9995]	297	9	<0.0001
B11 (*S*_1_)	[0.9995, 0.99994]	131	9	<0.0001

*A1 (S_1_)–A4 (S_1_) are groups of archaeal genomes by S_1_; B1 (S_1_)–B11 (S_1_) are groups of bacterial genomes by S_1_; member is the number of genomes; order is the order of oligonucleotides up to which strand symmetry would tend to persist in the group of genomes concerned; P-value is the P-value for the two-tailed Wilcoxon signed-rank test*.

It is natural that genomes with high values of *S*_1_ for mononucleotides are likely to have better higher-order strand symmetry. This is what is shown in Table 2 for the groups of genomes classified according to their mononucleotide symmetry levels.

For the archaeal genomes, strand symmetry would tend to persist for up to 8-mers in groups of genomes with mononucleotide *S*_1_ < 0.9985, and up to 9-mers in the group with larger *S*_1_ values (group A4 in Table 2). The result for group A4 is only marginally significant [for the assumption that the *WSD*2 (*nWSD*2) values for 9-mers for the genomes would tend to be smaller than those for their corresponding random sequences, 0.04 < *P* < 0.05]. In this group of 42 genomes, actually only a little more than half are with *WSD*2 (*nWSD*2) values for 9-mers smaller than those for their corresponding random sequences.

The results for the bacterial genomes are more revealing. There are quite a few bacterial genomes whose mononucleotide *S*_1_ values are relatively low (including several ones with exceptionally low values). Consequently, strand symmetry in the group with the lowest mononucleotide symmetry levels (group B1 in Table 2) would tend to persist for up to 7-mers only. In fact, strand symmetry in some individual genomes of this group may even be less satisfactory, persisting for up to 6-mers only (see section Evaluation of Strand Symmetry in Individual Genomes by Word Symmetry Distance Analysis). For the groups of genomes with relatively high (intermediate) values of mononucleotide *S*_1_, strand symmetry would tend to persist for up to 8-mers (groups B2 to B6 in Table 2). And for the groups of genomes with high values of mononucleotide *S*_1_, strand symmetry would tend to persist for up to 9-mers (groups B**7** to B11 in Table 2). Such results are comparable and coherent with those of groups of genomes classified by GC content. Mononucleotide symmetry levels as well as GC content of genomes seem to be important factors relevant to their higher-order strand symmetry.

#### Strand Symmetry in Groups of Genomes Classified According to Their Phyla/Classes

To figure out whether strand symmetry, especially that for higher-order oligonucleotides, is taxon-specific, we classified the genomes studied according to the phyla or classes of the species concerned. Most of the 206 archaeal genomes belong to either phylum Crenarchaeota or phylum Euryarchaeota. And there were no sufficient genomes of other archaeal phyla for the analysis. On the other hand, plentiful bacterial genomes of various phylum groups (each consisting of several related phyla), phyla, or classes were available. In total, 14 groups by phylum/class were used for word symmetry distance analysis. The results (*WSD*2 and *nWSD*2) are shown in Table 3 (for details, including *WSD*1 and *nWSD*1, and *P*-values see Supplementary Material 7).

**Table 3:** Extent of strand symmetry determined by word symmetry distance analysis (*WSD*2 and *nWSD*2) in groups of archaeal and bacterial genomes classified according to their phyla/classes.

**Group**	**Phylum group/phylum/class**	**Member**	**Order**	***P*-value**
A1 (phylum)	Crenarchaeota	43	8	<0.0001
A2 (phylum)	Euryarchaeota	145	8	<0.0001
B1 (phylum group)	FCB group	200	8	<0.0001
B2 (phylum group)	PVC group	43	8	<0.0001
B3 (phylum)	Spirochaetes	45	8	<0.0001
B4 (phylum)	Actinobacteria	382	9	<0.0001
B5 (phylum)	Cyanobacteria	82	8	<0.0001
B6 (phylum)	Firmicutes	467	8	<0.0001
B7 (phylum)	Tenericutes	70	8	<0.01
B8 (class)	Alphaproteobacteria	338	9	<0.0001
B9 (class)	Betaproteobacteria	219	9	<0.0001
B10 (class)	Deltaproteobacteria	71	9	<0.005
B11 (class)	Epsilonproteobacteria	52	8	<0.0001
B12 (class)	Gammaproteobacteria	556	9	<0.0001

*A1–A2 are groups of archaeal genomes; B1–B12 are groups of bacterial genomes; FCB group (B1) includes phyla Bacteroidetes, Candidatus Cloacimonetes, Chlorobi, Fibrobacteres, Gemmatimonadetes, and Ignavibacteriae in the present study; PVC group (B2) includes phyla Chlamydiae, Kiritimatiellaeota, Planctomycetes, and Verrucomicrobia in the present study; Alpha-, Beta-, Delta-, Epsilon-, and Gamma-proteobacteria (B8–B12) are classes of the phylum Proteobacteria; member is the number of genomes; order is the order of oligonucleotides up to which strand symmetry would tend to persist in the group of genomes concerned; P-value is the P-value for the two-tailed Wilcoxon signed-rank test*.

Although strand symmetry would tend to persist for up to 8-mers for both phyla of the archaeal genomes (groups A1 and A2 in Table 3) and therefore seems not to be taxon-specific, for the bacterial genomes there may be differences of higher-order symmetry levels among groups of different phyla/classes. Usually the difference is about strand symmetry up to 8-mers vs. 9-mers. Strand symmetry in the phylum Actinobacteria (group B4 in Table 3) and four classes of the phylum Proteobacteria (Alpha-, Beta-, Delta-, and Gamma-proteobacteria; groups B8–B10, B12 in Table 3) would all tend to persist for up to 9-mers. In all the other groups, including Epsilonproteobacteria, strand symmetry would tend to persist for up to 8-mers.

The patterns of higher-order strand symmetry of genomes in groups of different phyla/classes seem to correlate with the distributions of GC content and mononucleotide *S*_1_ values of genomes in those groups, rather than specifically with phyla/classes themselves.

For the archaeal genomes, the distribution of genomic GC content and that of mononucleotide *S*_1_ values are not considerably different between the two phyla studied. In both phyla there are low-, intermediate-, and high-GC content genomes with comparable proportions; mononucleotide *S*_1_ values are usually larger than 0.990, and the proportions of genomes with the highest ones (≥0.999) are relatively small (for details see Supplementary Material 8). Combining with the results of the previous sections of Evaluation of Strand Symmetry in Groups of Genomes by Word Symmetry Distance Analysis, it seems not unusual that higher-order symmetry levels would be the same (persisting for up to 8-mers) in both phyla.

The situations for the bacterial genomes are not the same. There may be considerable differences for the distributions of genomic GC content and mononucleotide *S*_1_ values in different groups. As a principle, groups in which genomes are mostly with high-GC content or intermediate-to-high-GC content would be with strand symmetry up to 9-mers (groups B4, B8–B10, B12 in Table 3; for details see Supplementary Material 8). In these groups the proportions of genomes with the highest mononucleotide *S*_1_ values (≥0.998) are relatively large. The other groups, without large enough proportions of high-GC content genomes, would be with strand symmetry up to 8-mers. In them the proportions of genomes with the highest mononucleotide *S*_1_ values are relatively small (with the exception of PVC group and Cyanobacteria, Supplementary Material 8). It is clear that the above criteria are also true of the classes of the phylum Proteobacteria in which Epsilonproteobacteria stands alone in terms of higher-order strand symmetry of its genomes (up to 8-mers vs. up to 9-mers in other classes).

The fact that classes of the same phylum may have different higher-order symmetry levels for their genomes if they have different distributions of genomic GC content and mononucleotide *S*_1_ values would underscore the assumption that phyla/classes themselves would not be the primary factor relevant to higher-order strand symmetry.

### Evaluation of Strand Symmetry in Individual Genomes by Word Symmetry Distance Analysis

The above results of word symmetry distance analysis of groups of genomes deal with the level of strand symmetry of the majority of genomes in each group. As there may be differences among genomes in all cases, word symmetry distance analysis of individual genomes must be performed to have an unequivocal conclusion for a given genome.

We selected some representative genomes with different mononucleotide symmetry levels and GC content from the archaeal and bacterial genomes studied for the purpose. The results of word symmetry distance analysis (*WSD*2) of the individual genomes are shown in Table 4 (for details and *P*-values see Supplementary Material 9).

**Table 4:** Extent of strand symmetry determined by word symmetry distance analysis (*WSD*2) in individual genomes with different mononucleotide symmetry levels (*S*_1_) and GC content.

**Genome (species)**	***S*_**1**_**	**GC content (%)**	**Order**	***P*-value**
*Nanohaloarchaea archaeon* SG9 (A)	0.9627	46.37	7	<0.0001
*Methanococcus voltae* A3 (A)	0.9934	28.59	7	<0.0001
*Methanoculleus bourgensis* MS2 (A)	0.9941	60.64	8	<0.0005
*Methanosarcina* sp. WH1 (A)	0.9985	41.82	8	<0.0001
*Natrialba magadii* ATCC 43099 (A)	0.9997	61.42	9	<0.0001
*Candidatus* Nitrocosmicus oleophilus (A)	0.9998	34.14	7	<0.0001
*Candidatus* Hodgkinia cicadicola (B)	0.8721	46.39	6	<0.0001
*Filifactor alocis* ATCC 35896 (B)	0.9182	35.45	7	<0.0001
*Idiomarina loihiensis* L2TR (B)	0.9856	47.04	7	<0.0001
*Pseudoalteromonas phenolica* (B)	0.9934	40.58	8	<0.0001
*Lactobacillus amylophilus* DSM 20533 = JCM 1125 (B)	0.9948	43.65	7	<0.0001
*Pseudoalteromonas* sp. SM9913 (B)	0.9954	40.28	9	<0.0001
*Flammeovirga* sp. MY04 (B)	0.9963	34.57	8	<0.0001
*Planococcus maritimus* (B)	0.9973	47.17	8	<0.0001
*Exiguobacterium* sp. ZWU0009 (B)	0.9976	47.06	8	<0.0001
*Chlorobaculum parvum* NCIB 8327 (B)	0.9984	55.80	8	<0.0001
*Mycobacterium* sp. JS623 (B)	0.9988	65.07	9	<0.0001
*Maricaulis maris* MCS10 (B)	0.9994	62.73	9	<0.0001
*Sphingomonas* sp. LK11 (B)	0.9997	66.16	9	<0.0001
*Actinobacillus suis* ATCC 33415 (B)	0.9999	40.22	8	<0.0001

*Archaeal genomes and bacterial genomes, respectively, are arranged by their S_1_ values; (A) denotes an archaeon; (B) denotes a bacterium; order is the order of oligonucleotides up to which strand symmetry would tend to persist in the genome concerned; P-value is the P-value for the one-sample two-tailed t-test*.

**Figure 4:**
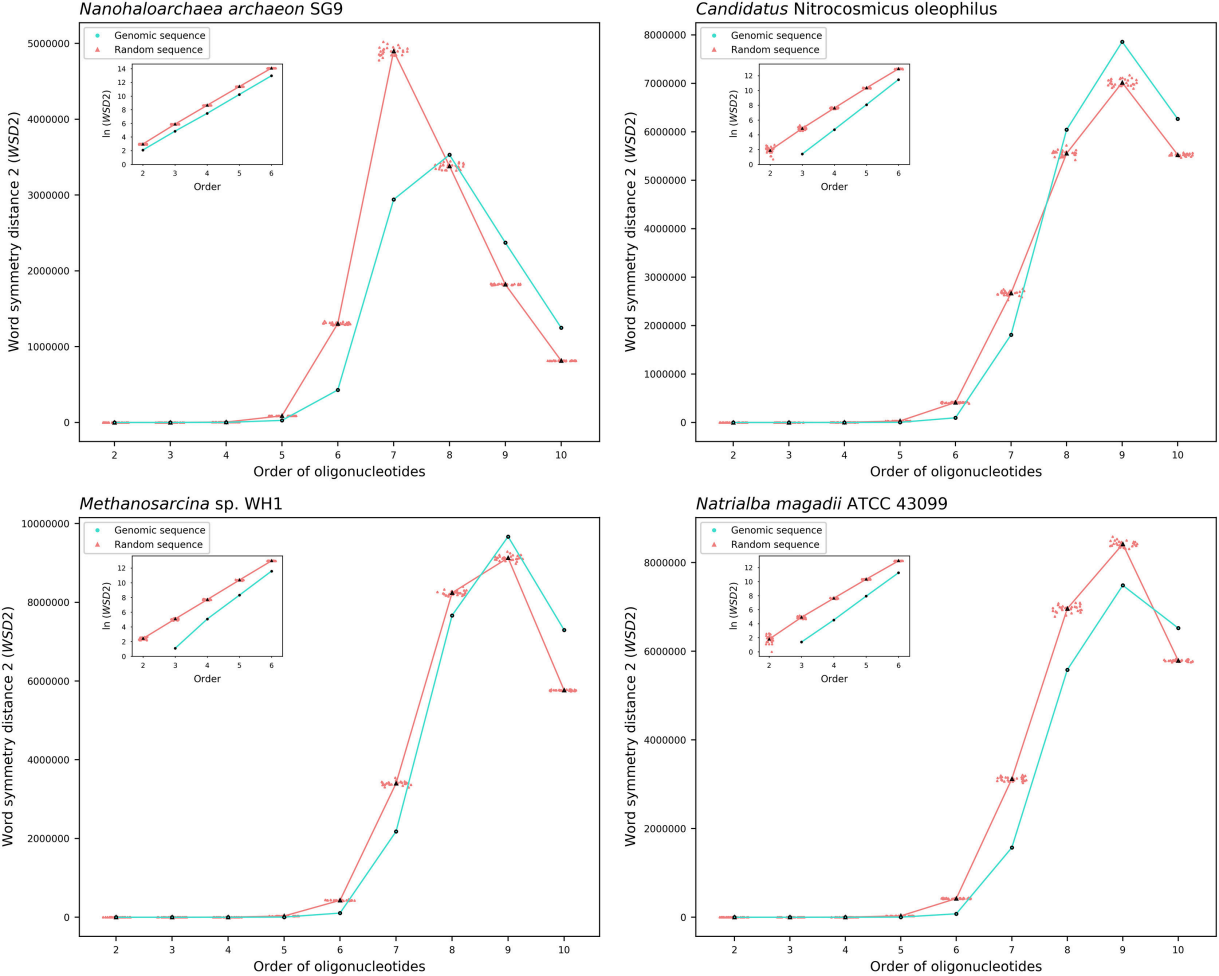
Word symmetry distances (*WSD*2) for four representative archaeal genomes (*Nanohaloarchaea archaeon* SG9, *Candidatus* Nitrocosmicus oleophilus, *Methanosarcina* sp. WH1, *Natrialba magadii* ATCC 43099) and their corresponding random sequences. *WSD*2 value for each genome and the mean value for its corresponding random sequences, respectively, are marked for each order. Data dots for the random sequences are horizontally dispersed within the range of an order for better visualization. The values of *WSD*2 are presented in natural logarithm in insets (the logarithmic value for genomic sequences may not be available at order 2 where *WSD*2 is zero).

**Figure 5:**
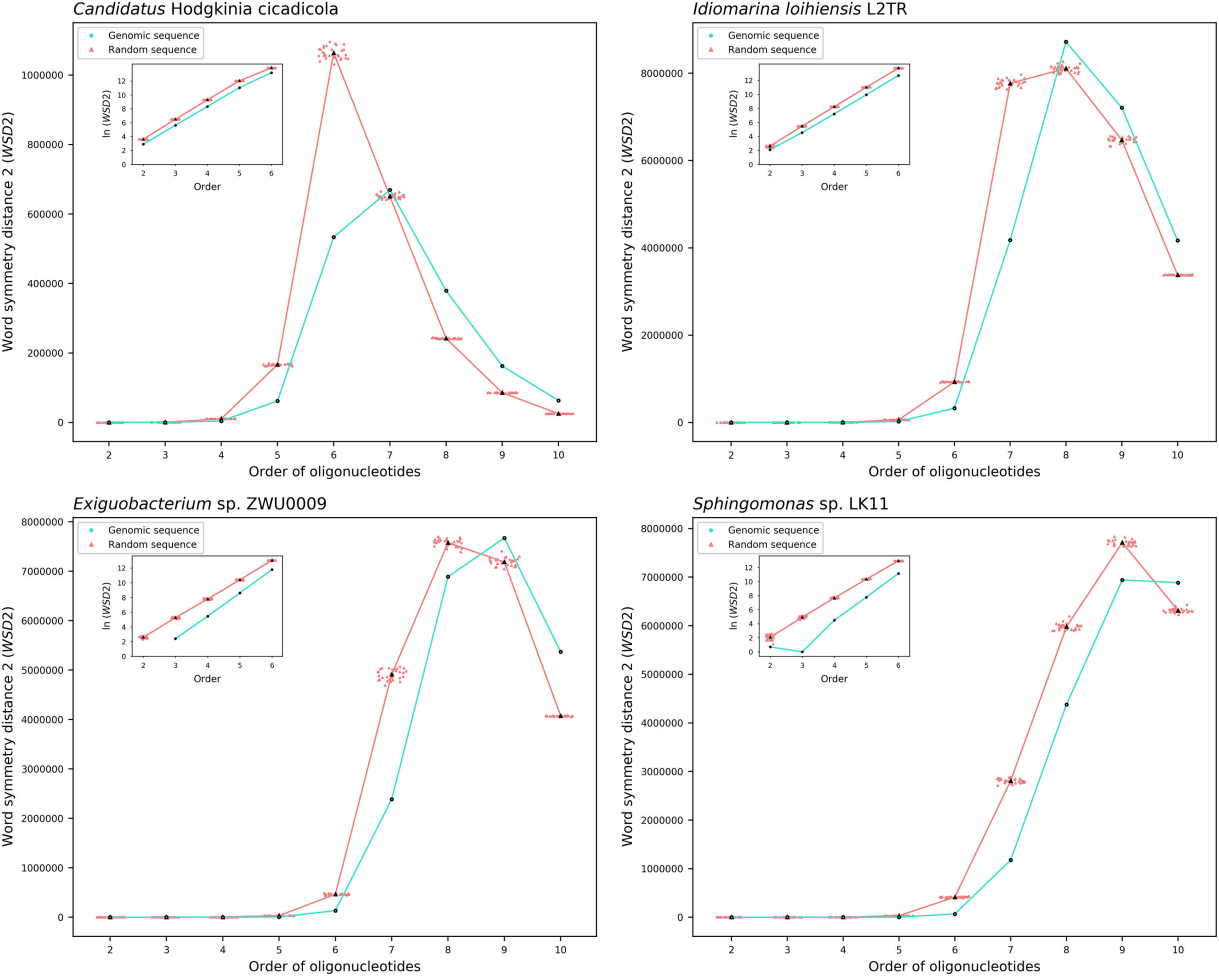
Word symmetry distances (*WSD*2) for four representative bacterial genomes (*Candidatus* Hodgkinia cicadicola, *Idiomarina loihiensis* L2TR, *Exiguobacterium* sp. ZWU0009, *Sphingomonas* sp. LK11) and their corresponding random sequences. For other explanations see the legend of Figure 4.

The results about the extent of strand symmetry of the individual genomes are apparent in graphic illustrations (Figures 4, 5, and Supplementary Material 9). For individual genomes, the extent of strand symmetry may be up to as low as 6-mers, or as high as 9-mers. In many cases of the archaeal and bacterial genomes, those with relatively low mononucleotide symmetry levels and GC content would tend to have strand symmetry persisting for up to 7-mers (for certain bacterial genomes up to 6-mers only); those with relatively high mononucleotide symmetry levels and GC content would tend to have strand symmetry persisting for up to 8- or 9-mers (there may be exceptions, e.g., *Candidatus* Nitrocosmicus oleophilus, see Table 4).

Therefore, there would be considerable variations among individual genomes for the extent of strand symmetry. And individually, it would also be a feature related, though there may be exceptions, to mononucleotide symmetry levels and GC content of the genomes concerned.

## Discussion

To determine the extent of strand symmetry in modern genomic sequences is crucial for the further understanding of the ubiquitous phenomenon. Symmetry indexes *S*_1_ and *S*_2_ used in our study measure the measure the frequency similarity/difference of complementary *k*-mers. They may provide an overall view of the levels of strand symmetry across orders of oligonucleotides for genomic sequences. However, they could not determine to what extent strand symmetry still persists because they do not take into account the frequency pattern of non-complementary *k*-mer pairs. In random sequences generated according to for mononucleotides), mononucleotides follow the same symmetry pattern as their corresponding genomic sequences. And all *k*-mers (*k* ≥ 2) of the same order with the same GC content (including the complementary pairs) in the random sequences would have practically the same probability to occur. That is fundamentally different from the higher-order symmetry pattern of genomic sequences, in which only or mainly the frequencies of words and those of their respective reverse complements are with marked similarity. The symmetry indexes could hardly distinguish the difference of frequency pattern between genomic sequences and corresponding random sequences. Word symmetry distance analysis, in contrast, exploits such a difference to determine the extent of strand symmetry in genomic sequences. Symmetry indexes *S*_1_ and *S*_2_ may be combined with word symmetry distance analysis to have a better illustration of the levels of strand symmetry.

The novel approach (*WSD*2) proposed in this study, rather than the previous word symmetry distance analysis (*WSD*1), seems to meet the need to determine the extent of strand symmetry. Although *WSD*1 (*nWSD*1) could not fulfill the requirement, it inspired us to propose *WSD*2 (*nWSD*2), which is an important amendment of it. *WSD*1 (*nWSD*1) would considerably underestimate the word symmetry distance for higher-order oligonucleotides and random sequences, therefore there would be a bias in the calculation of higher-order word symmetry distance as well as in the comparison between genomic sequences and their corresponding random sequences. On the other hand, *WSD*2 (*nWSD*2) takes into account the number of words that separates each complementary pair in the word frequency arrangement. It could reflect the adequate word symmetry distance for both higher-order oligonucleotides and random sequences.

In terms of the pattern of *WSD*1, its values would increase with orders for both genomic sequences and their corresponding random sequences, while the random sequences would require more moves to obtain the final frequency arrangement. The number of different higher-order oligonucleotides would be less numerous in genomic sequences compared to their corresponding random sequences, leading also to fewer necessary moves and smaller *WSD*1 values for the genomic sequences. Therefore, *WSD*1 (*nWSD*1) would always have larger values for the random sequences across orders. Such a difference between genomic sequences and their corresponding random sequences is statistically significant for both large and small groups of genomes, indicating that sample size would not be a critical issue of concern in the analysis.

In comparison, the values of *WSD*2 would not always increase with orders. They would level off and then decrease. For higher-order oligonucleotides, though more and more moves would be needed to achieve the final frequency arrangement as the order increases, the steps of moves would not be as big as in the previous order. Consequently, the *WSD*2 values would peak at a certain order and then decrease, as illustrated in graphs. The values for the random sequences are larger than those for genomic sequences at lower-orders, while the reverse would be true of higher-order oligonucleotides. Although the number of moves required for the random sequences would be larger than that for genomic sequences for higher-order oligonucleotides, their steps would generally be smaller due to the randomness of frequencies beginning from a certain order. The values of *WSD*2 for genomic sequences would therefore be larger than or approximately equal to those for their corresponding random sequences beginning from that order. And there would appear a crossing in the graphic illustration, making it possible to judge from which order strand symmetry would begin to break down. As a result, *WSD*2 (*nWSD*2) worked well in our study. In graphic illustration, *nWSD*2 seems to work better for the evaluation of strand symmetry for lower-order oligonucleotides, while *WSD*2 is more suitable for higher-order oligonucleotides for the same purpose.

Furthermore, genome sizes play a role in the calculation of word symmetry distances. Large genomes may provide more chances for rare higher-order oligonucleotides to occur, thus leading to more words in the frequency arrangement and larger word symmetry distances. The opposite would be true of small genomes (see also FCB group, PVC group, and phylum Spirochaestes in Supplementary Material 7).

The result of our study has also revealed that higher-order strand symmetry would tend to positively correlate with GC content and mononucleotide symmetry levels of the genomic sequences. High mononucleotide symmetry levels provide the basis for higher-order symmetry by making the occurrence frequencies of higher-order oligonucleotides with the same GC content have more chances to be similar. As the oligonucleotides of a complementary pair are of the same GC content, they would probably be closer in the frequency arrangement for sequences with high mononucleotide symmetry levels, leading to better higher-order symmetry in the distance analysis. As for the correlation of higher-order strand symmetry and genomic GC content, that would be relevant to the positive correlation of mononucleotide symmetry levels with GC content (see also section Strand Symmetry in Groups of Genomes Classified According to Their GC Content) and might be relevant to the origin of strand symmetry itself.

In the present work, we applied the novel word symmetry distance analysis to the study of archaeal and bacterial genomes. Actually, the novel word symmetry distance analysis works as well for eukaryotic genomes. Our preliminary results show that strand symmetry would persist for up to 9-mers in the human genome, consistent with our previous estimation (Zhang, 2015). It seems there are no strict structural or functional constraints for the persistence (maintenance) of higher-order strand symmetry. Symmetry levels decrease with order of oligonucleotides. Strand symmetry would break down at a certain order. That would be the main reason why it would persist for up to 9-mers at most in the genomes studied. On the other hand, further development of the method would be needed for the analysis of genomes with extreme GC content and very small sizes, such as some very small bacterial genomes, some chloroplast and mitochondrial genomes. And that is the study in our ongoing work.

The results of our present study may provide additional clues for the issue about how strand symmetry in modern genomes originated in the first place. The variations of higher-order oligonucleotide strand symmetry among genomes would be an indication that there is no universal role of strand symmetry in modern genomes. That would provide further information to judge whether strand symmetry in modern genomes would originate from early genomes without the feature, or it would date back from the most primitive genome. The former scenario considers strand symmetry as the result of selection of stem-loop structures (Forsdyke, 1995a,b), no strand biases for mutation and selection (Lobry, 1995; Lobry and Lobry, 1999), or strand inversion/inverted transposition (Fickett et al., 1992; Albrecht-Buehler, 2006), while the latter as original trait of the primordial genome (Zhang and Huang, 2008, 2010; Zhang et al., 2013).

## Conclusion

In order to determine the extent of strand symmetry in modern genomic sequences, we developed an algorithm for the novel word symmetry distance analysis (*WSD*2). The novel approach could provide a clearer-cut criterion for the purpose. We may conclude from the present study that strand symmetry would persist for up to as low as 6-mers, or as high as 9-mers, in individual prokaryotic genomes. As groups, strand symmetry would tend to persist for up to 8-mers in archaeal genomes, and up to 9-mers in bacterial genomes. In addition, it seems the extent of strand symmetry correlates with genomic GC content as well as mononucleotide symmetry levels, but not with taxonomic status such as phylum and class. Our results about the variations of higher-order strand symmetry would shed new light on the origin and evolution of the phenomenon.

## Data Availability

All datasets generated for this study are included in the manuscript and/or the supplementary files.

## Author Contributions

BH wrote the computer programs, analyzed data, and wrote the paper. L-FH analyzed data and wrote the paper. S-HZ designed the study, analyzed data, and wrote the paper.

### Conflict of Interest Statement

The authors declare that the research was conducted in the absence of any commercial or financial relationships that could be construed as a potential conflict of interest.
