# Supplementary material for: Evaluation of the Persistence of Higher-Order Strand Symmetry in Genomic Sequences by Novel Word Symmetry Distance Analysis
Source: Front Genet. 2019 Mar 7;10:148. doi: 10.3389/fgene.2019.00148 (PMC6416199; doi:10.3389/fgene.2019.00148)
Supplement: Supplementary file 1 [file Data_Sheet_1.PDF]

Supplementary material 2-2-1. Symmetry indexes of the complete genomes and their corresponding random sequences

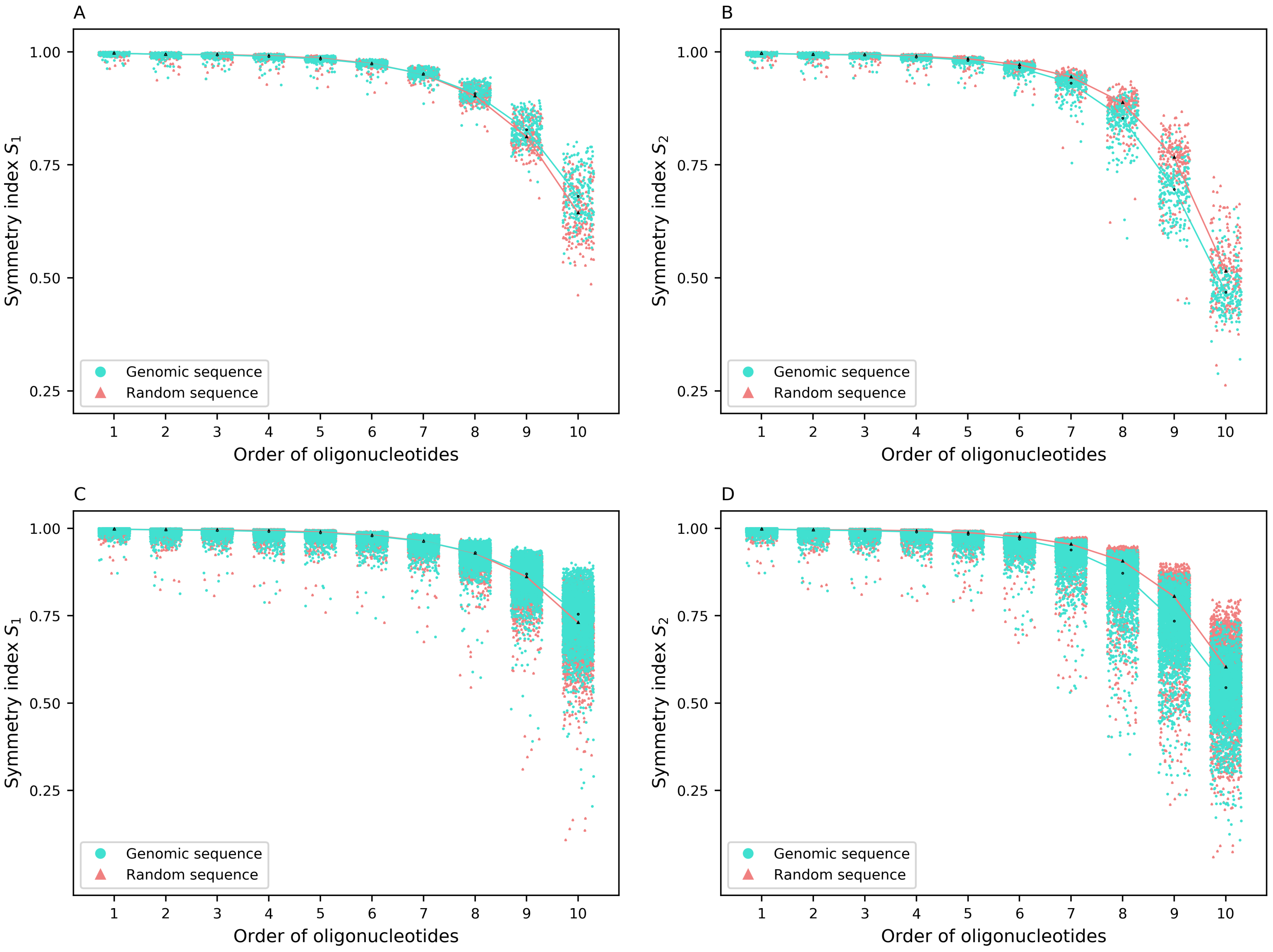

(A) S_1 and (B) S_2 of 206 archaeal genomes and their corresponding random sequences. (C) S_1 and (D) S_2 of 2659 bacterial genomes and their corresponding random sequences. Data dots are horizontally dispersed within the range of an order for better visualization. Medians for genomic sequences and random sequences, respectively, are marked for each order.

Supplementary material 2-2-2. Details of the quantile and fence values indicated in Fig. 1

Order		A					B					C					D				
		Q_1	Q_2	Q_3	Lower fence	Upper fence	Q_1	Q_2	Q_3	Lower fence	Upper fence	Q_1	Q_2	Q_3	Lower fence	Upper fence	Q_1	Q_2	Q_3	Lower fence	Upper fence
**1**	Genomic	0.9940	0.9967	0.9983	0.9876	0.9998	0.9939	0.9966	0.9983	0.9873	0.9998	0.9956	0.9976	0.9987	0.9910	0.9999	0.9954	0.9975	0.9986	0.9906	0.9999
	Random	0.9940	0.9967	0.9983	0.9876	0.9998	0.9939	0.9966	0.9983	0.9873	0.9998	0.9956	0.9976	0.9987	0.9910	0.9999	0.9954	0.9975	0.9986	0.9906	0.9999
**2**	Genomic	0.9905	0.9943	0.9967	0.9814	0.9991	0.9902	0.9942	0.9965	0.9807	0.9991	0.9924	0.9956	0.9971	0.9853	0.9996	0.9921	0.9954	0.9970	0.9849	0.9995
	Random	0.9905	0.9943	0.9971	0.9806	0.9994	0.9898	0.9942	0.9969	0.9793	0.9993	0.9926	0.9960	0.9977	0.9849	0.9998	0.9924	0.9959	0.9976	0.9846	0.9997
**3**	Genomic	0.9897	0.9932	0.9952	0.9816	0.9981	0.9893	0.9928	0.9948	0.9810	0.9978	0.9912	0.9943	0.9960	0.9840	0.9984	0.9901	0.9934	0.9954	0.9822	0.9983
	Random	0.9904	0.9939	0.9962	0.9816	0.9985	0.9896	0.9937	0.9960	0.9800	0.9982	0.9925	0.9956	0.9971	0.9856	0.9991	0.9921	0.9954	0.9968	0.9849	0.9987
**4**	Genomic	0.9861	0.9897	0.9921	0.9770	0.9961	0.9849	0.9886	0.9911	0.9755	0.9950	0.9875	0.9913	0.9935	0.9786	0.9971	0.9853	0.9898	0.9923	0.9749	0.9962
	Random	0.9876	0.9912	0.9933	0.9791	0.9963	0.9867	0.9904	0.9930	0.9773	0.9960	0.9898	0.9933	0.9951	0.9818	0.9978	0.9890	0.9929	0.9945	0.9806	0.9971
**5**	Genomic	0.9811	0.9842	0.9870	0.9722	0.9917	0.9773	0.9813	0.9841	0.9670	0.9888	0.9827	0.9872	0.9899	0.9718	0.9953	0.9775	0.9834	0.9868	0.9637	0.9931
	Random	0.9829	0.9859	0.9884	0.9746	0.9920	0.9806	0.9845	0.9869	0.9711	0.9906	0.9854	0.9895	0.9915	0.9763	0.9959	0.9834	0.9878	0.9901	0.9733	0.9940
**6**	Genomic	0.9693	0.9731	0.9768	0.9580	0.9843	0.9600	0.9650	0.9687	0.9470	0.9786	0.9724	0.9788	0.9826	0.9570	0.9917	0.9597	0.9690	0.9748	0.9370	0.9859
	Random	0.9711	0.9739	0.9784	0.9600	0.9836	0.9666	0.9713	0.9742	0.9552	0.9820	0.9749	0.9806	0.9838	0.9614	0.9920	0.9699	0.9769	0.9806	0.9539	0.9873
**7**	Genomic	0.9458	0.9510	0.9582	0.9272	0.9702	0.9214	0.9309	0.9375	0.8972	0.9594	0.9527	0.9627	0.9685	0.9288	0.9851	0.9204	0.9386	0.9503	0.8756	0.9702
	Random	0.9460	0.9504	0.9587	0.9268	0.9685	0.9365	0.9445	0.9491	0.9176	0.9665	0.9539	0.9634	0.9689	0.9313	0.9844	0.9413	0.9543	0.9615	0.9111	0.9752
**8**	Genomic	0.8984	0.9075	0.9212	0.8642	0.9432	0.8337	0.8534	0.8703	0.7787	0.9183	0.9125	0.9307	0.9413	0.8694	0.9719	0.8327	0.8716	0.8975	0.7355	0.9383
	Random	0.8934	0.9027	0.9190	0.8551	0.9398	0.8712	0.8876	0.8977	0.8313	0.9344	0.9106	0.9291	0.9387	0.8684	0.9692	0.8802	0.9065	0.9218	0.8177	0.9507
**9**	Genomic	0.8065	0.8275	0.8517	0.7387	0.8922	0.6598	0.6959	0.7248	0.5622	0.8223	0.8371	0.8697	0.8893	0.7586	0.9473	0.6788	0.7346	0.7835	0.5216	0.8737
	Random	0.7917	0.8121	0.8400	0.7191	0.8817	0.7312	0.7665	0.7884	0.6455	0.8680	0.8267	0.8614	0.8805	0.7459	0.9397	0.7480	0.8050	0.8383	0.6125	0.9007
**10**	Genomic	0.6427	0.6805	0.7282	0.5324	0.8003	0.4435	0.4685	0.5015	0.3565	0.5884	0.7003	0.7548	0.7938	0.5601	0.9022	0.4892	0.5443	0.5883	0.3405	0.7350
	Random	0.6036	0.6440	0.6949	0.4665	0.7678	0.4673	0.5149	0.5561	0.3341	0.6892	0.6710	0.7301	0.7676	0.5261	0.8823	0.5191	0.6032	0.6611	0.3061	0.7958

* Q_1: the first quartile, the lowest 25% of data; Q_2: the second quartile, the median of the data; Q_3: the third quartile, the highest 75% of data; Lower fence: equal to (Q_1 - 1.5IQR) or the minimum value of data; Upper fence: equal to (Q_3 + 1.5IQR) or the maximum value of data; IQR (interquartile range) = Q_3 - Q_1; the Lower fence is the "lower limit" and the Upper fence is the "upper limit" of data, and any data lying outside these defined bounds can be considered as an outlier.

Supplementary material 2-2-3. Paired differences between symmetry index values

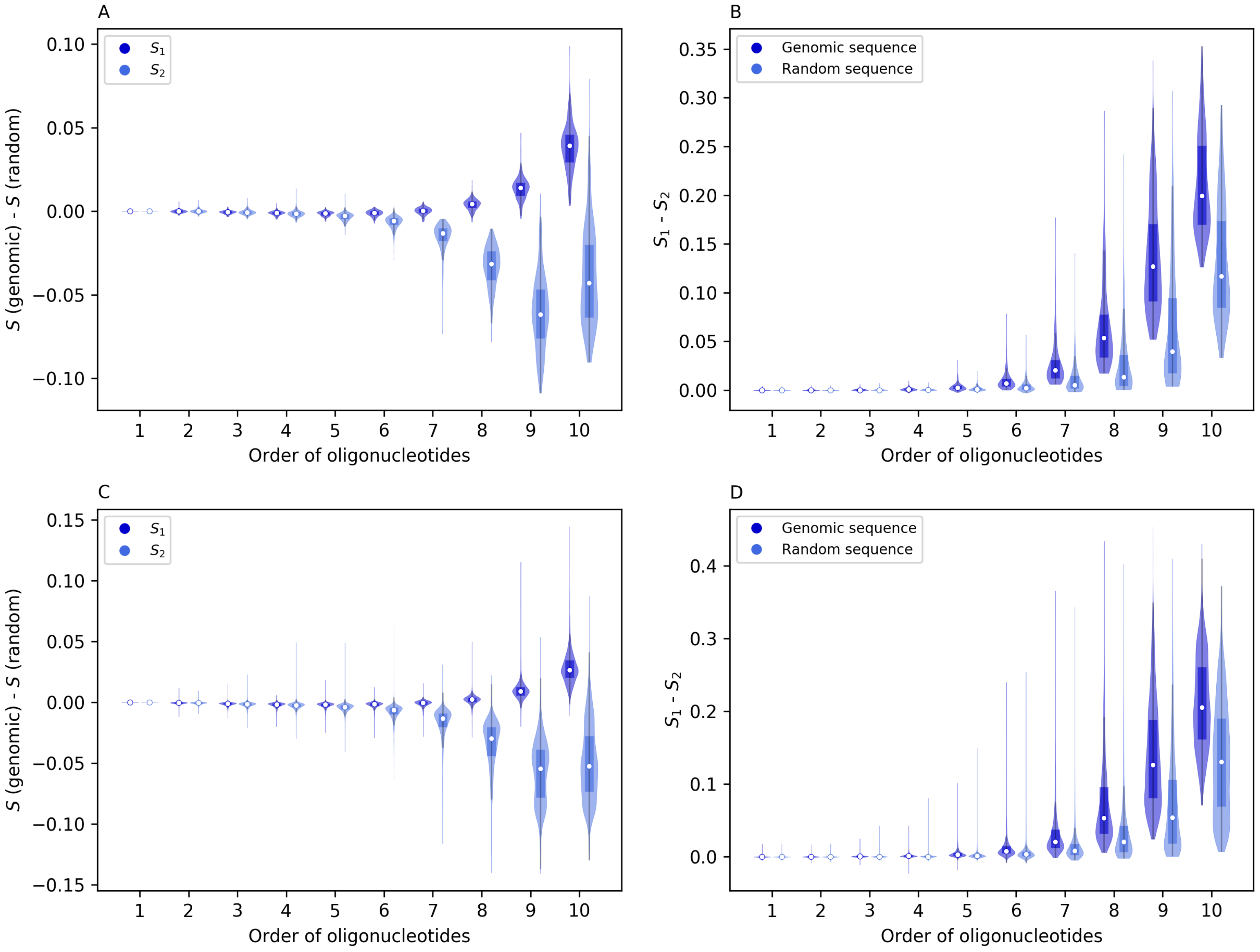

(A) Paired differences of S_1 and S_2, respectively, between 206 archaeal genomes and their corresponding random sequences. (B) Paired differences between S_1 and S_2 of 206archaeal genomes and their corresponding random sequences respectively. (C) Paireddifferences of S_1 and S_2, respectively, between 2659 bacterial genomes and their corresponding random sequences. (D) Paired differences between S_1 and S_2 of 2659 bacterial genomes and their corresponding random sequences respectively. S (genomic) and S (random) denote symmetry index for a genomic sequence and its corresponding random sequence respectively. The rectangles in the violin plot indicate the interquartile range of the data at different orders. Medians are marked with dots.
