# Supplementary material for: Evaluation of the Persistence of Higher-Order Strand Symmetry in Genomic Sequences by Novel Word Symmetry Distance Analysis
Source: Front Genet. 2019 Mar 7;10:148. doi: 10.3389/fgene.2019.00148 (PMC6416199; doi:10.3389/fgene.2019.00148)
Supplement: Supplementary file 2 [file Data_Sheet_2.PDF]

Supplementary material 3-2-1. Various word symmetry distances for 206 archaeal genomes and their corresponding random sequences

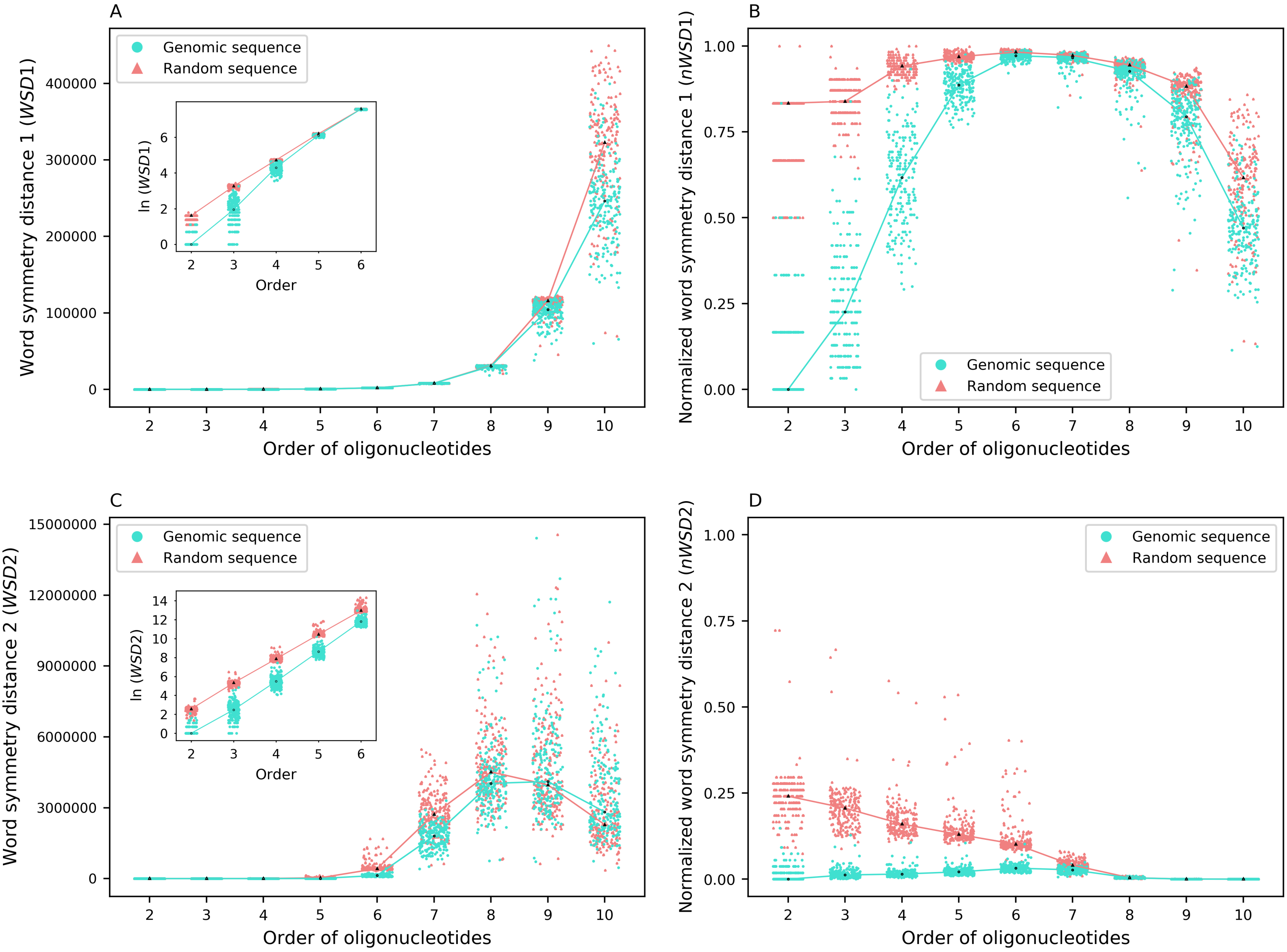

(A) *WSD*1. (B) n*WSD*1. (C) *WSD*2. (D) n*WSD*2. The values of *WSD* are presented in natural logarithm in insets (A and C). For other explanations see the legend of Supplementarymaterial 2-2-1.

Supplementary material 3-2-2. Details of the quantile and fence values indicated in Fig. 2

		A					B					C					D				
**Order**		Q_1	Q_2	Q_3	Lower fence	Upper fence	Q_1	Q_2	Q_3	Lower fence	Upper fence	Q_1	Q_2	Q_3	Lower fence	Upper fence	Q_1	Q_2	Q_3	Lower fence	Upper fence
**2**	Genomic	0	0	1	0	3	0.00	0.00	0.17	0.00	0.42	0	0	1	0	3	0.0E+00	0.0E+00	1.9E-02	0.0E+00	4.6E-02
	Random	4	5	5	3	6	0.67	0.83	0.83	0.50	1.00	11	13	15	5	21	2.0E-01	2.4E-01	2.8E-01	9.3E-02	3.9E-01
**3**	Genomic	4	7	11	0	22	0.13	0.23	0.35	0.00	0.69	6	12	20	0	41	6.0E-03	1.2E-02	2.0E-02	0.0E+00	4.1E-02
	Random	25	26	27	22	30	0.81	0.84	0.87	0.71	0.97	170	205	230	88	320	1.7E-01	2.1E-01	2.3E-01	8.9E-02	3.2E-01
**4**	Genomic	62	74	85	35	112	0.52	0.62	0.71	0.29	0.93	166	241	338	59	595	1.0E-02	1.5E-02	2.1E-02	3.6E-03	3.7E-02
	Random	110	113	114	105	120	0.92	0.94	0.95	0.88	1.00	2248	2593	3078	1703	4321	1.4E-01	1.6E-01	1.9E-01	1.1E-01	2.7E-01
**5**	Genomic	436	453	467	390	498	0.85	0.89	0.91	0.76	0.97	4048	5548	7177	2365	11870	1.5E-02	2.1E-02	2.7E-02	9.0E-03	4.5E-02
	Random	492	495	498	483	507	0.96	0.97	0.97	0.95	0.99	31352	33948	38457	26081	49116	1.2E-01	1.3E-01	1.5E-01	1.0E-01	1.9E-01
**6**	Genomic	1943	1959	1970	1903	1998	0.96	0.97	0.98	0.94	0.99	99290	133722	157434	71035	244649	2.4E-02	3.2E-02	3.8E-02	1.7E-02	5.8E-02
	Random	1975	1980	1986	1959	2001	0.98	0.98	0.99	0.97	0.99	404289	424534	471080	304102	571267	9.6E-02	1.0E-01	1.1E-01	7.3E-02	1.4E-01
**7**	Genomic	7871	7910	7942	7766	8048	0.96	0.97	0.97	0.95	0.98	1437893	1807544	2123536	409428	3152002	2.1E-02	2.7E-02	3.2E-02	6.1E-03	4.7E-02
	Random	7933	7967	7989	7849	8072	0.97	0.97	0.98	0.96	0.99	2335717	2724551	3325699	850745	4810672	3.5E-02	4.1E-02	5.0E-02	1.3E-02	7.2E-02
**8**	Genomic	29762	30239	30574	28545	31543	0.91	0.93	0.94	0.87	0.97	3417965	4030549	4725573	1456552	6686986	3.2E-03	3.8E-03	4.4E-03	1.4E-03	6.2E-03
	Random	30594	30841	31022	29951	31603	0.94	0.94	0.95	0.92	0.97	3695555	4516770	5512171	970631	8237095	3.4E-03	4.2E-03	5.1E-03	9.0E-04	7.7E-03
**9**	Genomic	97618	104109	109398	79948	120363	0.74	0.79	0.83	0.61	0.92	3404891	4109413	5661534	723868	9046500	2.0E-04	2.4E-04	3.3E-04	4.2E-05	5.3E-04
	Random	111647	115672	117377	103052	122682	0.85	0.88	0.90	0.79	0.94	3187933	3969825	5901207	628915	9971118	1.9E-04	2.3E-04	3.4E-04	3.7E-05	5.8E-04
**10**	Genomic	214269	246319	272224	127336	359157	0.41	0.47	0.52	0.24	0.69	2199238	2822025	4489390	468453	7924618	8.0E-06	1.0E-05	1.6E-05	1.7E-06	2.9E-05
	Random	275057	322777	354324	156156	449637	0.53	0.62	0.68	0.30	0.86	1779480	2284146	3824954	350918	6893164	6.5E-06	8.3E-06	1.4E-05	1.3E-06	2.5E-05

* Q_1: the first quartile, the lowest 25% of data; Q_2: the second quartile, the median of the data; Q_3: the third quartile, the highest 75% of data; Lower fence: equal to (Q_1 - 1.5IQR) or the minimum value of data; Upper fence: equal to (Q_3 + 1.5IQR) or the maximum value of data; IQR (interquartile range) = Q_3 - Q_1; the Lower fence is the "lower limit" and the Upper fence is the "upper limit" of data, and any data lying outside these defined bounds can be considered as an outlier.

Supplemenatry material 3-2-3. Various word symmetry distances for 2659 bacterial genomes and their corresponding random sequences

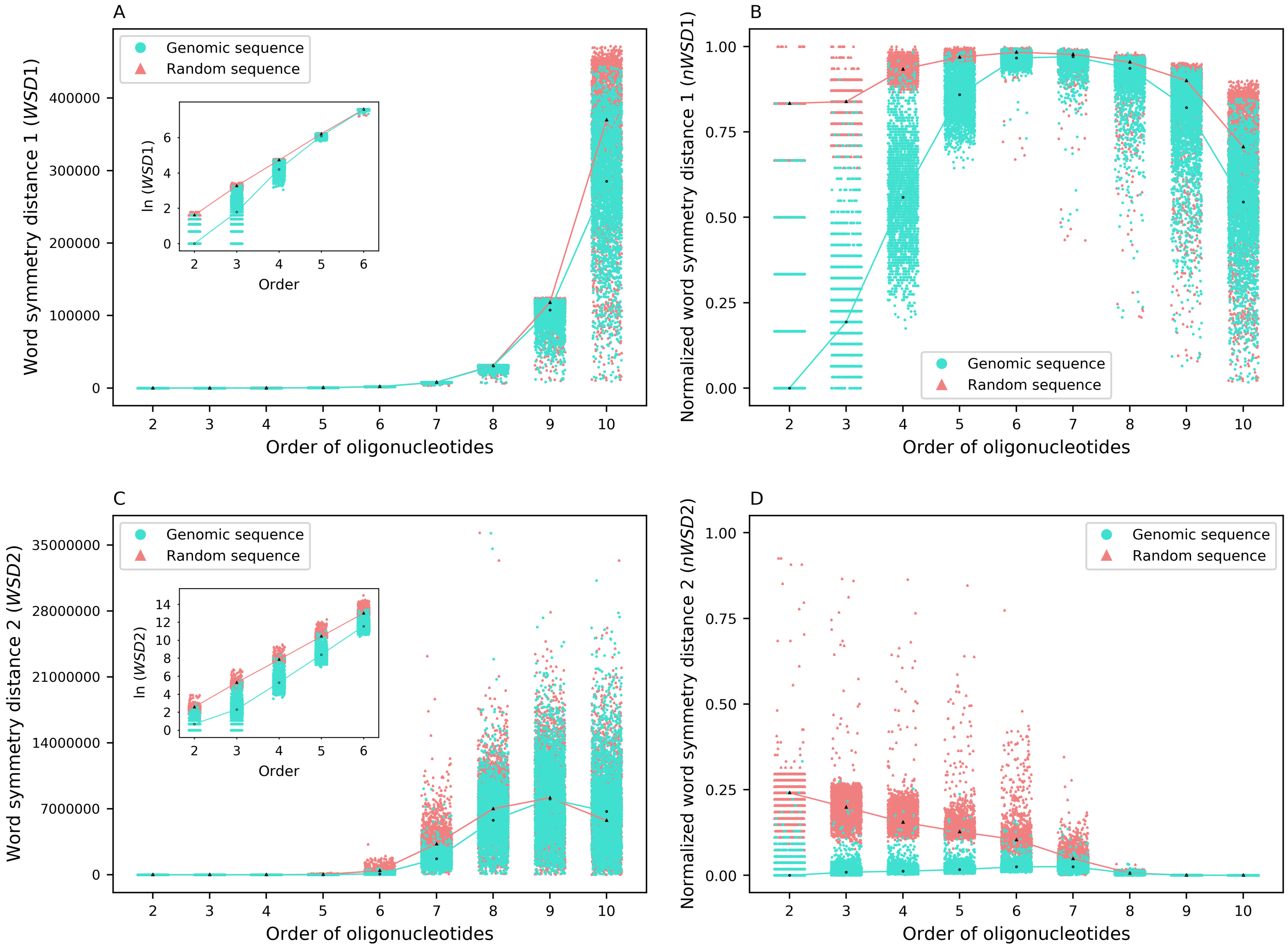

(A) *WSD*1. (B) n*WSD*1. (C) *WSD*2. (D) n*WSD*2. For other explanations see the legendof Supplementary material 2-2-1 and 3-2-1.

Supplementary material 3-2-4. Details of the quantile and fence values indicated in Fig. 3

		A					B					C					D				
**Order**		Q_1	Q_2	Q_3	Lower fence	Upper fence	Q_1	Q_2	Q_3	Lower fence	Upper fence	Q_1	Q_2	Q_3	Lower fence	Upper fence	Q_1	Q_2	Q_3	Lower fence	Upper fence
**2**	Genomic	0	0	1	0	3	0.00	0.00	0.17	0.00	0.42	0	0	1	0	3	0.0E+00	0.0E+00	1.9E-02	0.0E+00	4.6E-02
	Random	4	5	5	3	6	0.67	0.83	0.83	0.42	1.00	11	13	15	5	21	2.0E-01	2.4E-01	2.8E-01	9.3E-02	3.9E-01
**3**	Genomic	4	6	9	0	17	0.13	0.19	0.29	0.00	0.53	6	9	15	0	29	6.0E-03	9.1E-03	1.5E-02	0.0E+00	2.9E-02
	Random	25	26	27	22	30	0.81	0.84	0.87	0.71	0.97	166	197	226	77	316	1.7E-01	2.0E-01	2.3E-01	7.8E-02	3.2E-01
**4**	Genomic	56	67	79	22	114	0.47	0.56	0.66	0.18	0.95	139	195	284	33	502	8.5E-03	1.2E-02	1.8E-02	2.0E-03	3.1E-02
	Random	110	112	114	104	120	0.92	0.93	0.95	0.87	1.00	2252	2513	2911	1622	3900	1.4E-01	1.6E-01	1.8E-01	1.0E-01	2.4E-01
**5**	Genomic	419	439	455	365	504	0.82	0.86	0.89	0.71	0.99	3208	4397	5800	1096	9689	1.2E-02	1.7E-02	2.2E-02	4.2E-03	3.7E-02
	Random	492	495	497	485	505	0.96	0.97	0.97	0.95	0.99	31571	33308	36744	23812	44504	1.2E-01	1.3E-01	1.4E-01	9.1E-02	1.7E-01
**6**	Genomic	1930	1948	1961	1884	2006	0.96	0.97	0.97	0.93	1.00	78217	104268	130307	33200	208443	1.9E-02	2.5E-02	3.1E-02	7.9E-03	5.0E-02
	Random	1976	1982	1988	1958	2006	0.98	0.98	0.99	0.97	1.00	415742	435204	459209	350540	524410	9.9E-02	1.0E-01	1.1E-01	8.4E-02	1.3E-01
**7**	Genomic	7871	7945	7993	7688	8141	0.96	0.97	0.98	0.94	0.99	1251579	1702628	2132707	68691	3454400	1.9E-02	2.5E-02	3.2E-02	1.0E-03	5.1E-02
	Random	7954	8001	8033	7836	8150	0.97	0.98	0.98	0.96	0.99	2539671	3287101	3972988	389694	6122964	3.8E-02	4.9E-02	5.9E-02	5.8E-03	9.1E-02
**8**	Genomic	29590	30564	31045	27408	31985	0.91	0.94	0.95	0.84	0.98	4261187	5800566	7582282	84813	12563925	4.0E-03	5.4E-03	7.1E-03	7.9E-05	1.2E-02
	Random	30717	31140	31386	29714	32090	0.94	0.95	0.96	0.91	0.98	4801428	7002316	9171797	122299	15727351	4.5E-03	6.5E-03	8.5E-03	1.1E-04	1.5E-02
**9**	Genomic	96021	107598	115159	67315	123913	0.73	0.82	0.88	0.51	0.95	5253772	8043039	10894895	83147	19356579	3.1E-04	4.7E-04	6.3E-04	4.8E-06	1.1E-03
	Random	111832	117985	120616	98655	124795	0.85	0.90	0.92	0.75	0.95	5025181	8169620	11223959	70616	20522127	2.9E-04	4.8E-04	6.5E-04	4.1E-06	1.2E-03
**10**	Genomic	230601	285364	340252	66124	443446	0.44	0.54	0.65	0.13	0.85	3727924	6719085	9529564	35337	18232023	1.4E-05	2.4E-05	3.5E-05	1.3E-07	6.6E-05
	Random	300094	369908	418302	122781	471893	0.57	0.71	0.80	0.23	0.90	3077711	5760295	8441480	22768	16487132	1.1E-05	2.1E-05	3.1E-05	8.3E-08	6.0E-05

* Q_1: the first quartile, the lowest 25% of data; Q_2: the second quartile, the median of the data; Q_3: the third quartile, the highest 75% of data; Lower fence: equal to (Q_1 - 1.5IQR) or the minimum value of data; Upper fence: equal to (Q_3 + 1.5IQR) or the maximum value of data; IQR (interquartile range) = Q_3 - Q_1; the Lower fence is the "lower limit" and the Upper fence is the "upper limit" of data, and any data lying outside these defined bounds can be considered as an outlier.

Supplementary material 3-2-5. Paired differences of word symmetry distance values between 206 archaeal genomes and their corresponding random sequences.

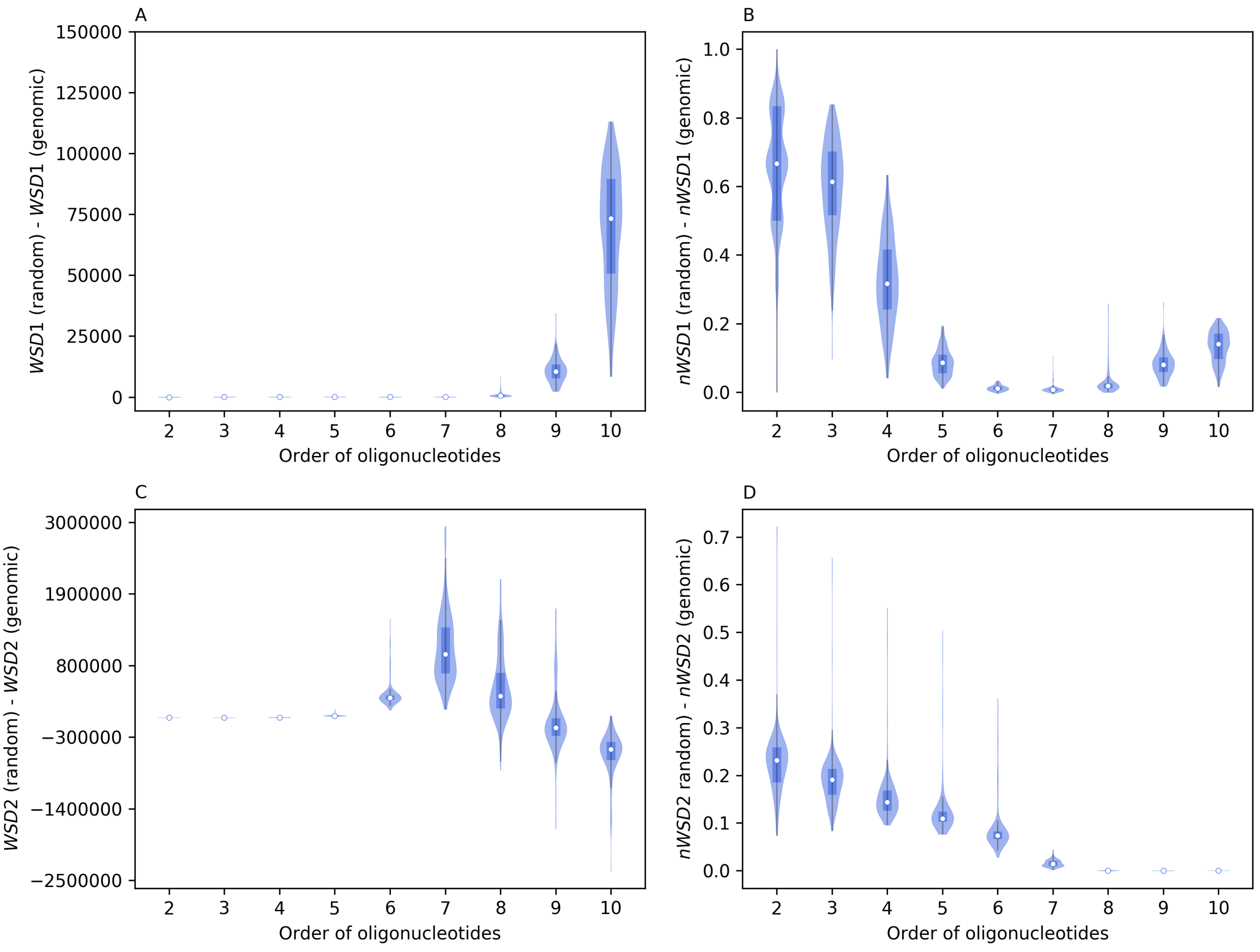

(A) Paired differences of *WSD*1. (B) Paired differences of n*WSD*1. (C) Paired differences of *WSD*2. (D) Paired differences of n*WSD*2. For other explanations see the legend of Supplementary material 2-2-3.

Supplementary material 3-2-6. Paired differences of word symmetry distance values between 2659 bacterial genomes and their corresponding random sequences.

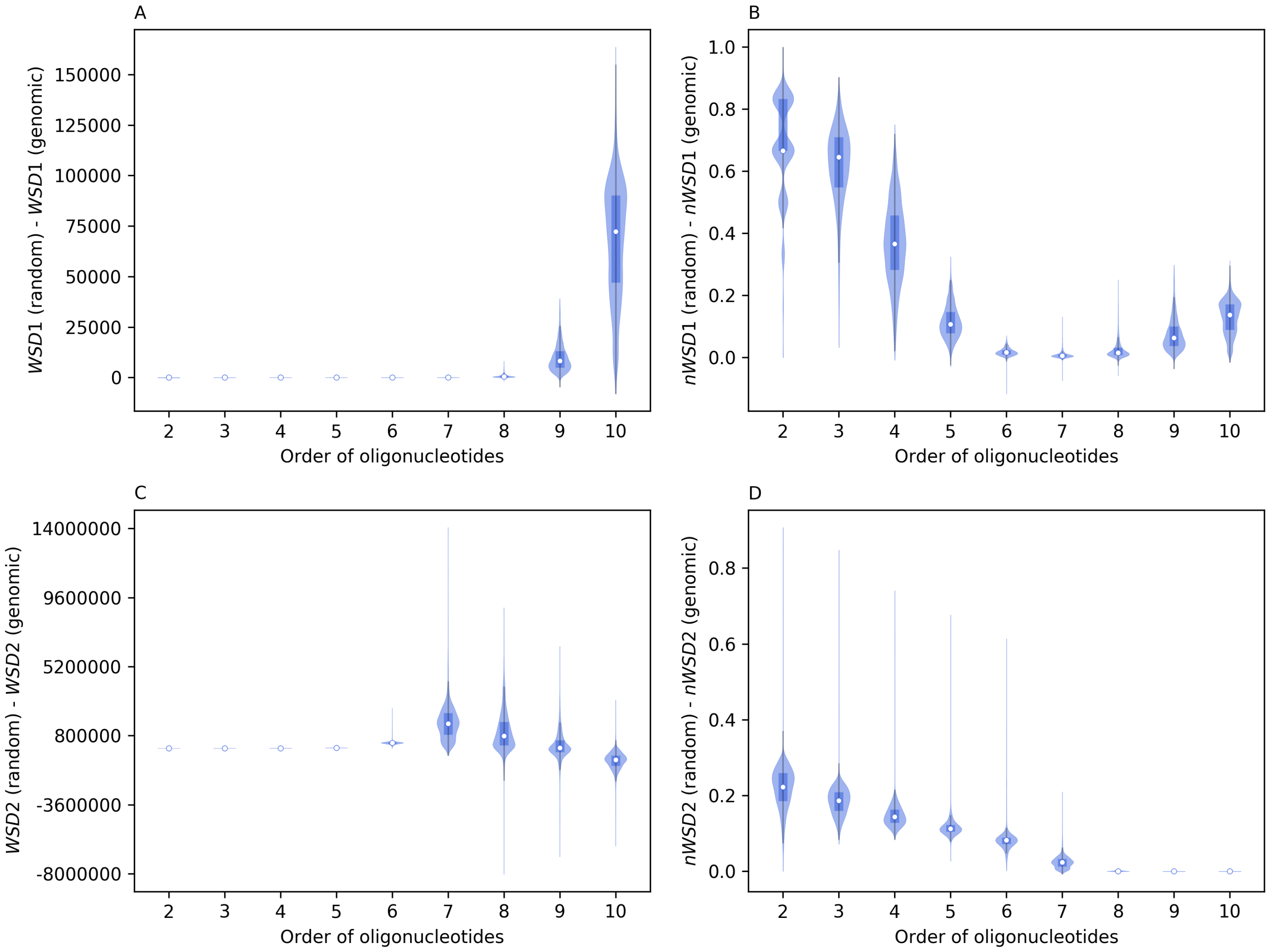

(A) Paired differences of *WSD*1. (B) Paired differences of n*WSD*1. (C) Paired differences of *WSD*2. (D) Paired differences of n*WSD*2. For other explanations see the legend of Supplementary material 2-2-3.
