# Supplementary material for: Evaluation of the Persistence of Higher-Order Strand Symmetry in Genomic Sequences by Novel Word Symmetry Distance Analysis
Source: Front Genet. 2019 Mar 7;10:148. doi: 10.3389/fgene.2019.00148 (PMC6416199; doi:10.3389/fgene.2019.00148)
Supplement: Supplementary file 3 [file Data_Sheet_3.PDF]

Supplementary material 4-2-1. *WSD*1 for groups of genomes (classified according to GC content)

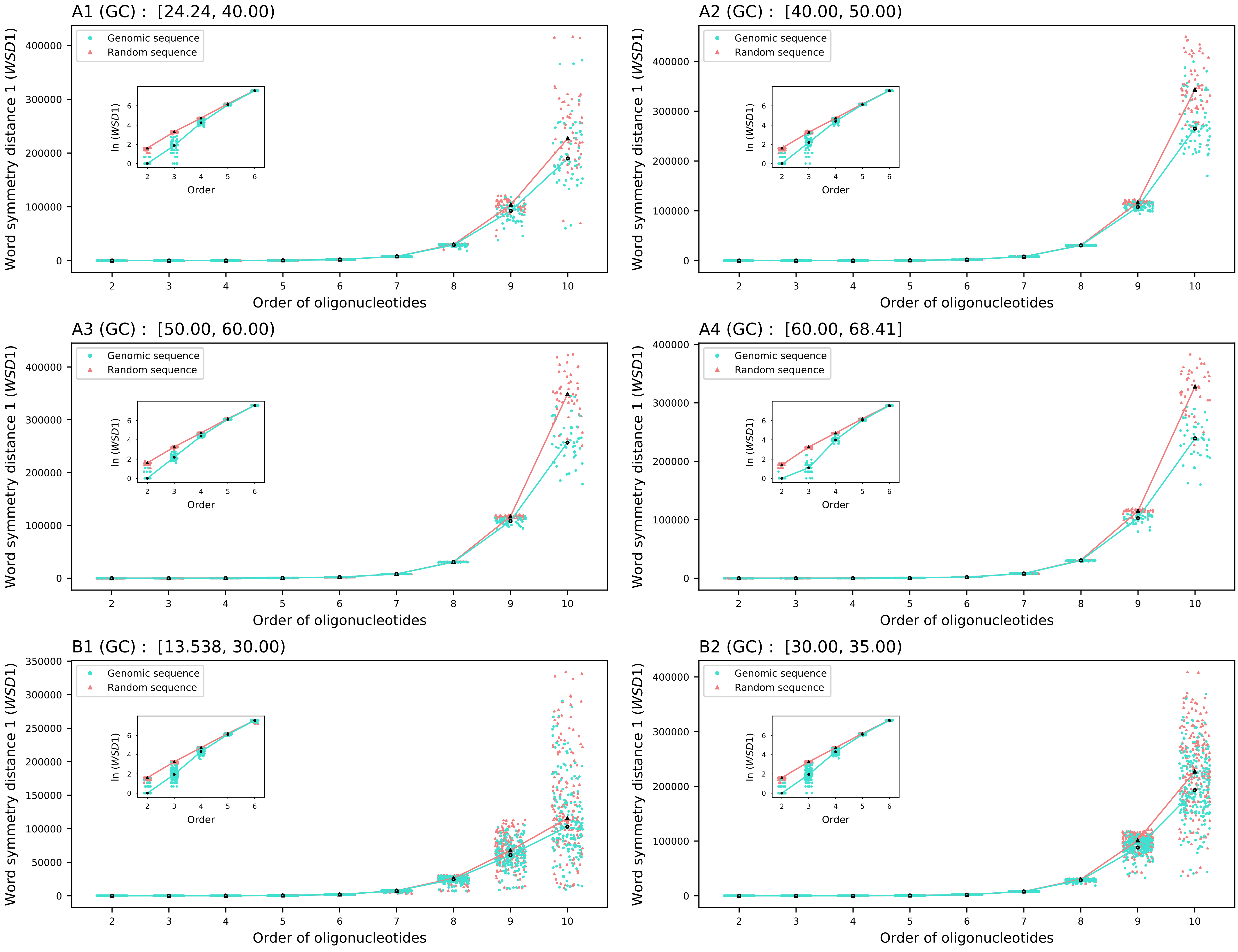

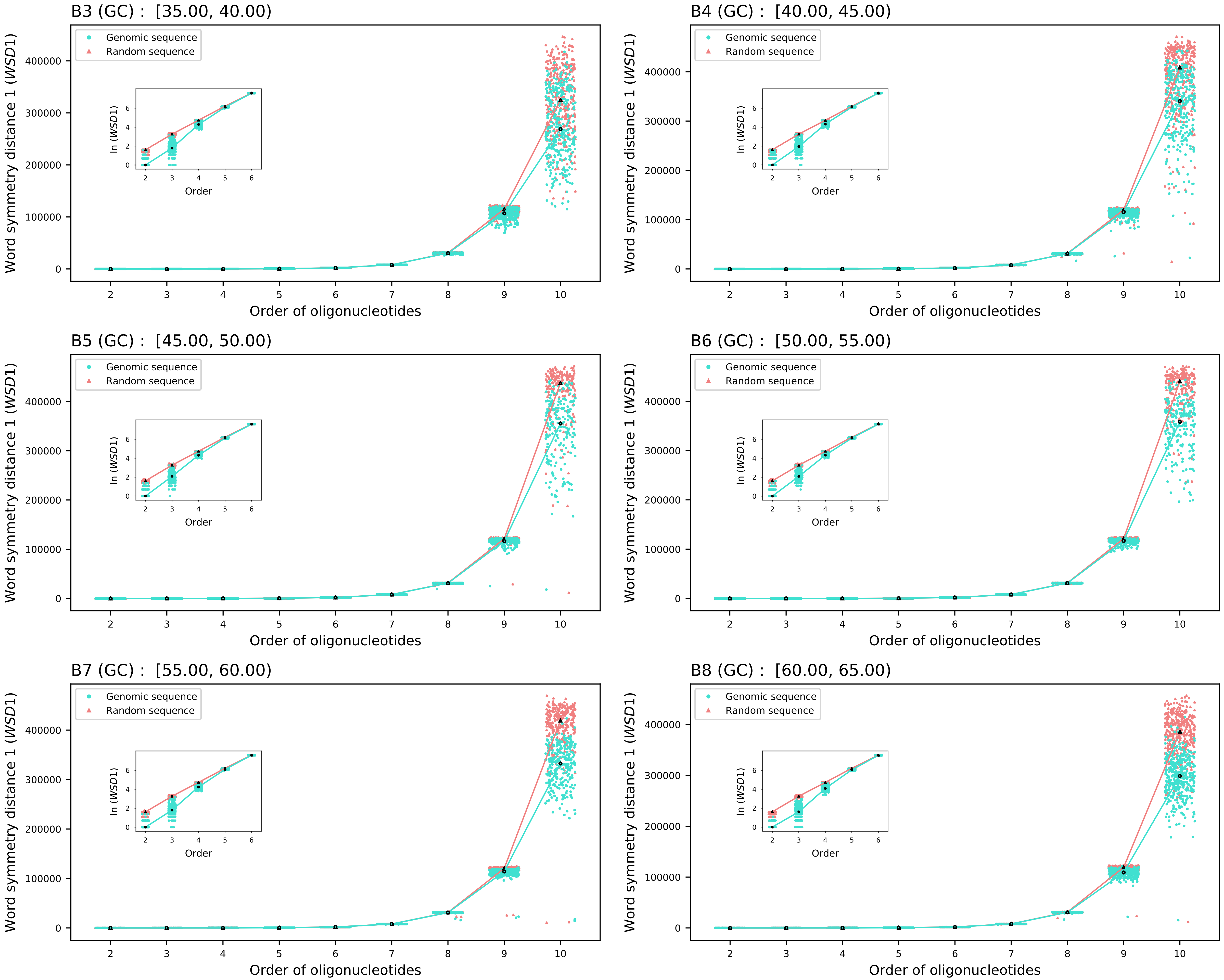
B3 (GC) : [35.00, 40.00)
B4 (GC) : [40.00, 45.00)
B5 (GC) : [45.00, 50.00)
B6 (GC) : [50.00, 55.00)
B7 (GC) : [55.00, 60.00)
B8 (GC) : [60.00, 65.00)
Genomic sequence
Random sequence
Word symmetry distance 1 (WSD1)
Order of oligonucleotides
ln (WSD1)
Order
0
100000
200000
300000
400000
2
3
4
5
6
7
8
9
10

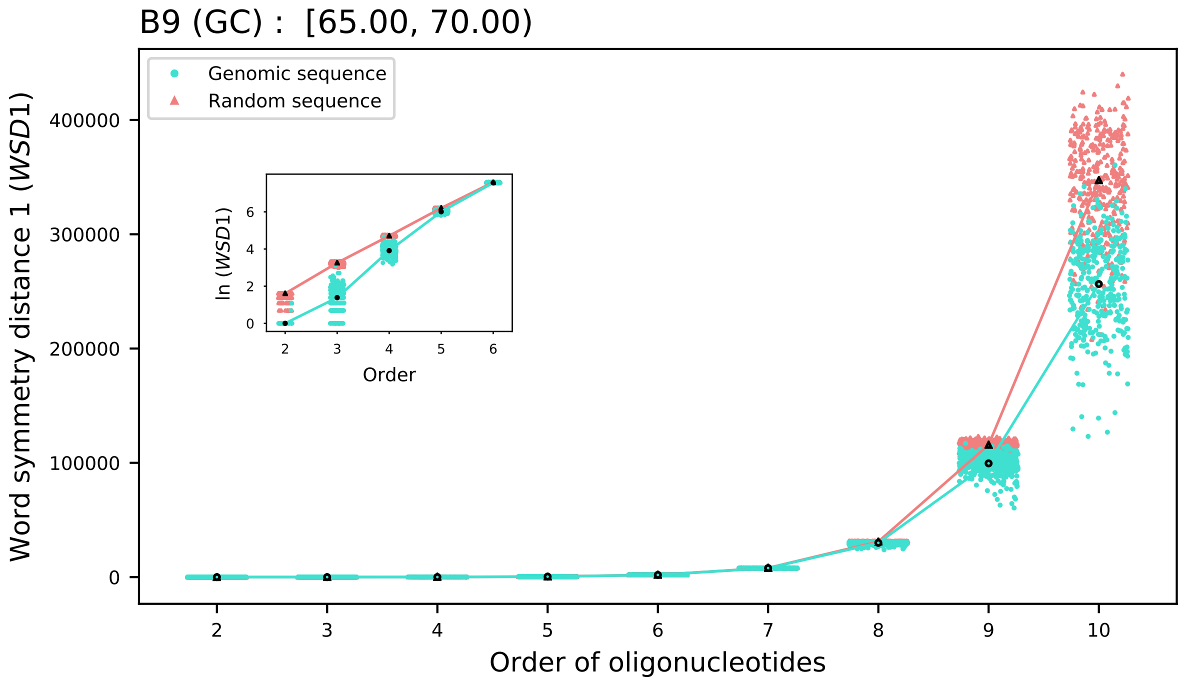
B9 (GC) : [65.00, 70.00)
Genomic sequence
Random sequence
Word symmetry distance 1 (WSD1)
0
100000
200000
300000
400000
2
3
4
5
6
7
8
9
10
Order of oligonucleotides
ln (WSD1)
Order

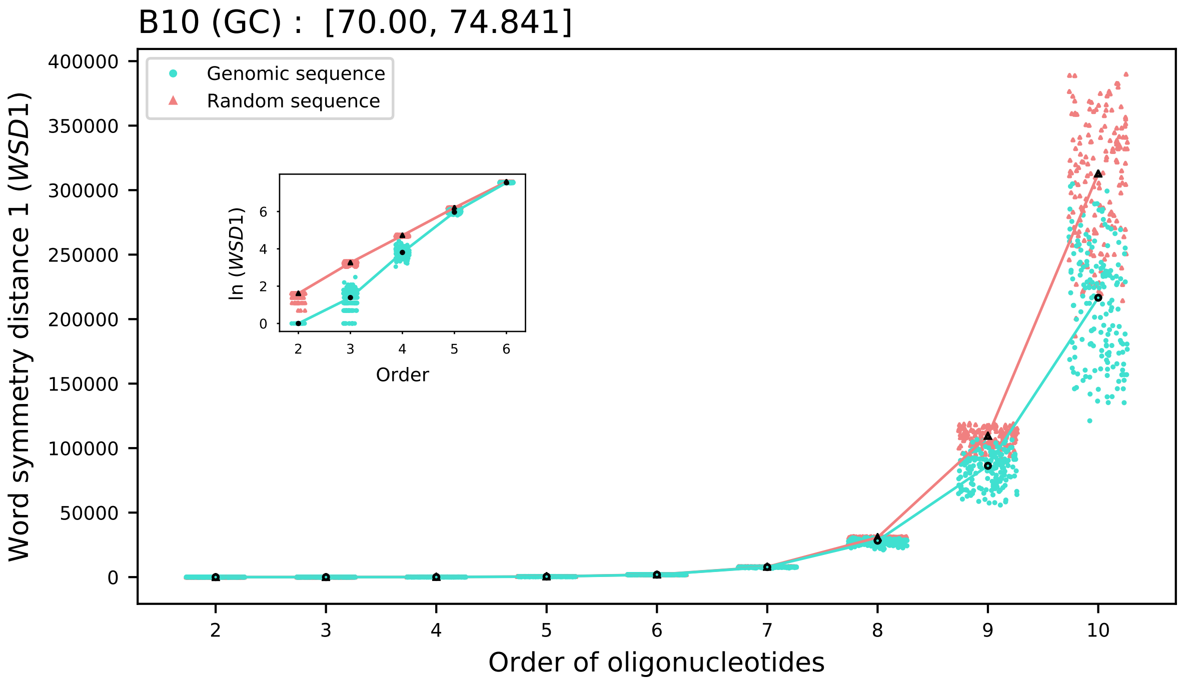
B10 (GC) : [70.00, 74.841]
Genomic sequence
Random sequence
Word symmetry distance 1 (WSD1)
0
50000
100000
150000
200000
250000
300000
350000
400000
2
3
4
5
6
7
8
9
10
Order of oligonucleotides
ln (WSD1)
Order

Supplementary material 4-2-2. *WSD*2 for groups of genomes (classified according to GC content)

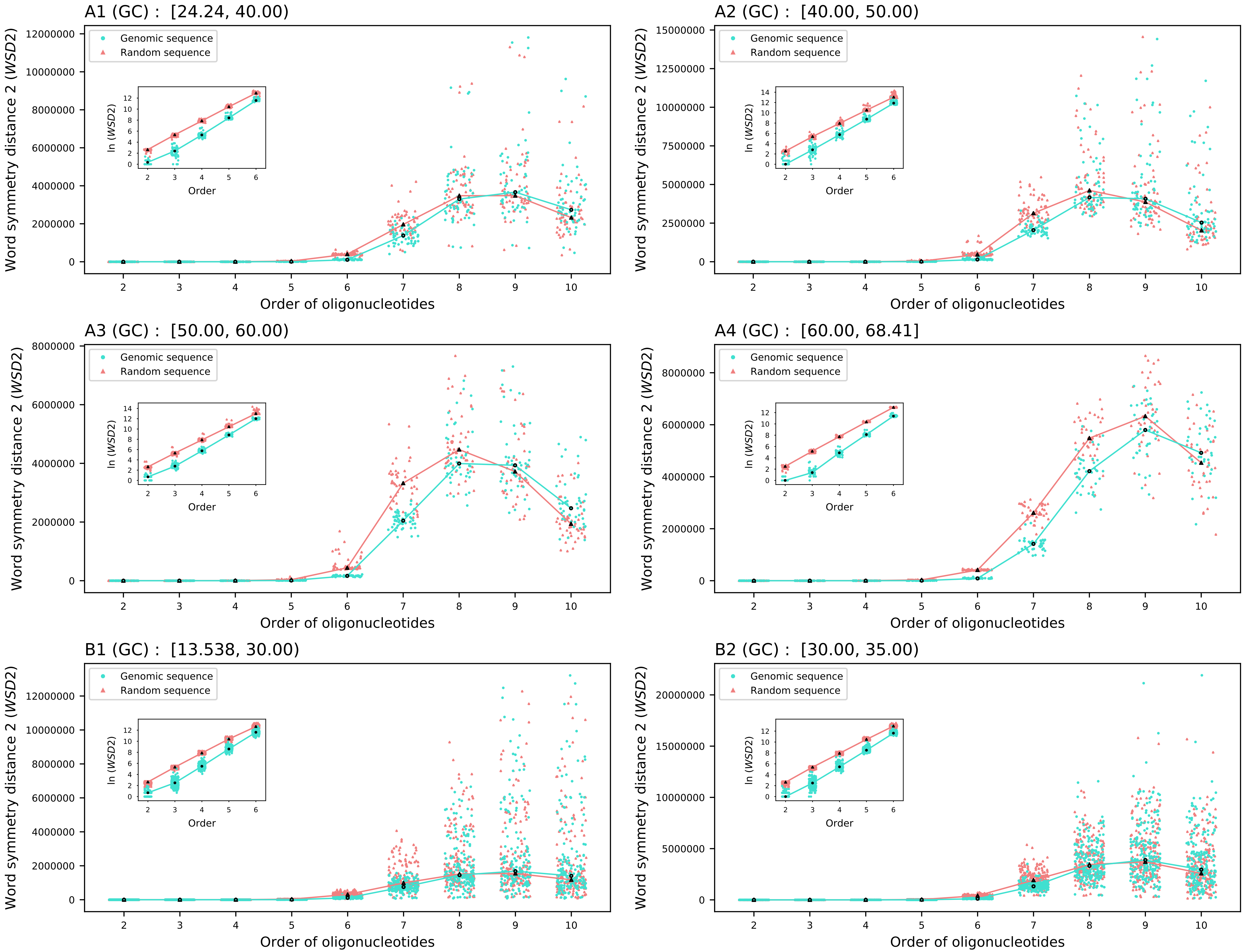

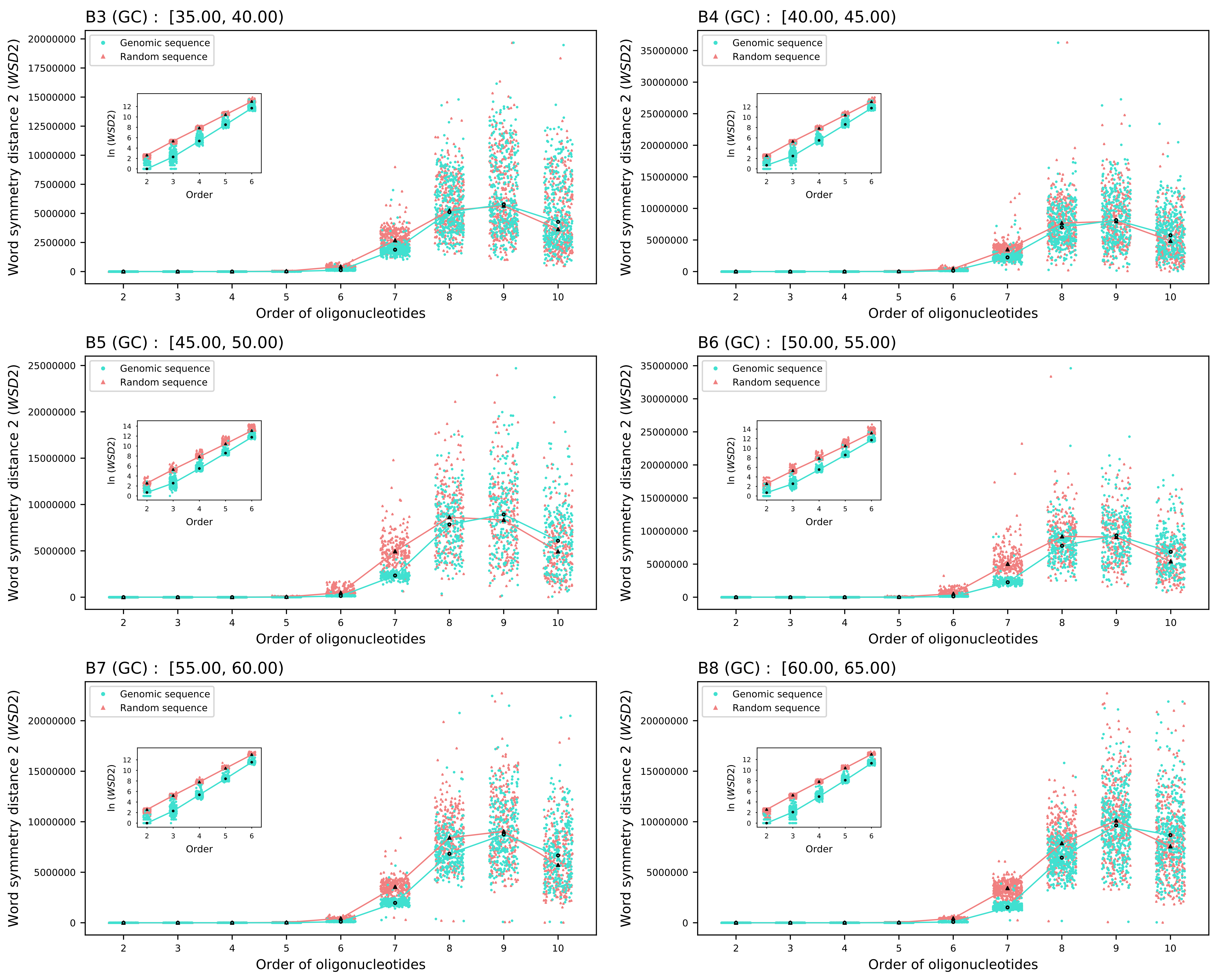
B3 (GC) : [35.00, 40.00)
B4 (GC) : [40.00, 45.00)
B5 (GC) : [45.00, 50.00)
B6 (GC) : [50.00, 55.00)
B7 (GC) : [55.00, 60.00)
B8 (GC) : [60.00, 65.00)
Genomic sequence
Random sequence
Word symmetry distance 2 (WSD2)
ln (WSD2)
Order
Order of oligonucleotides

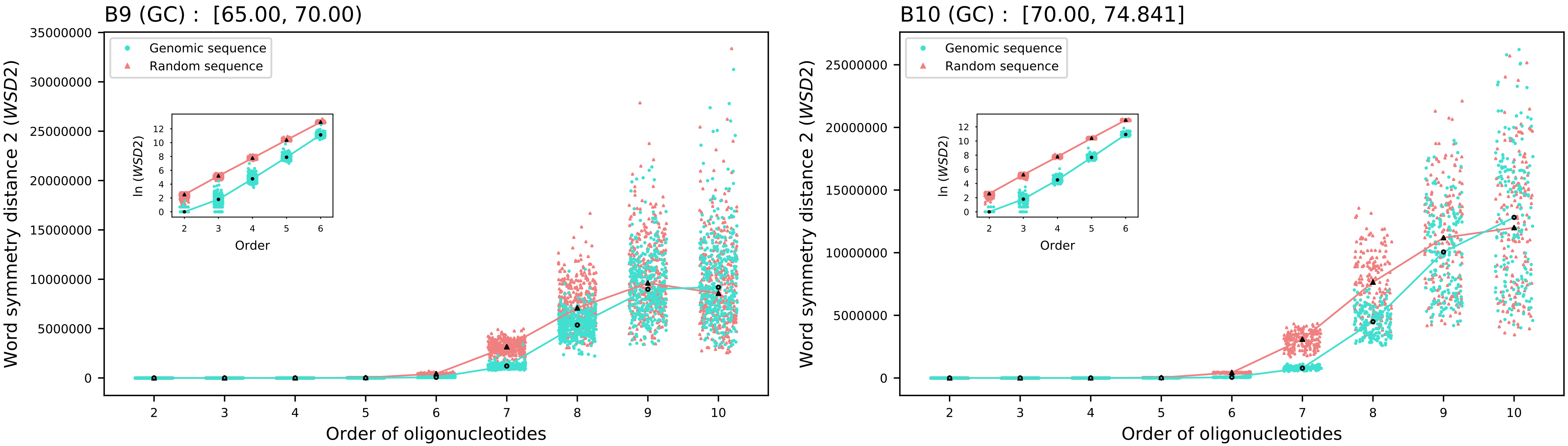
B9 (GC) : [65.00, 70.00)
B10 (GC) : [70.00, 74.841]
Genomic sequence
Random sequence
Word symmetry distance 2 (WSD2)
Order of oligonucleotides
ln (WSD2)
Order
