# Supplementary material for: Evaluation of the Persistence of Higher-Order Strand Symmetry in Genomic Sequences by Novel Word Symmetry Distance Analysis
Source: Front Genet. 2019 Mar 7;10:148. doi: 10.3389/fgene.2019.00148 (PMC6416199; doi:10.3389/fgene.2019.00148)
Supplement: Supplementary file 4 [file Data_Sheet_4.PDF]

Supplementary material 6-2-1. $WSD1$ for groups of genomes (classified according to S_1)

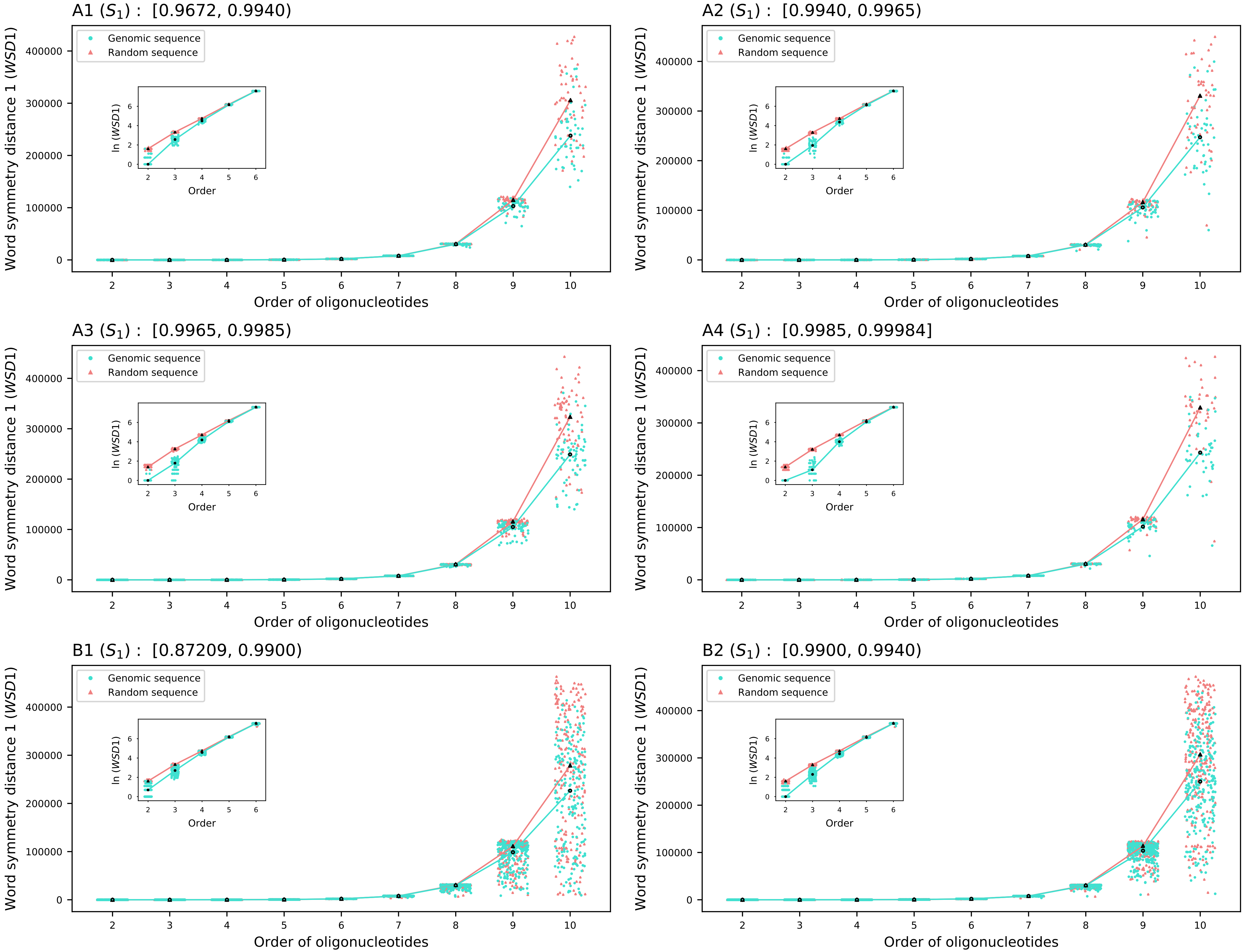

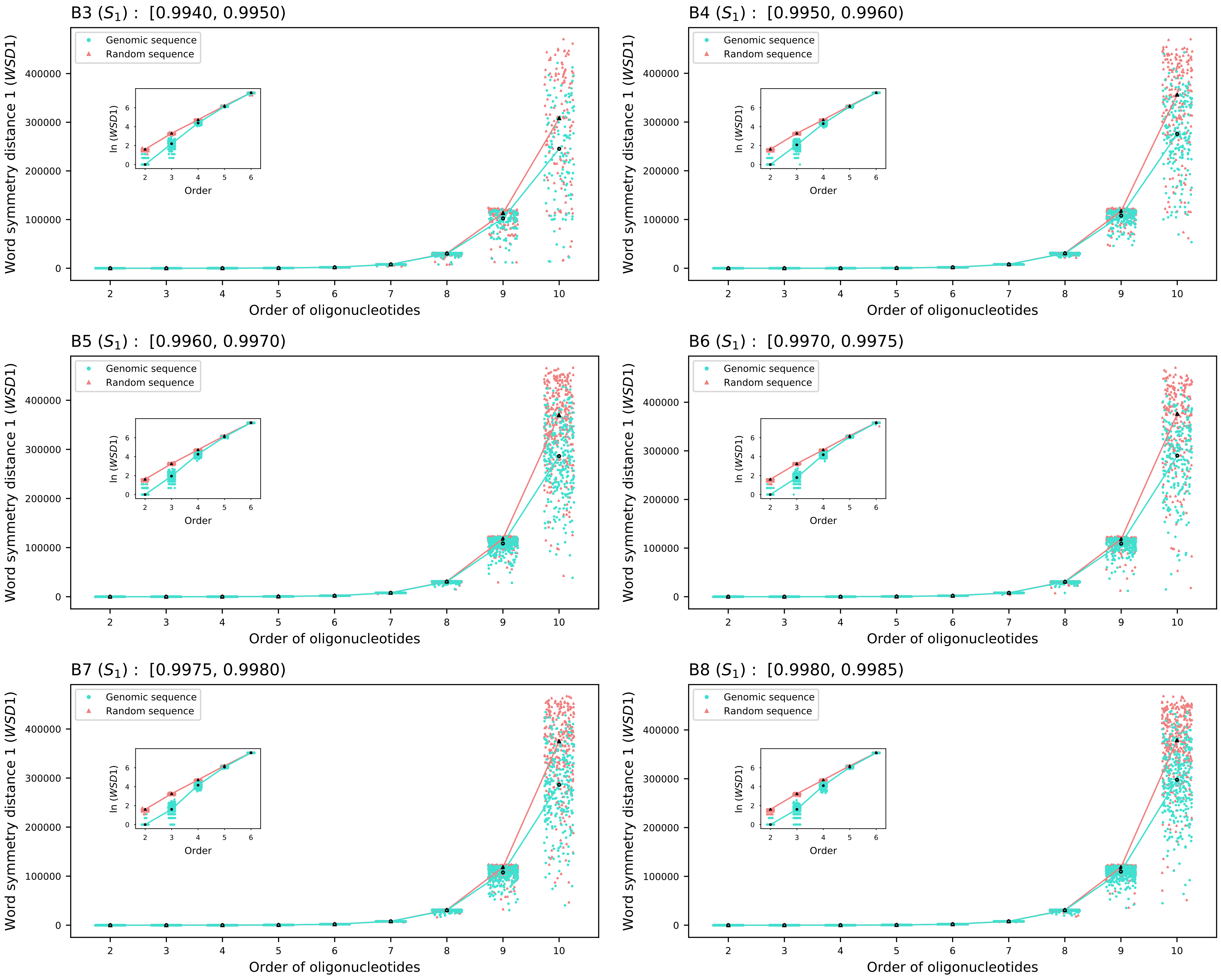
B3 (S_1) : [0.9940, 0.9950)
B4 (S_1) : [0.9950, 0.9960)
B5 (S_1) : [0.9960, 0.9970)
B6 (S_1) : [0.9970, 0.9975)
B7 (S_1) : [0.9975, 0.9980)
B8 (S_1) : [0.9980, 0.9985)
Genomic sequence
Random sequence
Word symmetry distance 1 ($WSD1$)
Order of oligonucleotides
ln ($WSD1$)
Order
0
100000
200000
300000
400000
2
3
4
5
6
7
8
9
10

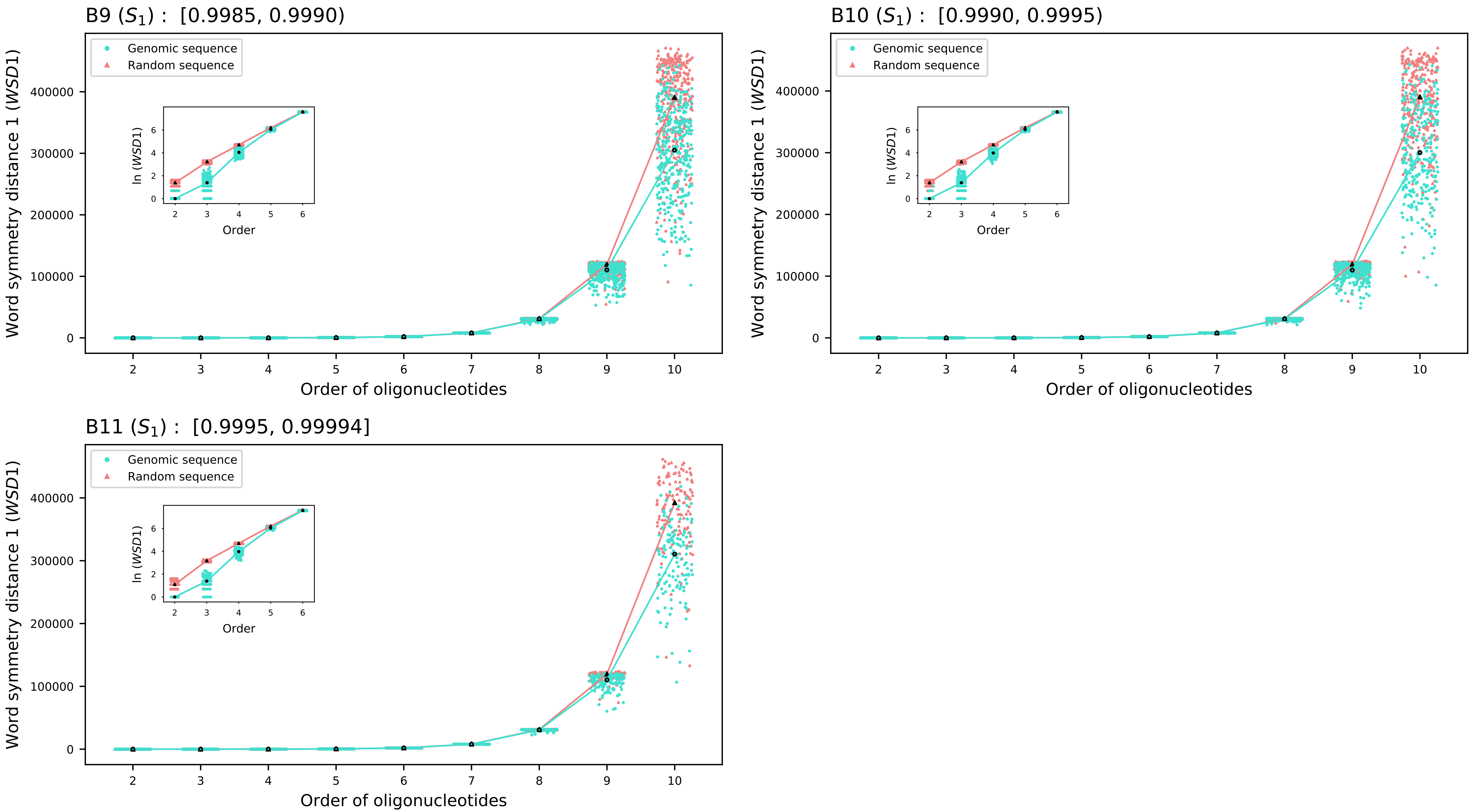

B9 (S_1) : [0.9985, 0.9990)
B10 (S_1) : [0.9990, 0.9995)
B11 (S_1) : [0.9995, 0.99994]
Genomic sequence
Random sequence
Word symmetry distance 1 ($WSD1$)
Order of oligonucleotides
ln ($WSD1$)
Order
400000
300000
200000
100000
0
2
3
4
5
6
7
8
9
10

Supplementary material 6-2-2. *WSD*2 for groups of genomes (classified according to S_1)

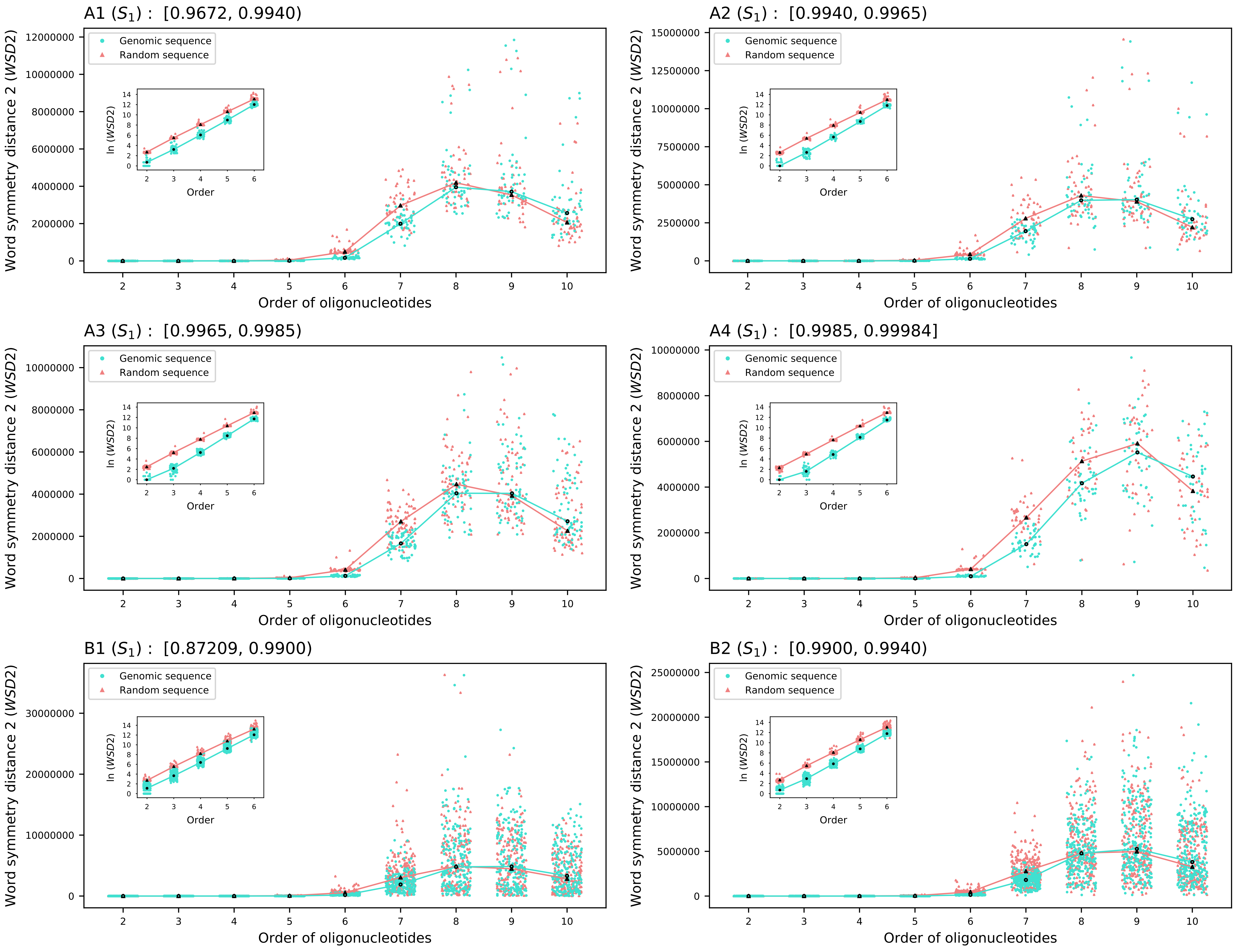

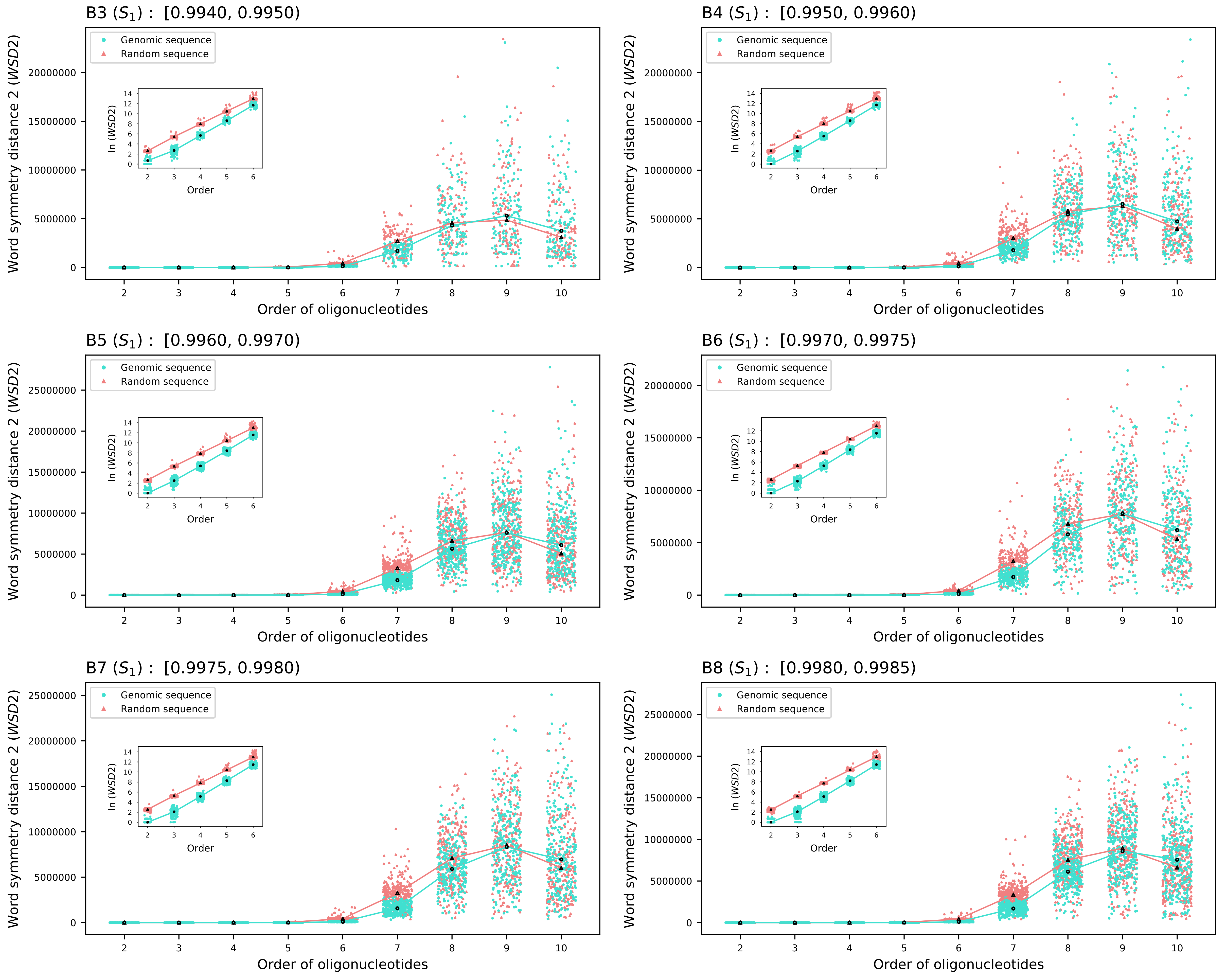
B3 (S_1) : [0.9940, 0.9950)
B4 (S_1) : [0.9950, 0.9960)
B5 (S_1) : [0.9960, 0.9970)
B6 (S_1) : [0.9970, 0.9975)
B7 (S_1) : [0.9975, 0.9980)
B8 (S_1) : [0.9980, 0.9985)
Genomic sequence
Random sequence
Word symmetry distance 2 (*WSD2*)
Order of oligonucleotides
ln (*WSD2*)
Order

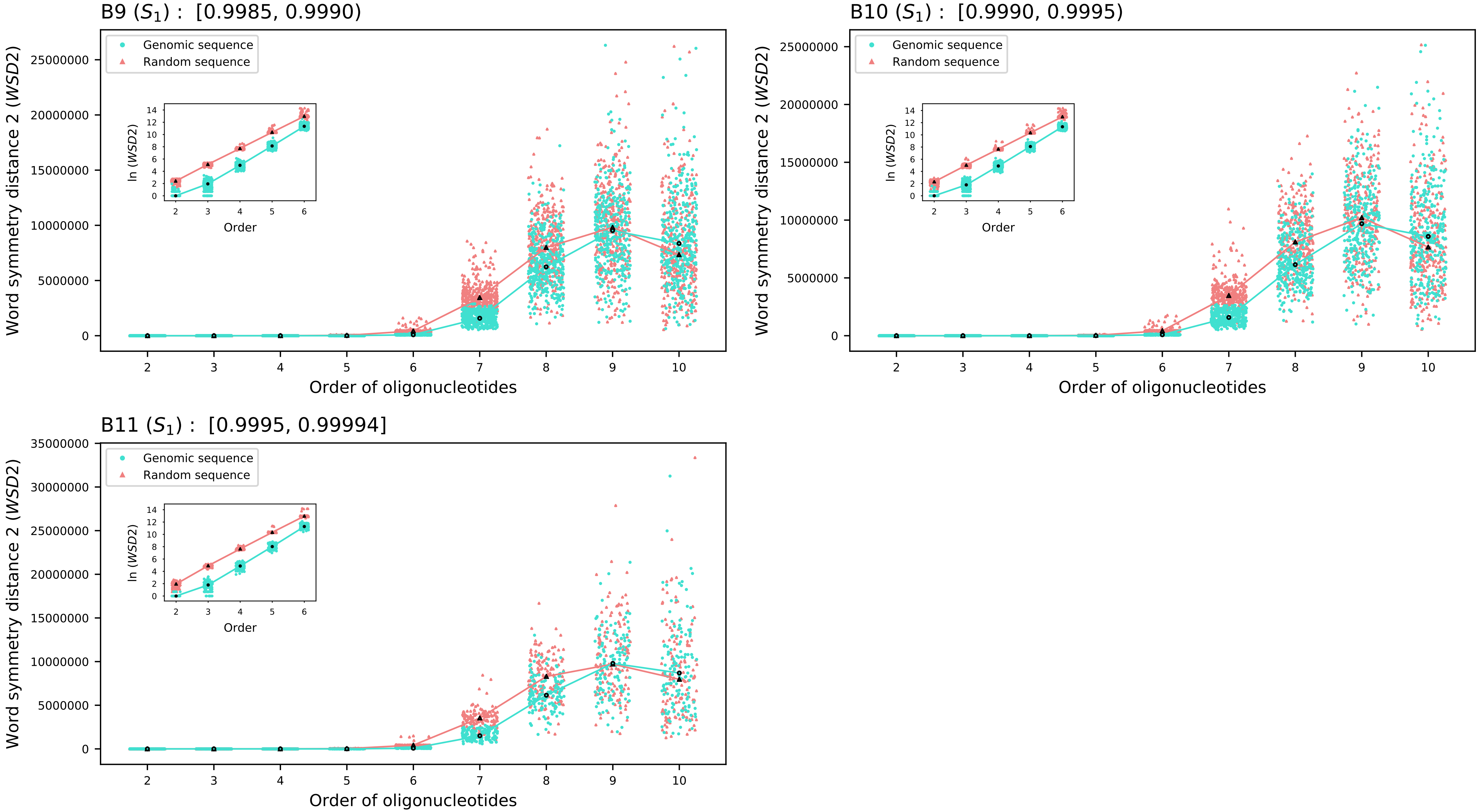

B9 (S_1) : [0.9985, 0.9990)
B10 (S_1) : [0.9990, 0.9995)
B11 (S_1) : [0.9995, 0.99994]
Genomic sequence
Random sequence
Word symmetry distance 2 ($WSD2$)
ln ($WSD2$)
Order
Order of oligonucleotides
