# Supplementary material for: Evaluation of the Persistence of Higher-Order Strand Symmetry in Genomic Sequences by Novel Word Symmetry Distance Analysis
Source: Front Genet. 2019 Mar 7;10:148. doi: 10.3389/fgene.2019.00148 (PMC6416199; doi:10.3389/fgene.2019.00148)
Supplement: Supplementary file 5 [file Data_Sheet_5.PDF]

Supplementary material 7-2-1. *WSD*1 for groups of genomes (classified according to phylum/class)

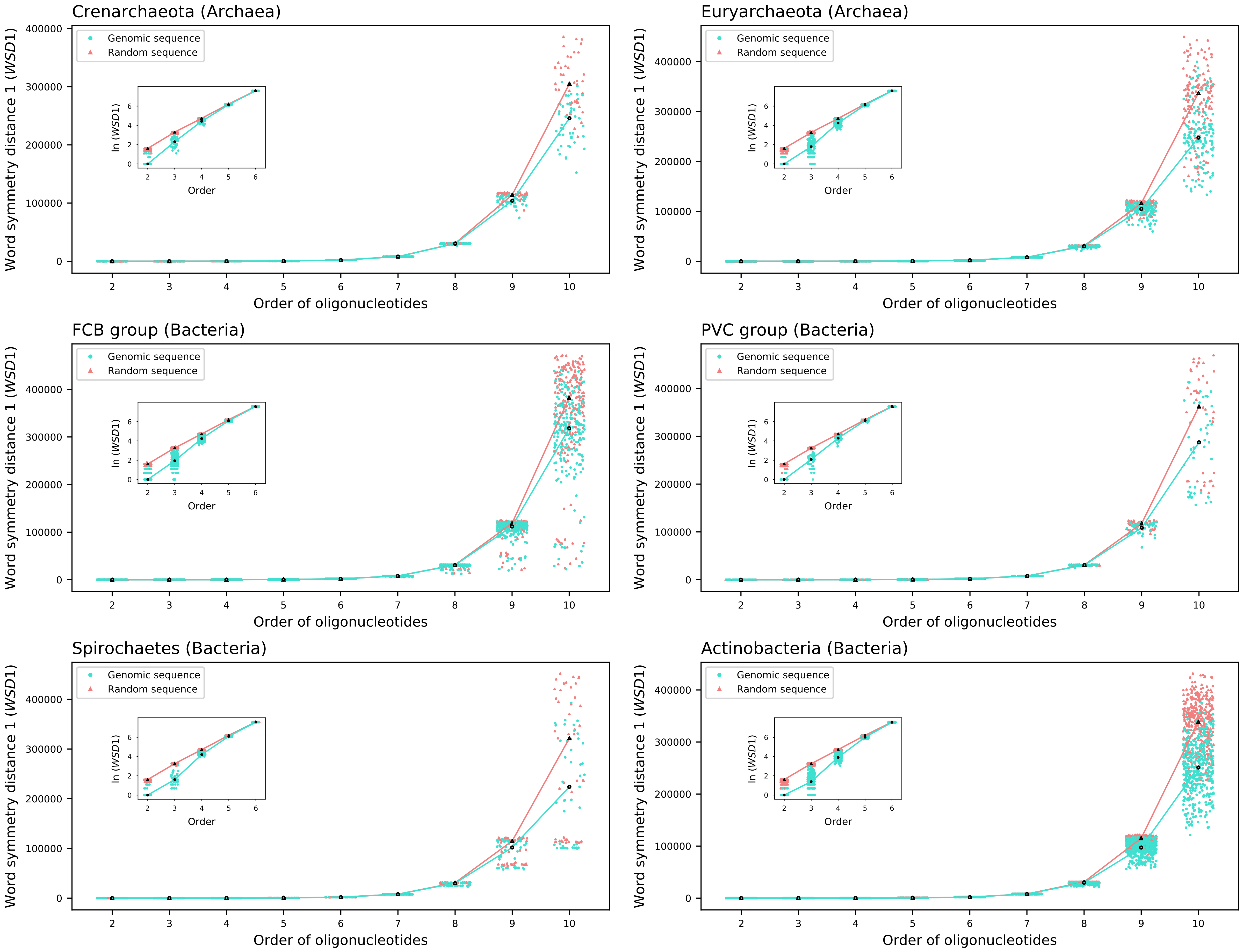

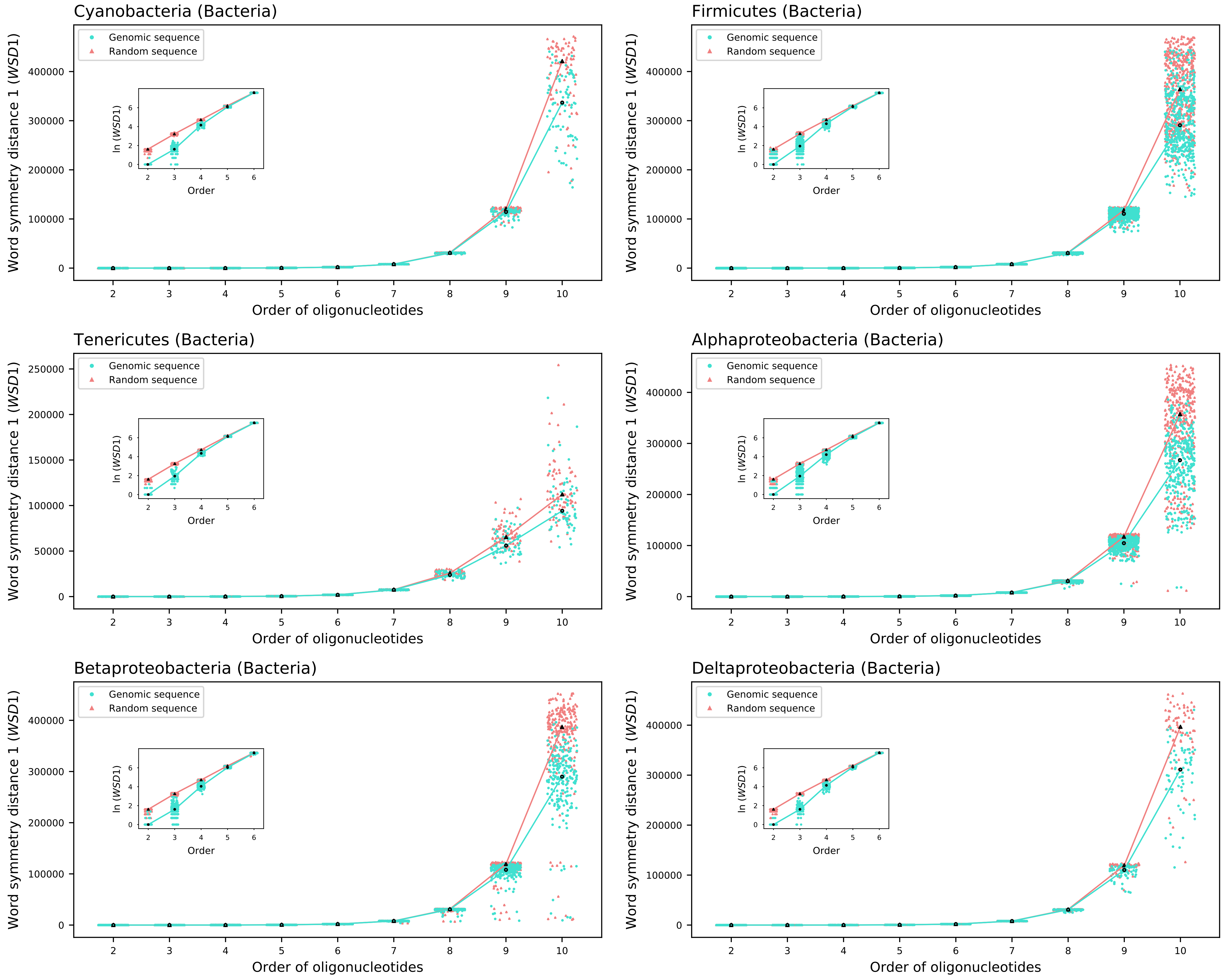

Cyanobacteria (Bacteria)
Firmicutes (Bacteria)
Tenericutes (Bacteria)
Alphaproteobacteria (Bacteria)
Betaproteobacteria (Bacteria)
Deltaproteobacteria (Bacteria)
Genomic sequence
Random sequence
Word symmetry distance 1 (WSD1)
Order of oligonucleotides
ln (WSD1)
Order

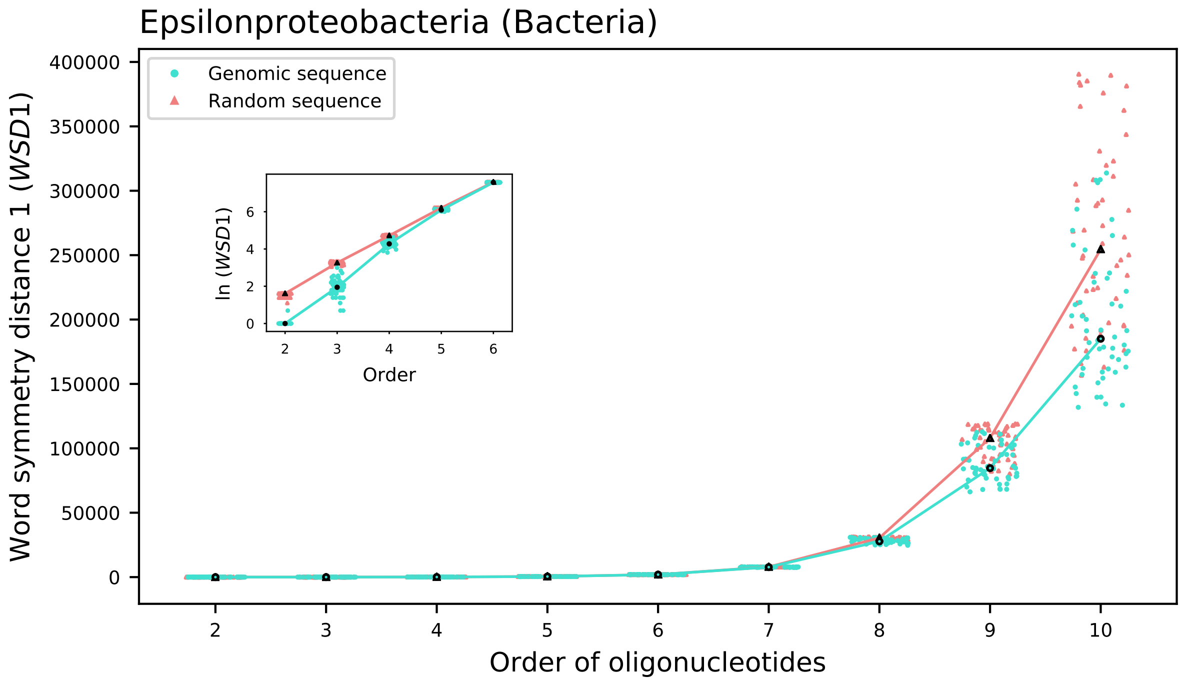

Epsilonproteobacteria (Bacteria)
Genomic sequence
Random sequence
Word symmetry distance 1 (WSD1)
ln (WSD1)
Order
Order of oligonucleotides

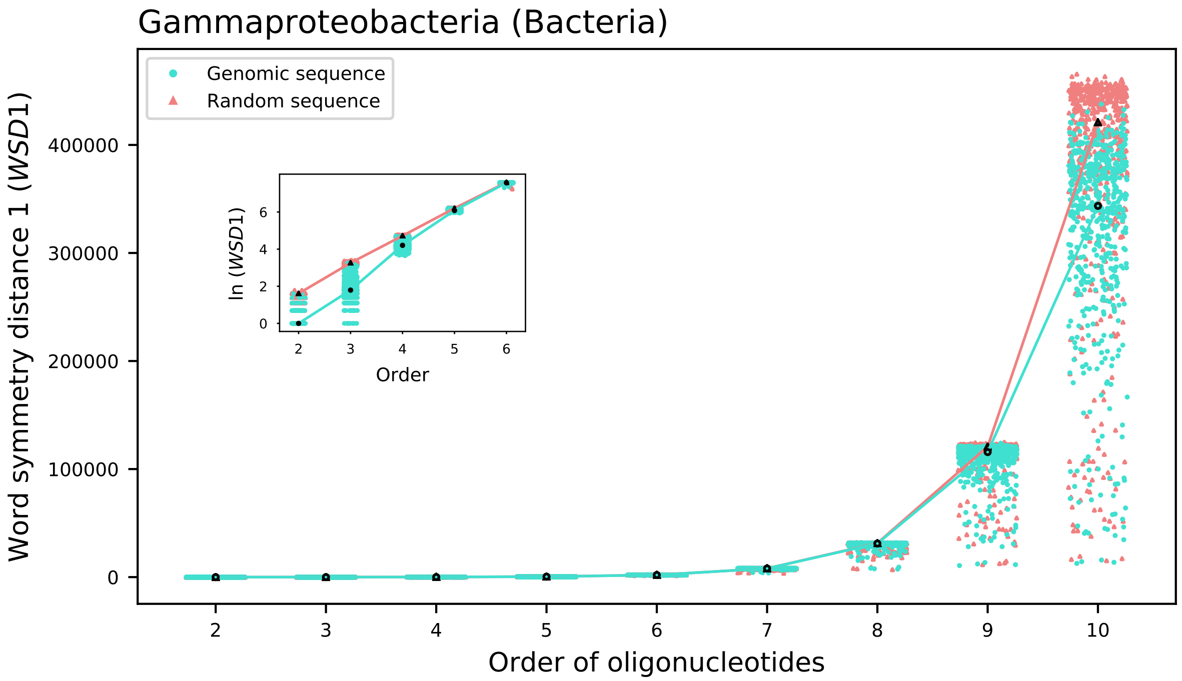

Gammaproteobacteria (Bacteria)
Genomic sequence
Random sequence
Word symmetry distance 1 (WSD1)
ln (WSD1)
Order
Order of oligonucleotides

Supplementary material 7-2-2. *WSD*2 for groups of genomes (classified according to phylum/class)

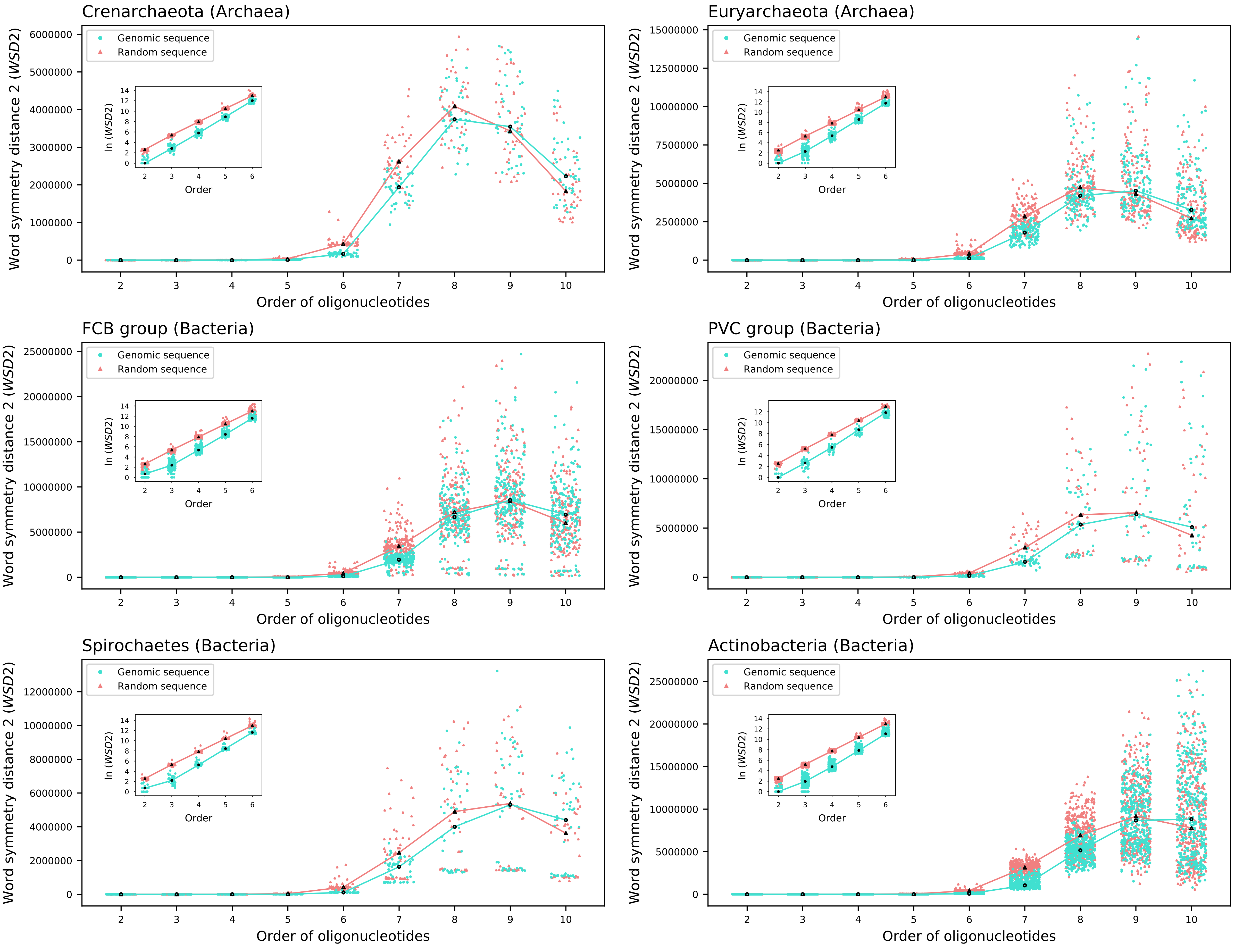

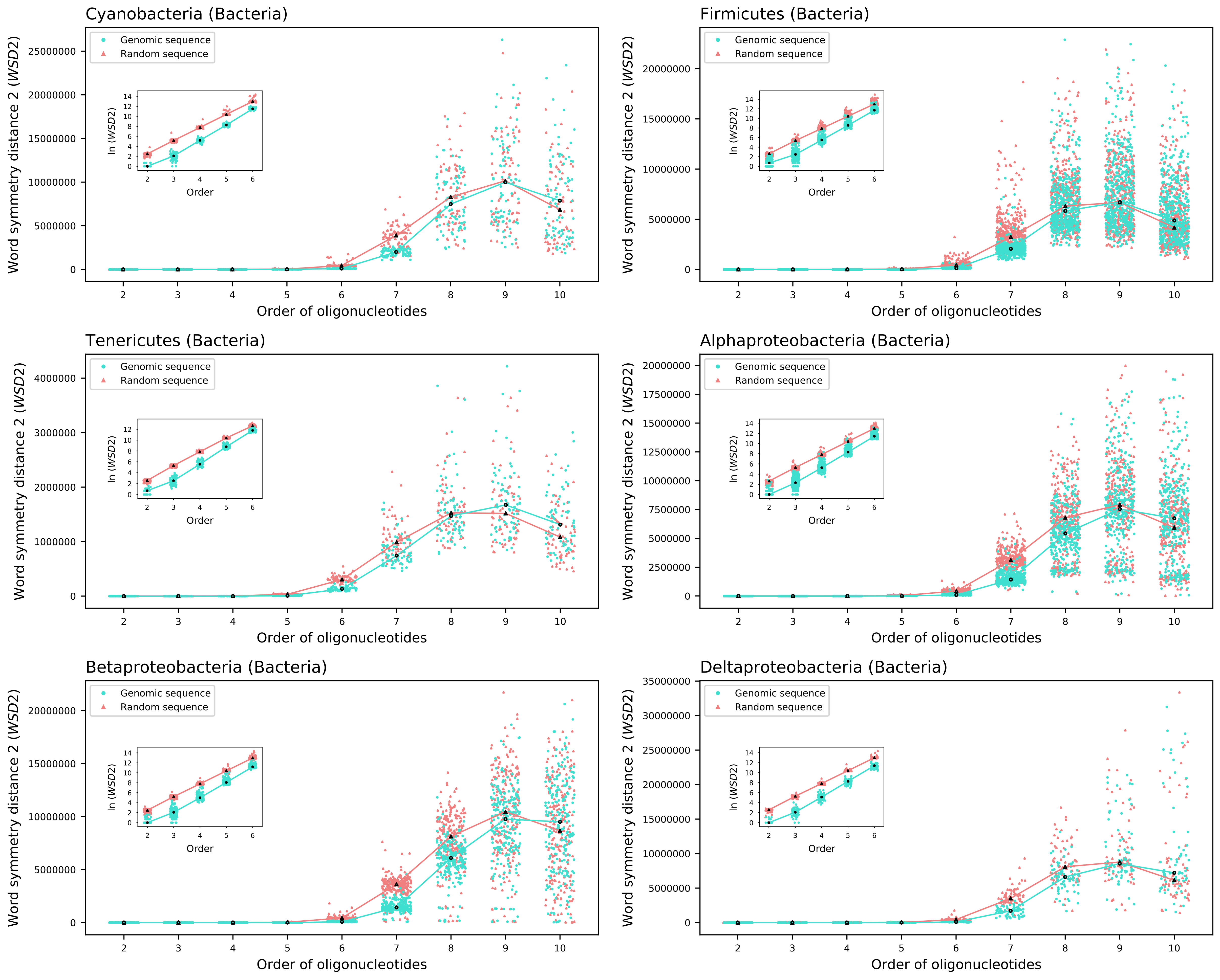

Cyanobacteria (Bacteria)
Firmicutes (Bacteria)
Tenericutes (Bacteria)
Alphaproteobacteria (Bacteria)
Betaproteobacteria (Bacteria)
Deltaproteobacteria (Bacteria)
Genomic sequence
Random sequence
Word symmetry distance 2 (WSD2)
Order of oligonucleotides
ln (WSD2)
Order

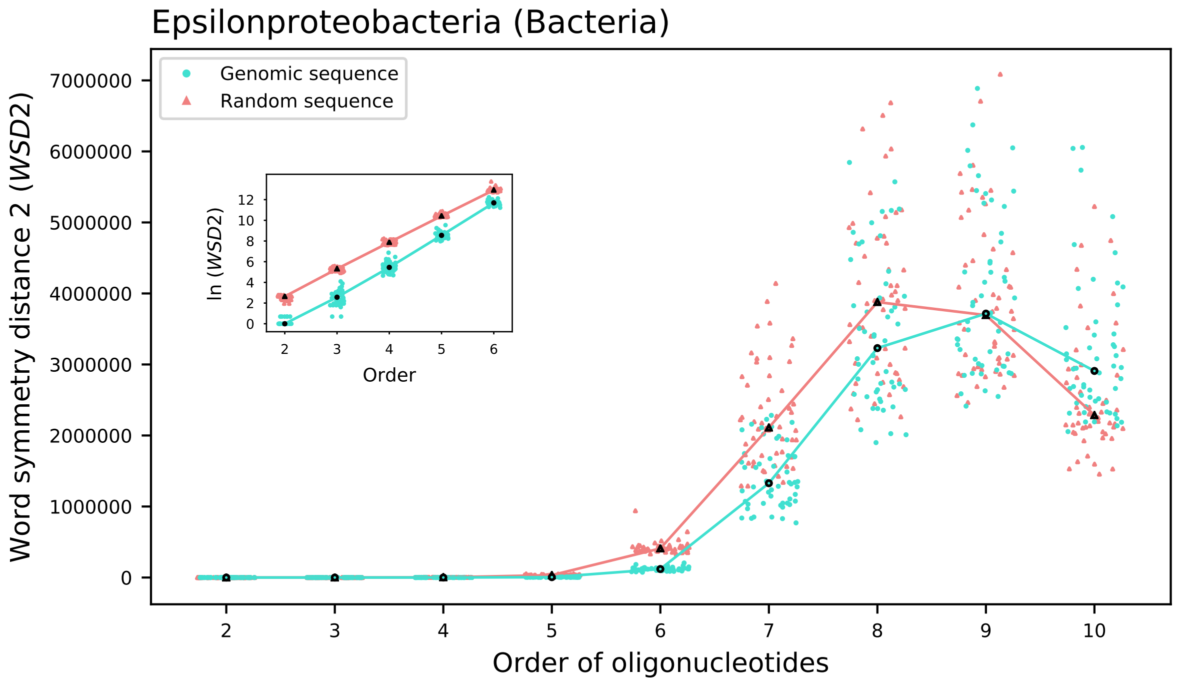

Epsilonproteobacteria (Bacteria)
Genomic sequence
Random sequence
Word symmetry distance 2 (WSD2)
ln (WSD2)
Order
Order of oligonucleotides

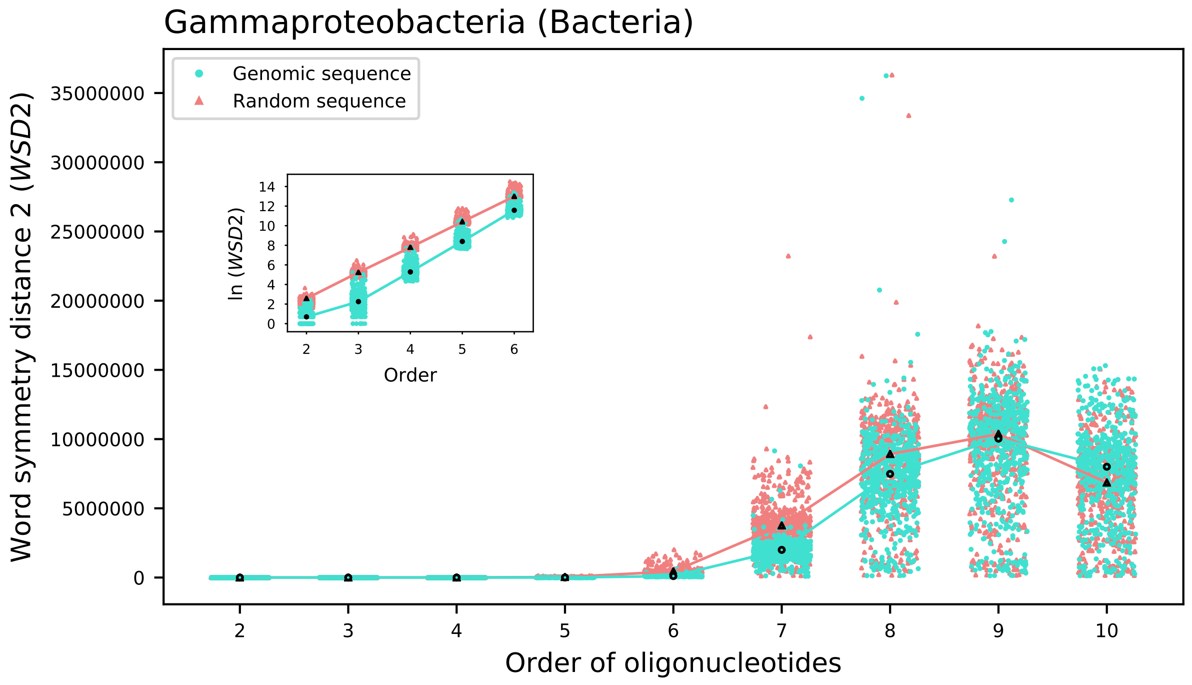

Gammaproteobacteria (Bacteria)
Genomic sequence
Random sequence
Word symmetry distance 2 (WSD2)
ln (WSD2)
Order
Order of oligonucleotides
