# Supplementary material for: Evaluation of the Persistence of Higher-Order Strand Symmetry in Genomic Sequences by Novel Word Symmetry Distance Analysis
Source: Front Genet. 2019 Mar 7;10:148. doi: 10.3389/fgene.2019.00148 (PMC6416199; doi:10.3389/fgene.2019.00148)
Supplement: Supplementary file 6 [file Data_Sheet_6.PDF]

Supplementary material 8-1. Distribution of GC content in groups of genomes by phylum/class

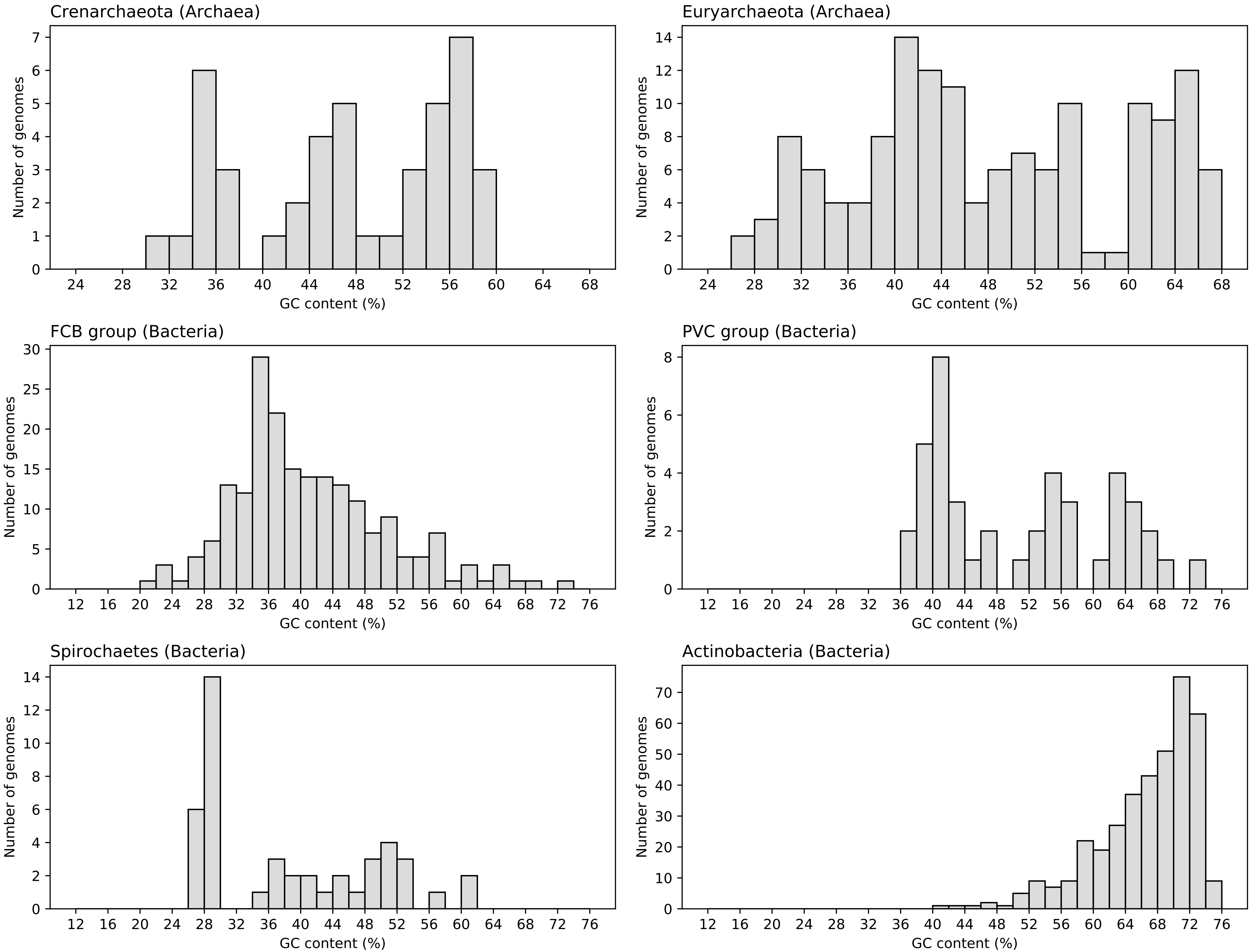

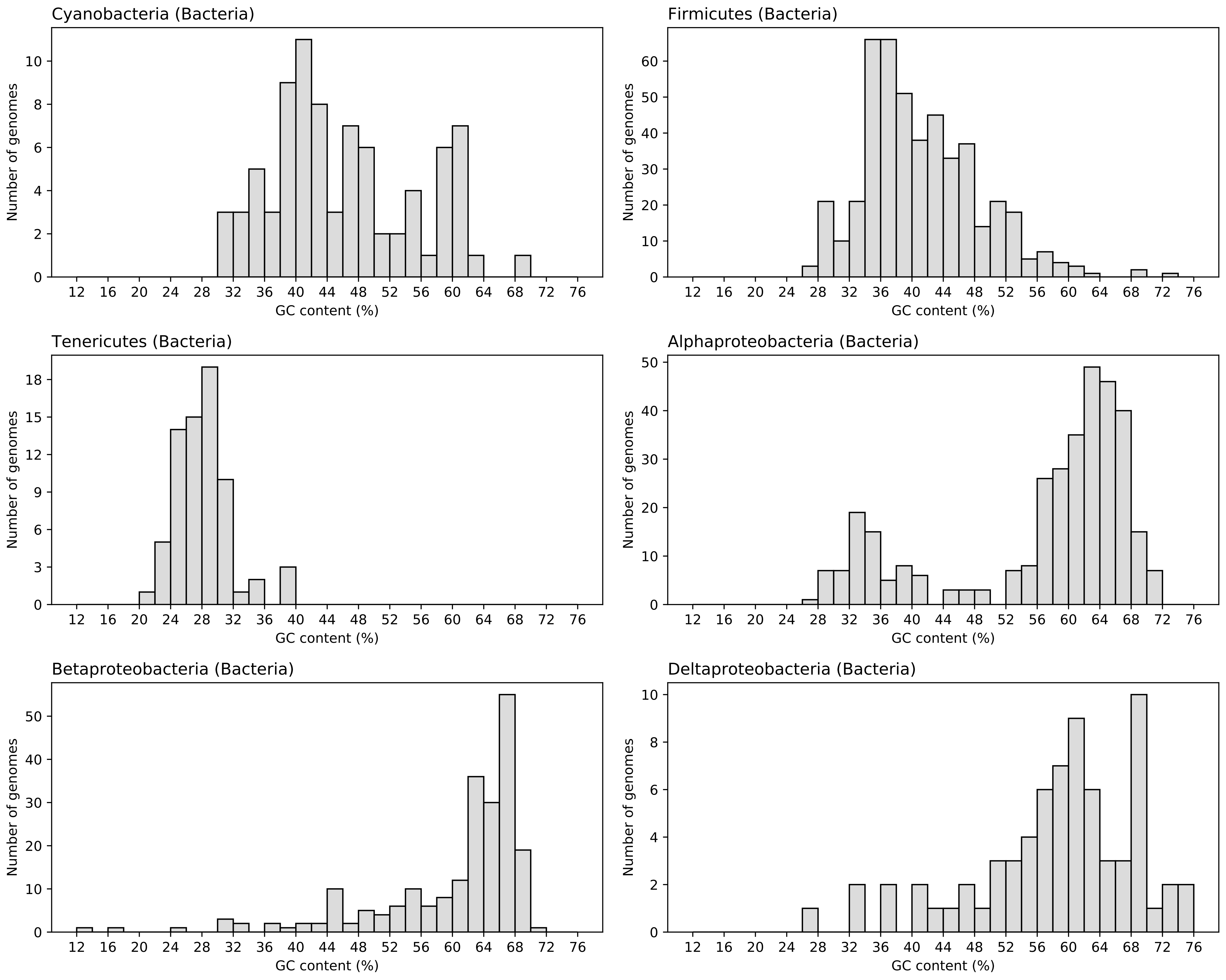
Cyanobacteria (Bacteria)
Firmicutes (Bacteria)
Tenericutes (Bacteria)
Alphaproteobacteria (Bacteria)
Betaproteobacteria (Bacteria)
Deltaproteobacteria (Bacteria)
Number of genomes
GC content (%)
12 16 20 24 28 32 36 40 44 48 52 56 60 64 68 72 76

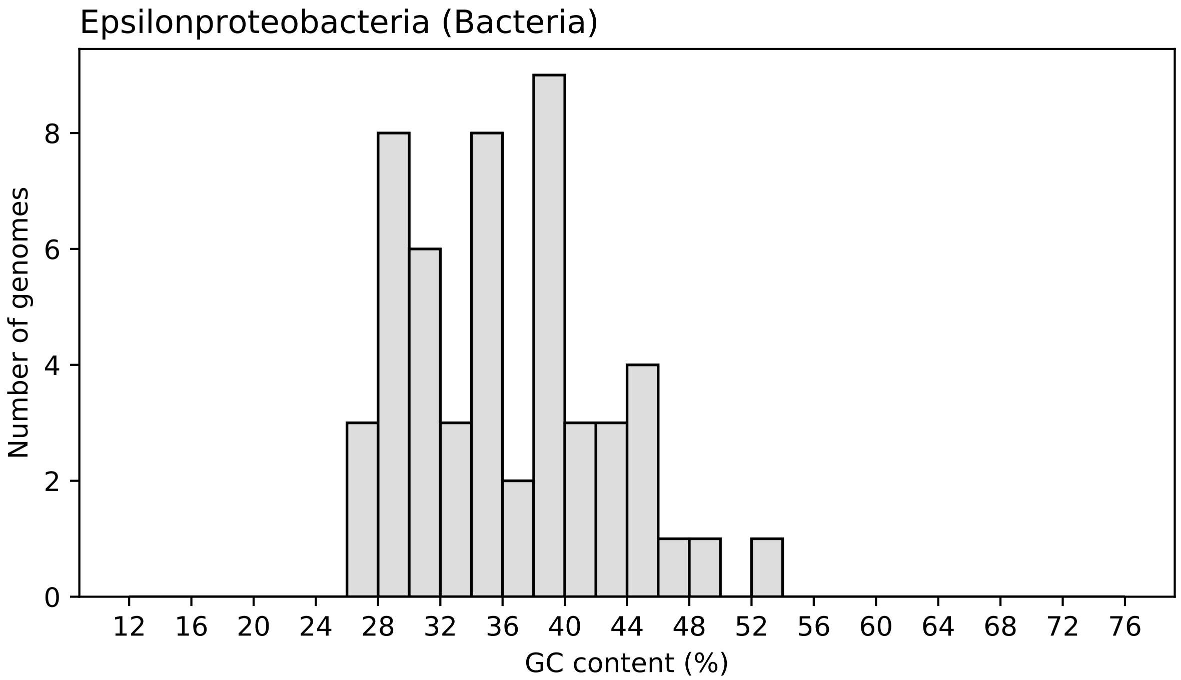
Epsilonproteobacteria (Bacteria)
Number of genomes
GC content (%)
0
2
4
6
8
12
16
20
24
28
32
36
40
44
48
52
56
60
64
68
72
76

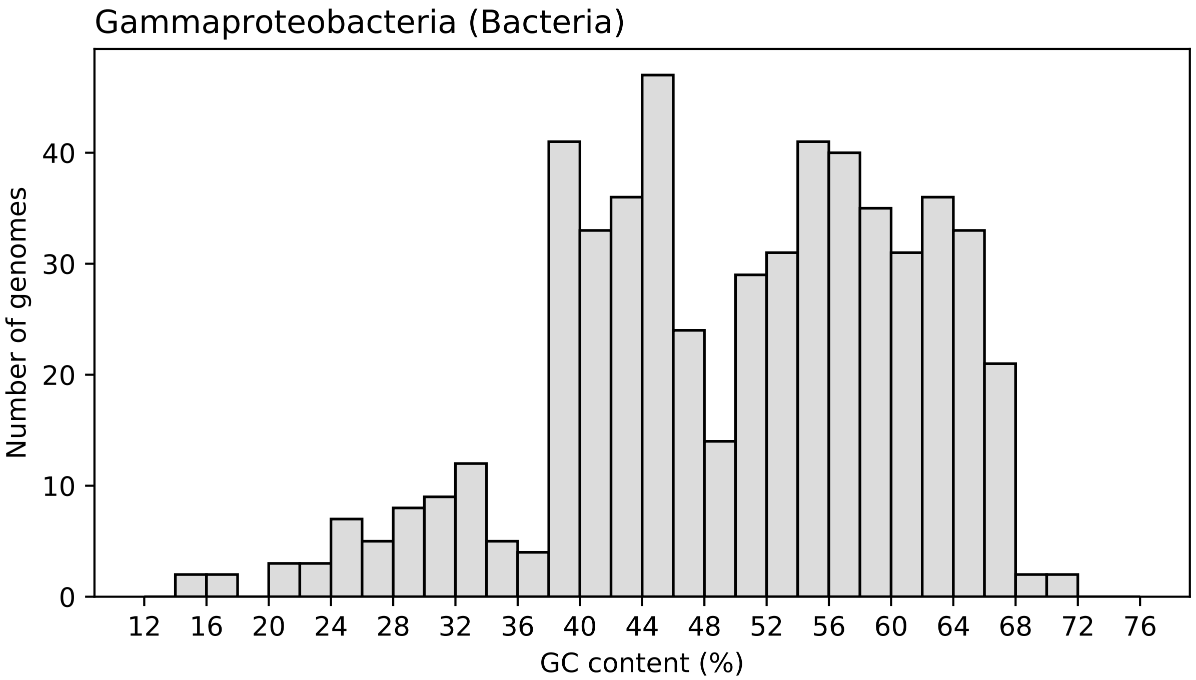
Gammaproteobacteria (Bacteria)
Number of genomes
GC content (%)
0
10
20
30
40
12
16
20
24
28
32
36
40
44
48
52
56
60
64
68
72
76

Supplementary material 8-2. Distribution of S_1 in groups of genomes by phylum/class

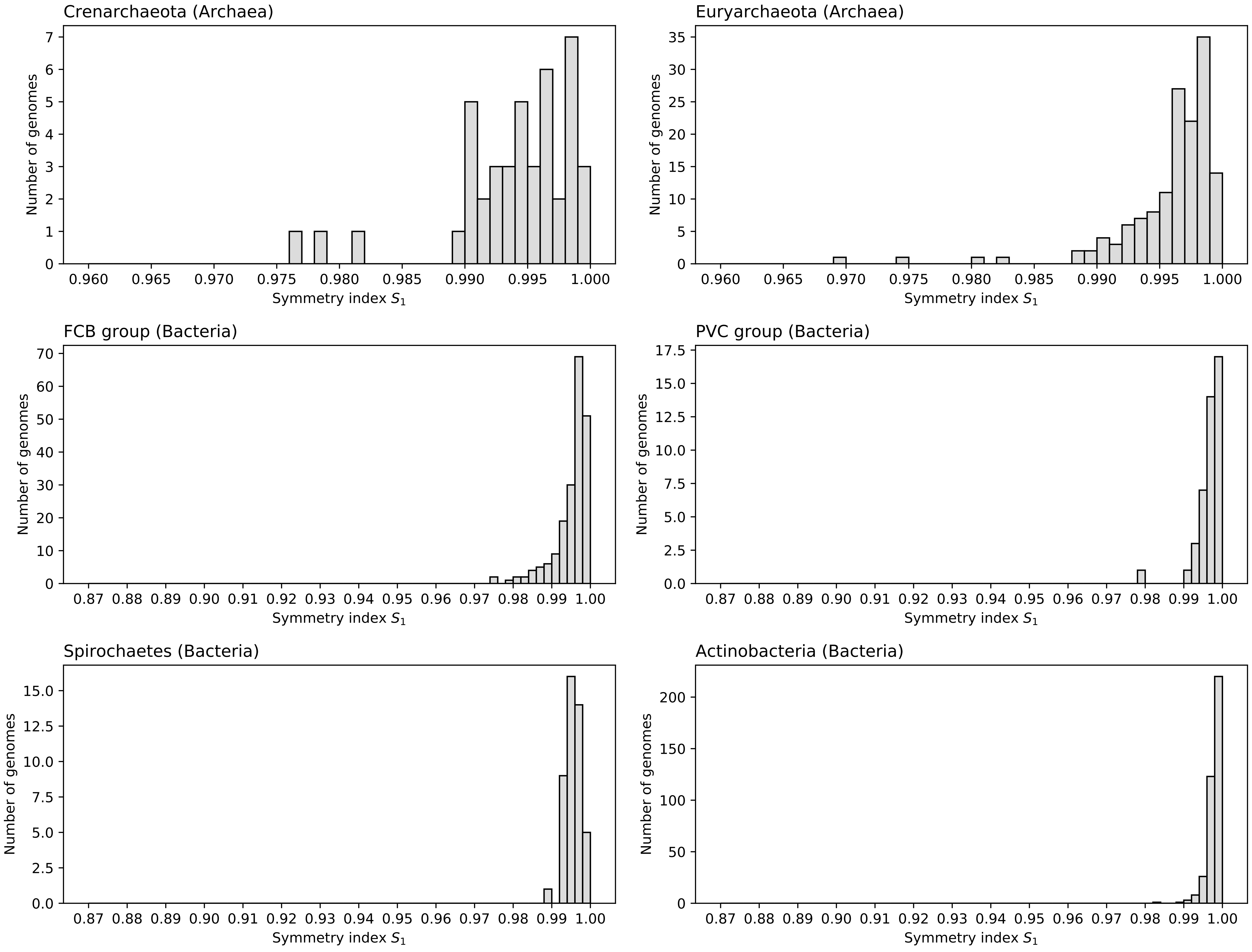

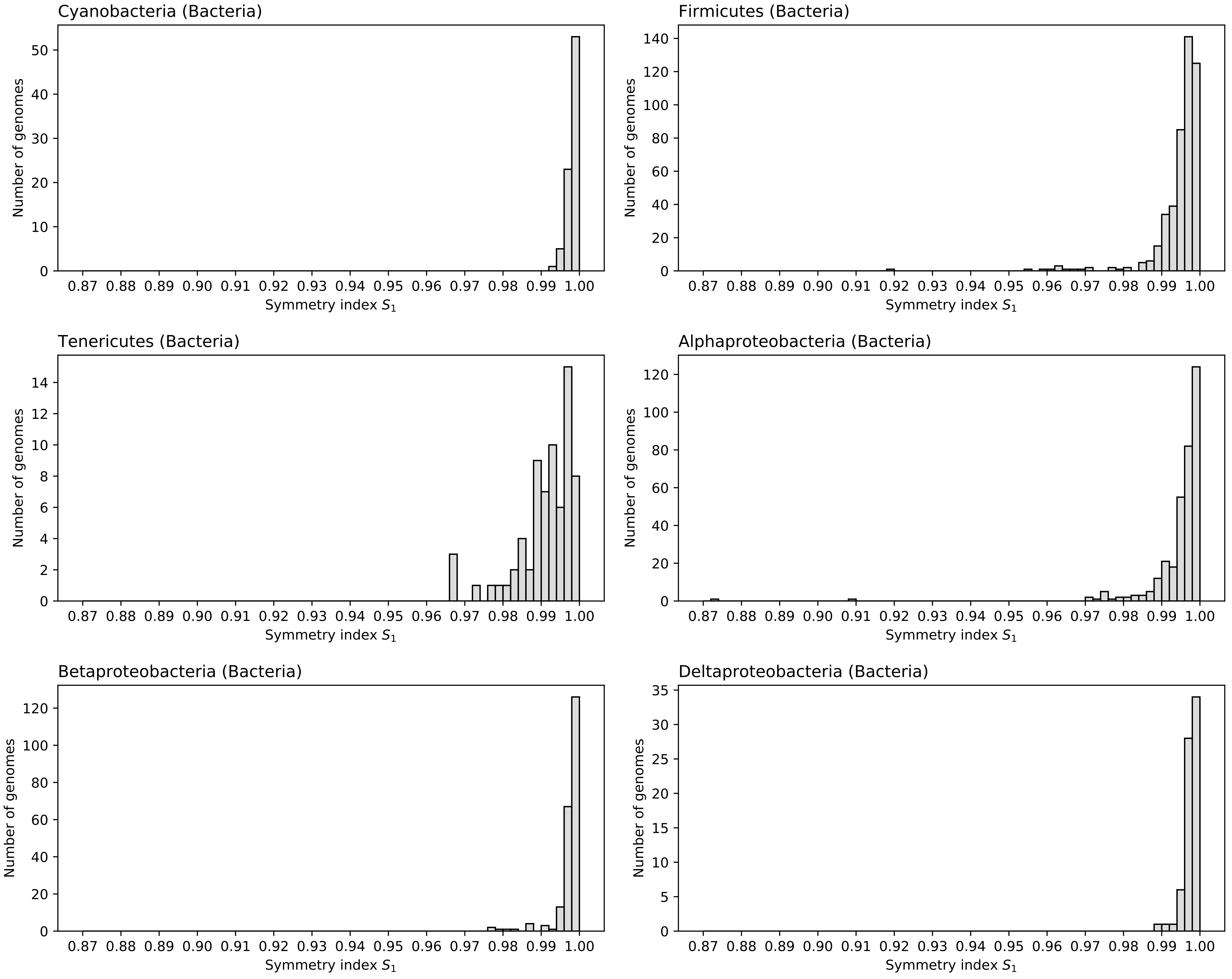

Cyanobacteria (Bacteria)
Number of genomes
0
10
20
30
40
50
0.87
0.88
0.89
0.90
0.91
0.92
0.93
0.94
0.95
0.96
0.97
0.98
0.99
1.00
Symmetry index S_1
Firmicutes (Bacteria)
Number of genomes
0
20
40
60
80
100
120
140
Symmetry index S_1
Tenericutes (Bacteria)
Number of genomes
0
2
4
6
8
10
12
14
Symmetry index S_1
Alphaproteobacteria (Bacteria)
Number of genomes
0
20
40
60
80
100
120
Symmetry index S_1
Betaproteobacteria (Bacteria)
Number of genomes
0
20
40
60
80
100
120
Symmetry index S_1
Deltaproteobacteria (Bacteria)
Number of genomes
0
5
10
15
20
25
30
35
Symmetry index S_1

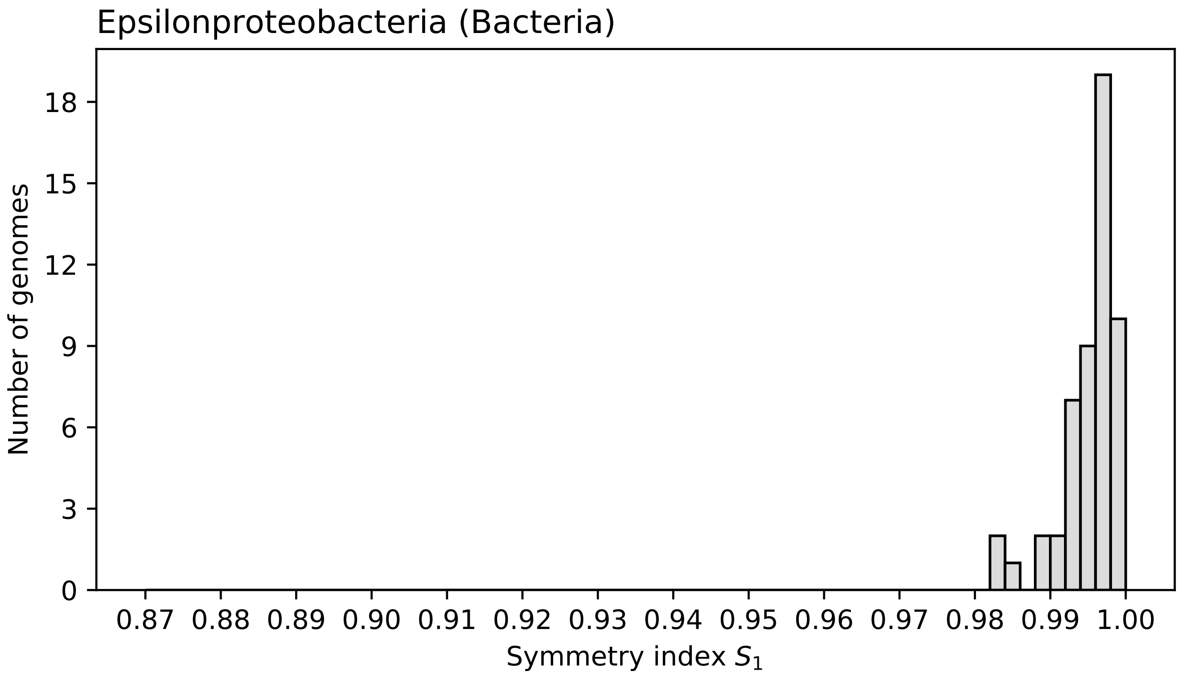
Epsilonproteobacteria (Bacteria)
Number of genomes
Symmetry index S_1
0.87
0.88
0.89
0.90
0.91
0.92
0.93
0.94
0.95
0.96
0.97
0.98
0.99
1.00
0
3
6
9
12
15
18

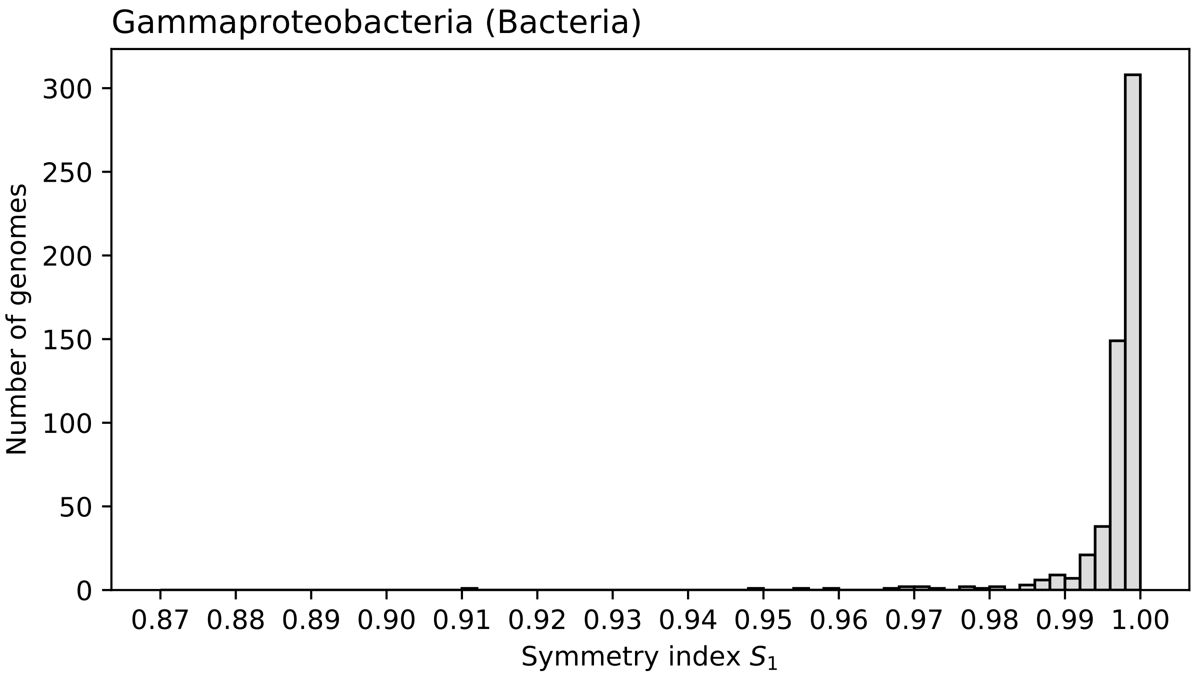
Gammaproteobacteria (Bacteria)
Number of genomes
Symmetry index S_1
0.87
0.88
0.89
0.90
0.91
0.92
0.93
0.94
0.95
0.96
0.97
0.98
0.99
1.00
0
50
100
150
200
250
300
