# Supplementary material for: Evaluation of the Persistence of Higher-Order Strand Symmetry in Genomic Sequences by Novel Word Symmetry Distance Analysis
Source: Front Genet. 2019 Mar 7;10:148. doi: 10.3389/fgene.2019.00148 (PMC6416199; doi:10.3389/fgene.2019.00148)
Supplement: Supplementary file 7 [file Data_Sheet_7.PDF]

Supplementary material 9-2. Word symmetry distance 2 (*WSD*2) for individual genomes

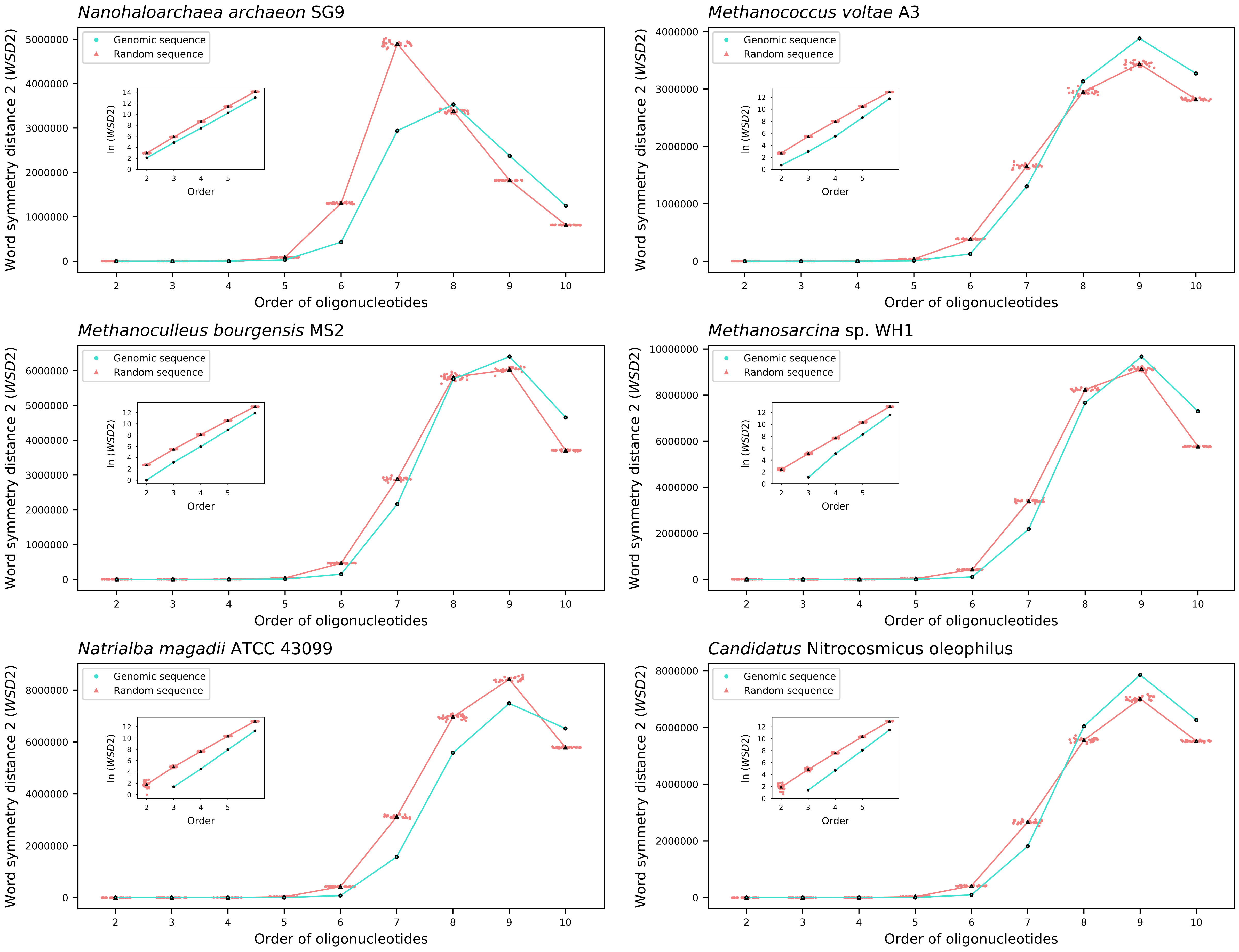

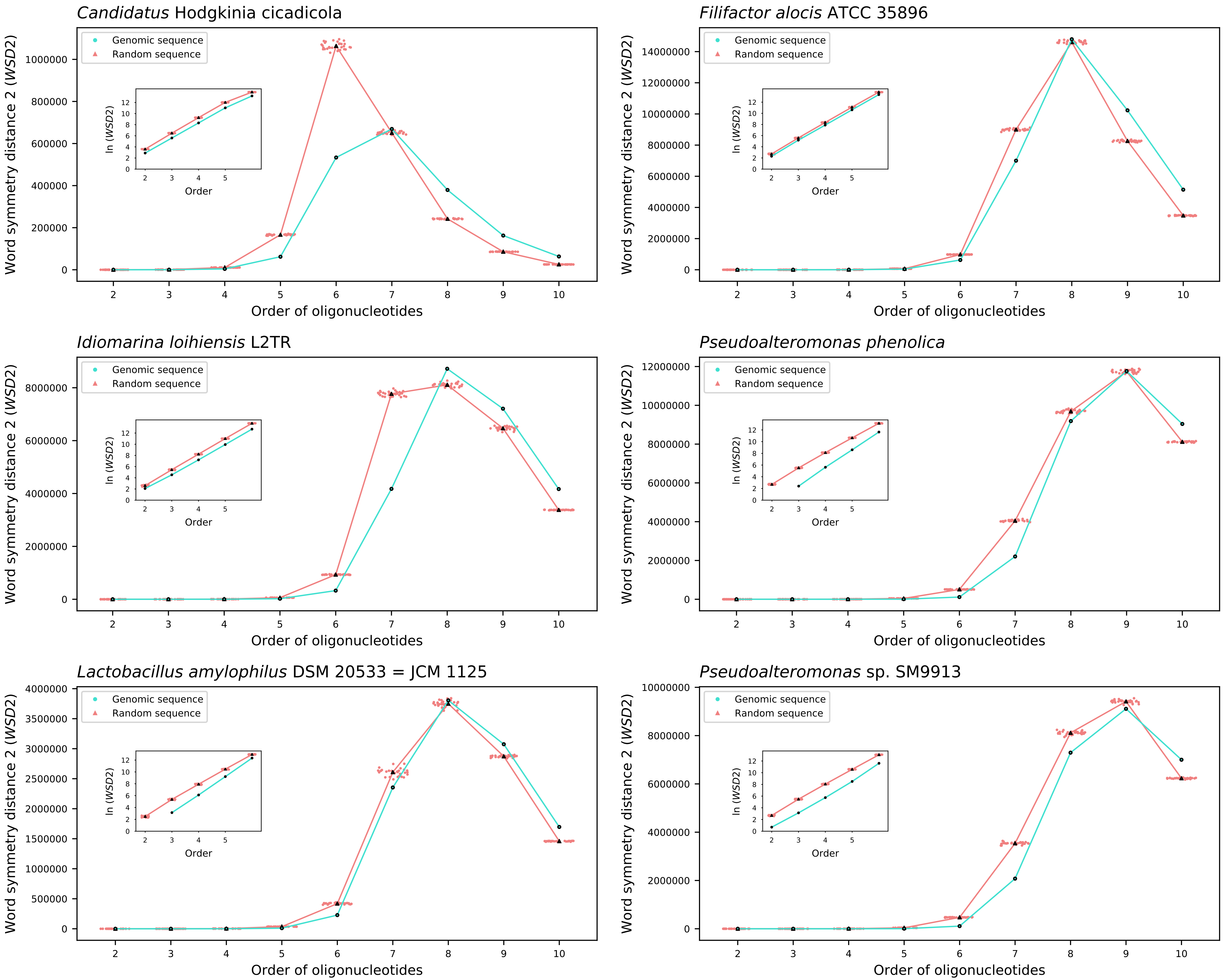

Candidatus Hodgkinia cicadicola
Filifactor alocis ATCC 35896
Idiomarina loihiensis L2TR
Pseudoalteromonas phenolica
Lactobacillus amylophilus DSM 20533 = JCM 1125
Pseudoalteromonas sp. SM9913
Genomic sequence
Random sequence
Word symmetry distance 2 (*WSD2*)
Order of oligonucleotides
ln (*WSD2*)
Order

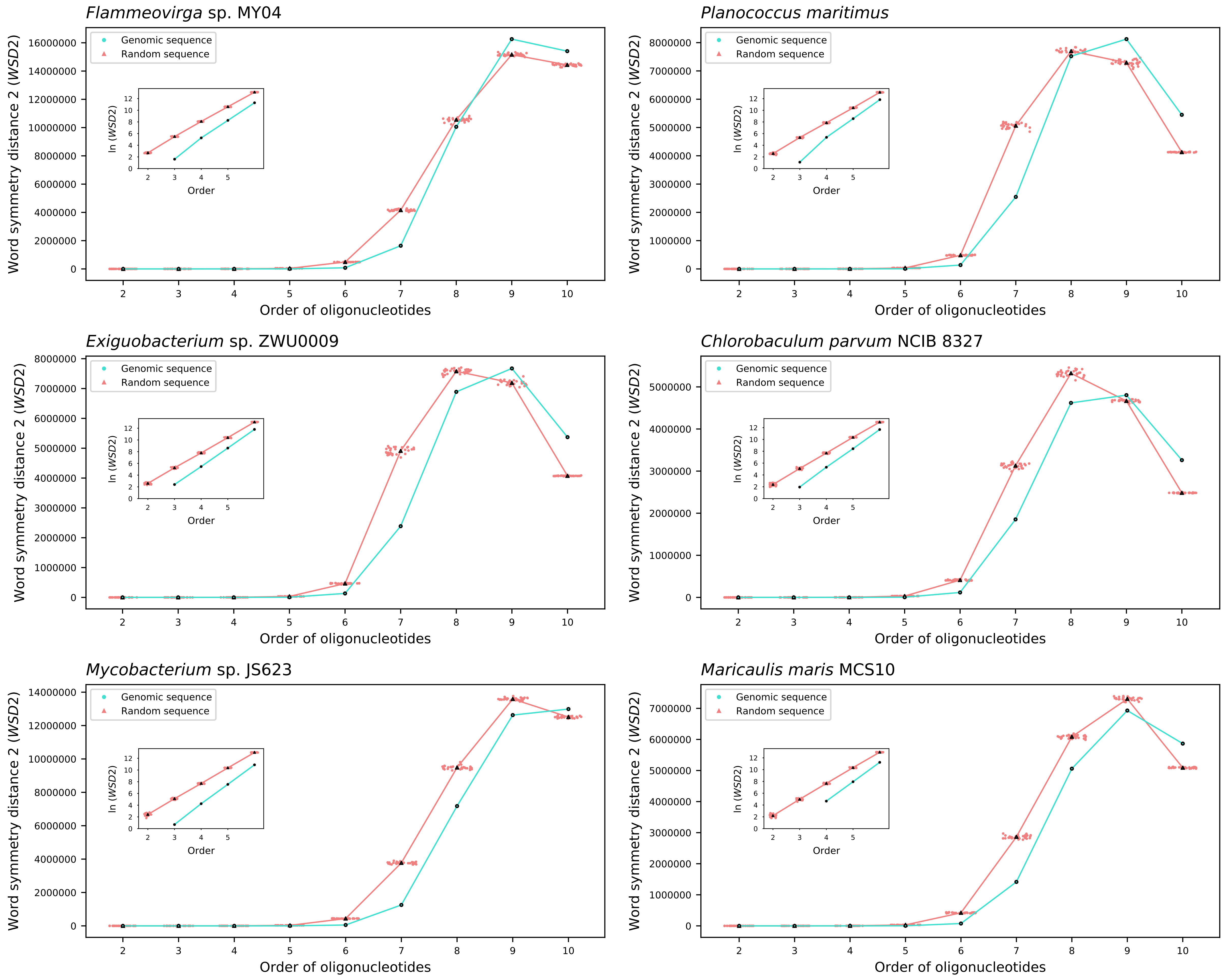
Flammeovirga sp. MY04
Planococcus maritimus
Exiguobacterium sp. ZWU0009
Chlorobaculum parvum NCIB 8327
Mycobacterium sp. JS623
Maricaulis maris MCS10
Genomic sequence
Random sequence
Word symmetry distance 2 (WSD2)
Order of oligonucleotides
ln (WSD2)
Order

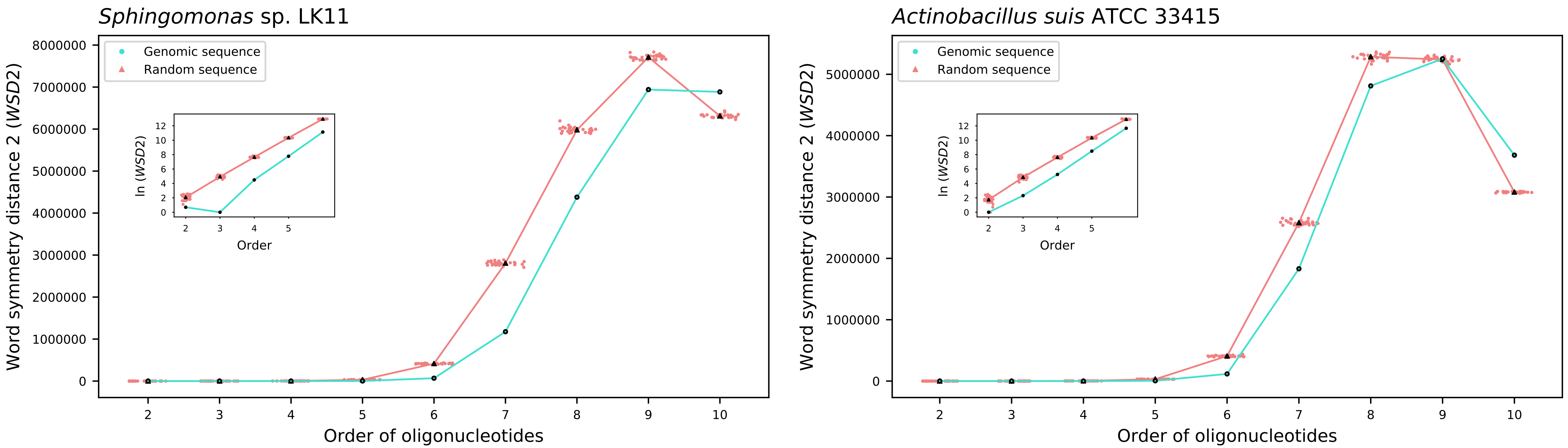

Sphingomonas sp. LK11
Genomic sequence
Random sequence
Word symmetry distance 2 (WSD2)
ln (WSD2)
Order
Order of oligonucleotides
Actinobacillus suis ATCC 33415
Genomic sequence
Random sequence
Word symmetry distance 2 (WSD2)
ln (WSD2)
Order
Order of oligonucleotides
